# Enantioselective allylic alkylation of stereodefined polysubstituted copper enolates as an entry to acyclic quaternary carbon stereocentres†
†Electronic supplementary information (ESI) available. CCDC 1488151. For ESI and crystallographic data in CIF or other electronic format see DOI: 10.1039/c6sc03036j



**DOI:** 10.1039/c6sc03036j

**Published:** 2016-09-15

**Authors:** Zackaria Nairoukh, Gunda G. K. S. Narayana Kumar, Yury Minko, Ilan Marek

**Affiliations:** a The Mallat Family Laboratory of Organic Chemistry , Schulich Faculty of Chemistry and Lise Meitner-Minerva Center for Computational Quantum Chemistry , Technion-Israel Institute of Technology , Technion City , Haifa 32000 , Israel . Email: chilanm@tx.technion.ac.il ; Fax: +972-4-829-37-09 ; Tel: +972-4-829-37-09

## Abstract

A sequence of regio- and stereoselective carbometalation followed by oxidation of ynamides leads to stereodefined fully substituted enolates that subsequently react with various functionalized allyl bromide reagents to provide the expected products possessing an enantiomerically pure quaternary carbon stereocentre in the α-position to the carbonyl group in excellent yields and enantiomeric ratios after cleavage of the oxazolidinone moiety. Three new bonds are formed in a single-pot operation.

## Abstract 2

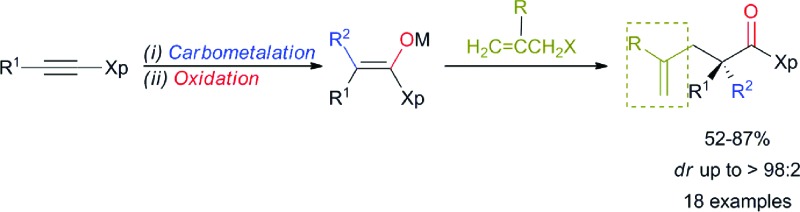
A sequence of regio- and stereoselective carbometalation followed by oxidation of ynamides leads to stereodefined fully substituted enolates that subsequently react with various functionalized allyl bromide reagents to provide the enantiomerically pure quaternary carbon stereocentre in the α-position to the carbonyl group.

## Introduction

**Scheme 1:**
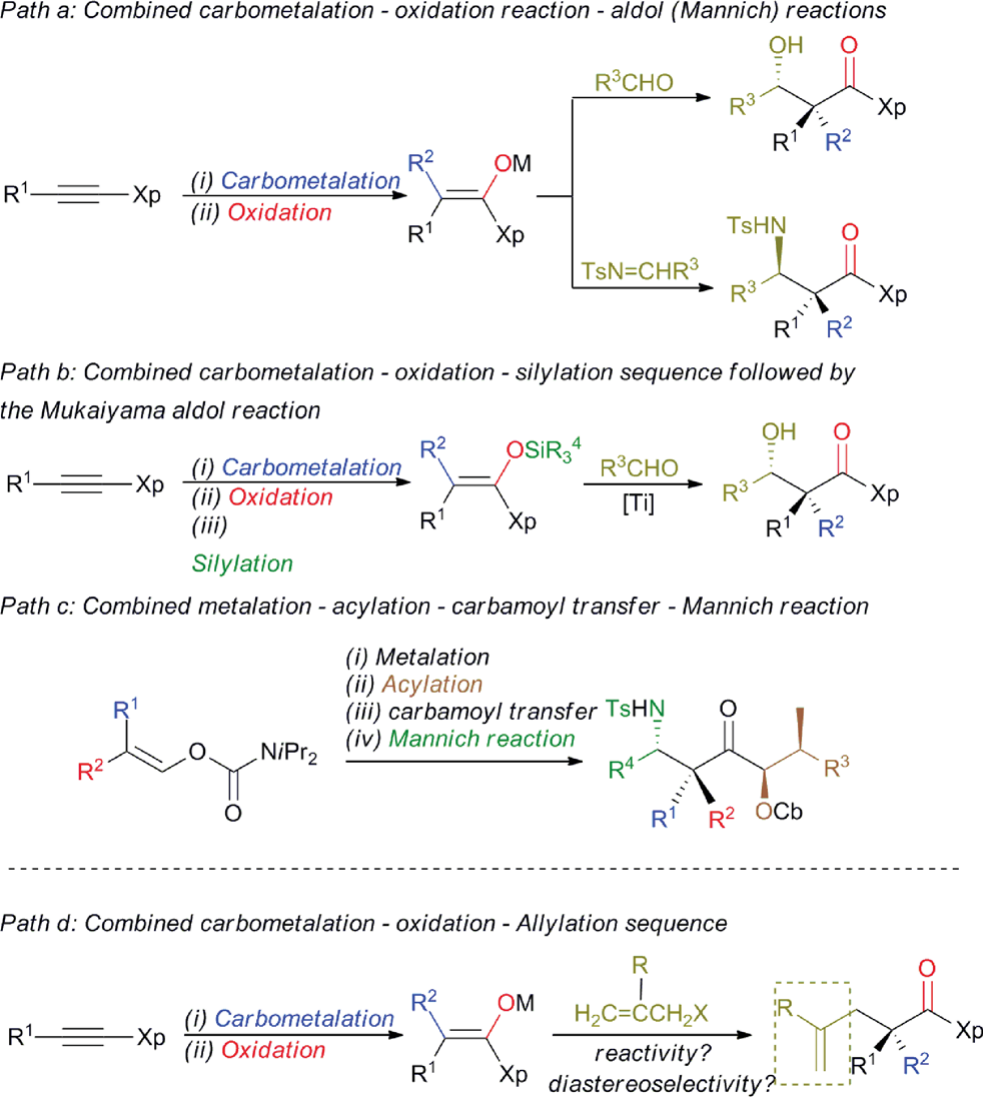
Formation of quaternary carbon stereocentres from alkynes and alkenes. Xp = chiral auxiliary.

The enantioselective synthesis of a quaternary carbon stereocentre α to a carbonyl group in acyclic systems is an ongoing challenge in the field of stereoselective synthesis, given the prevalence of these centres in a wide variety of natural products with important structural and biological properties.^1^ Even more challenging would be the preparation of these stereocentres from common starting materials with the concomitant creation of several new bonds in a single-pot operation.^2^ Additionally, any development of a new strategy that would answer this synthetic goal should be flexible enough to allow the direct preparation of functionalized quaternary stereocentres to avoid protection-deprotection steps.^3^ One of the main issues that has limited the formation of these stereocentres has been the lack of practical approaches to generate stereodefined acyclic β,β-disubstituted enolates.^4^ In this context, we have previously reported a simple, efficient and reliable stereoselective approach to polysubstituted stereodefined enolate species based on a carbometalation reaction of α-heterosubstituted alkynes followed by a selective *in situ* oxidation reaction.^5^ This approach was proven to be synthetically useful as it was successfully applied to the preparation of aldol and Mannich-type products in good overall yields and excellent diastereoselectivities (Scheme 1, path a).^6^ Alternatively, the aldol reaction with aliphatic aldehydes could be subsequently achieved by using stereodefined disubstituted silyl ketene aminals through the Mukaiyama aldol reaction (Scheme 1, path b).^7^ More recent studies have also shown that the preparation of stereochemically defined acyclic fully substituted enolates of ketones is now also possible from simple vinyl carbamates, and this has been successfully used in aldol and Mannich-type transformations (Scheme 1, path c).^8^

Although these methodologies are now well established, the formation of these quaternary stereocentres is always accompanied by the creation of an additional adjacent stereocentre.

## Results and discussion

The preparation of an acyclic single quaternary stereocentre devoid of any additional new ones would be highly desirable in the context of natural product synthesis.^9^ One potential solution to this question could emerge from the diastereoselective allylation of stereodefined polysubstituted metal enolates (Scheme 1, path d).^10^ It should be noted that the presence of this allylic functionality could be subsequently manipulated towards the synthesis of more complex molecular architectures. Herein, we disclose our approach for the diastereo- and enantioselective formation of quaternary stereocentres α to a carbonyl functionality *via* the allylation of stereodefined β,β-disubstituted amide copper enolates.

Stereodefined polysubstituted enolates could be formed through the addition of an organocuprate R_2_^2^CuLi·Me_2_S, easily accessible by the addition of 2 equivalents of commercially available R^2^Li to 1 equivalent of CuBr·SMe_2_,^11^ to ynamide **1**.^12,13^ 1 Equivalent of *t*-BuOOH was then added to the formed vinyl cuprate **2**_Cuate_, and the acidic hydrogen of the peroxide was deprotonated *in situ* by the remaining sacrificial R^2^ group of the vinyl copper species to produce an oxenoid as a reactive intermediate,^14^ which could undergo a 1,2-metalate rearrangement^5,15^ to give stereodefined copper enolate **3** (method A). Alternatively, the addition of an organocopper R^2^Cu·Me_2_S, now obtained by the addition of only one equivalent of the same commercially available R^2^Li to 1 equivalent of CuBr·SMe_2_,^11^ to ynamide **1** led to the formation of the stereodefined vinyl copper species **2**_Cu_.

**Scheme 2:**
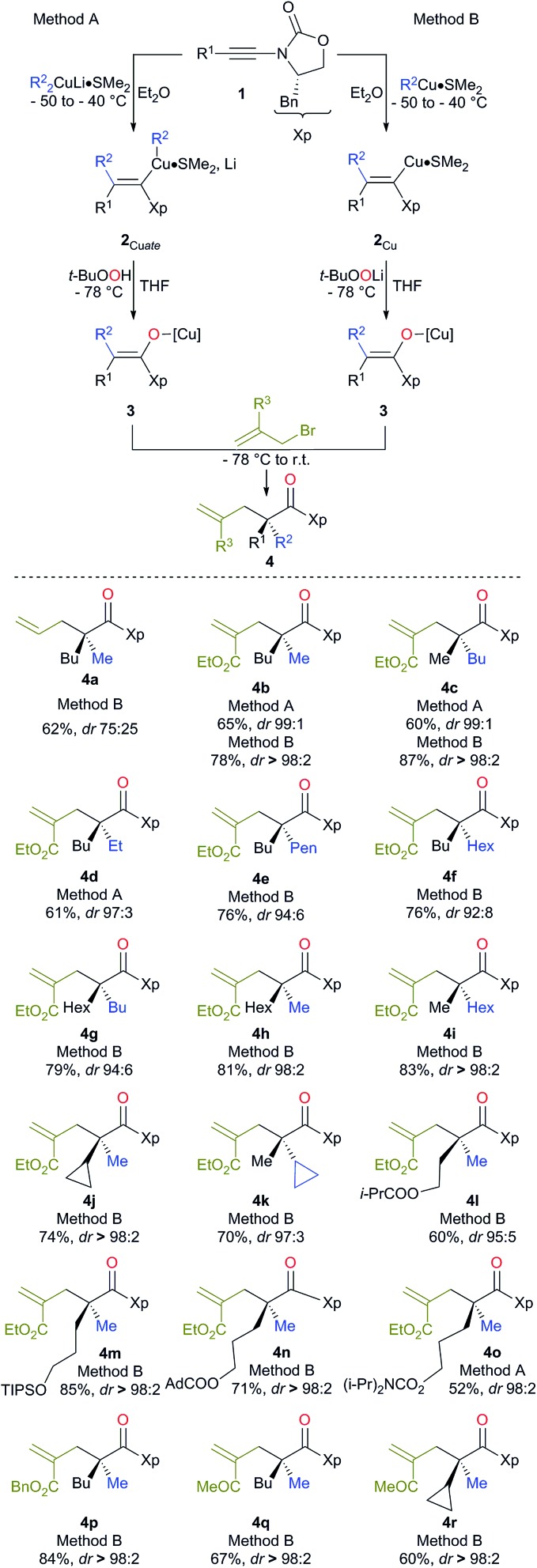
Formation of allylic alkylation products with quaternary carbon stereocentres.

Treatment of the resulting **2**_Cu_ with freshly prepared oxenoid *t*-BuOOLi led to the formation of the same stereodefined copper enolate **3** after the 1,2-metalate rearrangement (method B). Then, addition at low temperature of various allyl bromide derivatives to the reaction mixture produced the expected allylated products **4** as described in Scheme 2.

**Scheme 3:**
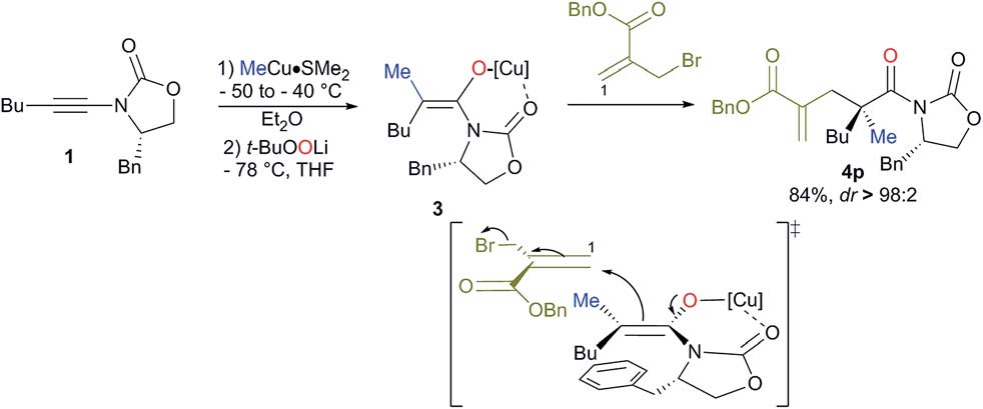
Proposed stereochemical model for the allylation reaction.

Although the addition of allyl bromide did not provide the expected product, even in the presence of various polar coordinative additives such as HMPA, DMPU, TMEDA, the addition of a more reactive allyl iodide in the presence of HMPA gave the allylated product **4a**, albeit in a low diastereoisomeric ratio (Scheme 2). The solution came when even more reactive electrophiles such as (bromomethyl) acrylate derivatives were used. To our delight, the reaction proceeded smoothly at low temperature either *via* the *in situ* preparation of the oxenoid (**4b**, 65% yield, dr > 98 : 2, method A) or *via* the addition of oxenoid prepared in an independent flask (**4b**, 78% yield, dr > 98 : 2, method B). ^1^H, ^13^C NMR and HPLC analyses of the crude reaction mixtures were used to determine the diastereoisomeric ratios of all synthesized products. Although diastereoisomeric ratios for most of the synthesized products were similarly high, yields were consistently higher when method B was used. It should be noted that lower diastereoisomeric ratios were obtained when the allylation reaction was performed at –50 °C instead of –78 °C (dr 96 : 4 and >98 : 2 respectively). By permuting the nature of the two alkyl groups (R^1^ on the ynamide and R^2^ of the organocopper derivatives), both diastereoisomers at the quaternary carbon stereocentre are easily accessible with similar diastereoisomeric ratios using the same strategy (compare **4b** and **4c**, Scheme 2). This strategy allows the creation of two C–C bonds and one C–O bond in a single-pot operation from a simple heterosubstituted alkyne with the creation of various acyclic quaternary carbon stereocentres (see **4d–i**) in excellent diastereoisomeric ratios and yields (based on three consecutive chemical steps). Interestingly, branched alkyl groups such as cyclopropyl units and also functionalized alkyl chains could be introduced at the quaternary carbon stereocentre, expanding nicely the scope of the reaction (see **4j**, **k** and **4l–o** respectively). When benzyl-2-(bromomethyl) acrylate was used as the electrophile, similarly excellent diastereoselectivity and chemical yield were obtained (**4p**). When 3-bromo-but-3-en-2-one was used as the electrophile, we were delighted to observe that the reaction still proceeded with very high diastereomeric ratios even when the quaternary stereocentre possessed a cyclopropyl ring, albeit in slightly lower yields (**4q** and **4r** respectively). It is important to note that this reaction could be scaled up to larger quantities (1.25 g of final product) without any erosion of the diastereoisomeric ratio and chemical yield (*i.e.* formation of **4b** in 78% yield and dr > 98 : 2). The absolute configuration was determined by X-ray analysis of **4p** (see ESI†)^16^ and assigned by analogy for all products described in Scheme 2. The stereochemistry of the major isomer could be rationalized by an *anti* diastereoselective allylic alkylation reaction of the stereodefined copper enolate **3**. In this case, the oxazolidinone intramolecularly chelates the copper atom leading to a pseudo-metallacycle where one face is shielded by the benzyl group of the oxazolidinone. The functionalized allyl bromide approaches the enolate moiety *anti* to this bulky group as represented in Scheme 3. To corroborate our mechanistic hypothesis, any substituent on C_1_ of the electrophilic partner should induce steric interactions and therefore decrease the reactivity. Indeed, when (bromomethyl) acrylates bearing a methyl or a phenyl group at the terminal C_1_ position of the methylene unit were used, no allylic alkylation was observed even when the reactions were heated at higher temperature for longer periods of time.

**Scheme 4:**
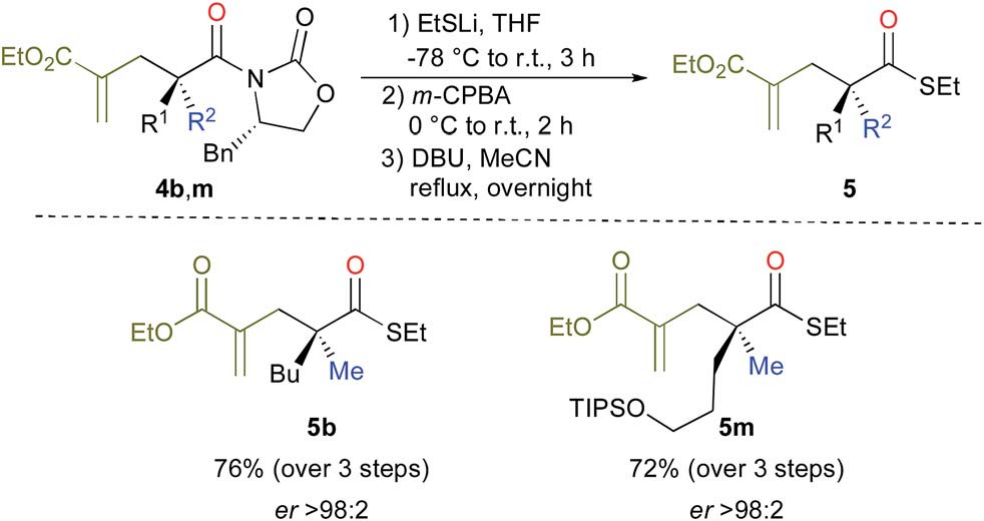
Cleavage of the oxazolidinone moiety of **4**.

Cleavage of the oxazolidinone moiety of **4** could be performed by previously described^17^ high-yielding transformations with recovery of the chiral oxazolidinone as shown in Scheme 4 (see ESI†). For instance, addition of an excess of EtSLi to **4b**,**m** in THF at –78 °C followed by oxidizing the crude reaction mixture with *m*-CPBA in DCM at 0 °C and finally heating in acetonitrile in the presence of DBU^18^ afforded the final products **5b**, **m** in good overall yields and excellent enantioselectivities as described in Scheme 4.

## Conclusions

A sequence of regio- and stereoselective carbometalation followed by oxidation of ynamides leads to stereodefined fully substituted enolates that subsequently react with various functionalized allyl bromide reagents to provide the expected products possessing an enantiomerically pure quaternary carbon stereocentre in the α-position to the carbonyl group in excellent yields and enantiomeric ratios after cleavage of the oxazolidinone moiety. It should be emphasized that three new bonds are created in a single-pot operation en route to formation of the desired acyclic quaternary stereocentre.
