# Supplementary material for: Enantioselective allylic alkylation of stereodefined polysubstituted copper enolates as an entry to acyclic quaternary carbon stereocentres
Source: Chem Sci. 2016 Sep 15;8(1):627–30. doi: 10.1039/c6sc03036j (PMC5358538; doi:10.1039/c6sc03036j)
Supplement: Supplementary file 1 [file SC-008-C6SC03036J-s001.pdf]

Electronic Supplementary Material (ESI) for Chemical Science.
This journal is © The Royal Society of Chemistry 2016

Enantioselective allylic alkylation of stereodefined polysubstituted copper enolate as an entry to acyclic quaternary carbon stereocentre†

Zackaria Nairoukh, Gunda GKS Narayana Kumar, Yury Minko and Ilan Marek*

The Mallat Family Laboratory of Organic Chemistry, Schulich Faculty of Chemistry and Lise MeitnerMinerva Center for Computational Quantum Chemistry, Technion-Israel Institute of Technology, Technion City, Haifa 32000, Israel

Table of Contents

General methods S1
Reagents and materials S2
General protocol for the preparation of starting chiral ynamides S3
General procedures for the allylation of stereodefined trisubstituted copper enolate S5
Optimization reactions S8
Products of the enantioselective allylic alkylation reaction S9
Cleavage of oxazolidinone moiety **4** S19
References S21
X-Ray crystallographic analysis data of **4p** S22
Analytical HPLC analysis data for the products of enantioselective allylic alkylation S36
NMR spectra S53

General methods

All reactions were carried out in a flame-dried glassware under positive pressure of argon in dry solvents using standard Schlenk techniques unless otherwise indicated. Progress of the reactions was monitored by analytical TLC using glass plates pre-coated with silica gel with F254 indicator (Merck). Visualization of spots was done using UV light (254 nm), iodine, *p*-anisaldehyde, phosphomolybdic acid (PMA), and Hanessian's (cerium ammonium molybdate) stains. All organometallic compounds, dry solvents and reagents were transferred using plastic single-use graduated syringes and oven-dried stainless steel needles. Purification of crude mixtures was accomplished by preparative flash column chromatography on silica gel 60A (GraceResolv), aluminum oxide 90 neutral (Merck) or Florisil® Adsorbent 100-200 mesh (Fluka) using gradient mixtures of ethyl acetate / *n*-hexane (unless otherwise indicated). ^{1}H and ^{13}C NMR spectra were measured on Bruker Avance 200 (200 MHz ^{1}H, 50 MHz ^{13}C), Bruker Avance 300 (300 MHz ^{1}H, 75 MHz ^{13}C) or Bruker Avance AV 400 (400 MHz ^{1}H, 100 MHz ^{13}C) spectrometers. Chemical shifts values (δ) are reported in ppm (calibration of spectra to the residual peak of chloroform: δ = 7.26 ppm (s) for ^{1}H NMR; δ = 77.00 ppm for ^{13}C NMR). All the proton spectra reported as following: δ value (multiplicity, J coupling constant (in Hz), number of nuclei). Multiplicity contractions used: (s) – singlet, (d) – doublet, (dd) – doublet of doublet, (t) – triplet, (q) – quartet, (m) – multiplet, and (br) – broad signal. Melting point of solid crystalline compounds was measured on Stuart® SMP11 apparatus. Optical specific rotations are measured with an UniPol L 1000 polarimeter (Na-vapor lamp, λ = 589 nm), calculated using the equation $[\alpha]_D^t = \frac{\alpha_{measured}}{l\cdot(c\cdot 10^{-3})}$, where $(l, dm; c, \frac{g}{dL})$, and reported as following: $[\alpha]_D^t$ value (*c* M, solvent). High performance liquid chromatography (HPLC) spectra were recorded on Agilent™ 1100 Series equipment with a variable wavelength UV analytical detector. Analytical separation of diastereomers from each sample was done using CHIRALPAK® AD-H 4.6 mm x 250 mmL, CHIRALCEL® OZ-H 10 mm x 250 mmL or CHIRALCEL OD 4.6 mm x 250 mm analytical HPLC column and isocratic mixtures of isopropyl alcohol and *n*-hexane (LiChrosolv® HPLC grade, Merck). MS and HRMS mass spectrometry was carried out on a APCI instrument by Maxis Impact (Bruker) and on a LCT Premier (Waters).

Reagents and materials

All solvents were purified and dried immediately prior to use: THF and diethyl ether (HPLC grade, non-stabilized, BioLab) were dried using Innovative Technology PureSolv PS-MD-2 solvent purifier (aluminum oxide columns) and kept under positive pressure of nitrogen (99.9999% purity grade); toluene was distilled from sodium metal under argon. Methyllithium solution in diethyl ether (1.60 M), *n*-butyllithium solution in hexane (1.60 M), *n*-pentyllithium solution in heptane (2.20M) and *n*-hexyllithium solution in hexane (2.30M) were purchased from Aldrich and used as received. *tert*-Butylhydroperoxide (TBHP) solution in nonane (5.5 M, over 4Å MS) was purchased from Aldrich. Terminal alkynes, ethyl acrylate, acryloyl chloride, 3-buten-2-one, 1-adamantanecarbonyl chloride, 2-methylpropanoyl chloride, N,N-diisopropylcarbamoyl chloride, *N*-bromosuccinimide, silver nitrate, iodine, (*S*)-4-benzyl-2-oxazolidinone, copper (I) bromide, copper (I) bromide – dimethylsulfide complex, copper(II) sulfate pentahydrate, 1,10-phenanthroline were purchased from commercial suppliers. (*S*)-4-isopropyl-2-oxazolidinone was prepared from commercially available starting materials and reagents according to published procedure.[1] But-3-yn-1-yl isobutyrate, pent-4-yn-1-yl adamantane-1-carboxylate and but-3-yn-1-yl diisopropylcarbamate were prepared by reaction of the corresponding commercially available acyl chlorides with commercially available 4-pentyn-1-ol and 3-butyn-1-ol in presence of a stoichiometric amount of pyridine in diethyl ether. TIPS-protected 4-pentyn-1-ol was prepared according to previously reported procedure.[2] All bromoalkynes were prepared according to a previously published procedure.[3] Purification of the corresponding compounds was accomplished using a short-path silica gel column in *n*-hexane or 10% of ethyl acetate in *n*-hexane as eluent. Cyclopropyllithium,[4] ethyl 2-(bromomethyl)acrylate,[5] benzyl 2-(bromomethyl)acrylate[6] and 3-(bromomethyl)but-3-en-2-one[7] were prepared according to a previously published procedure.

General protocol for the preparation of starting chiral ynamides

All starting chiral ynamides **1a-h** were prepared on a multi-gram scale according to a modified literature procedure.[8-9] NMR spectra of pure products **1a-e** were consistent with previously reported data.[8-9]

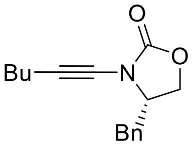

1a

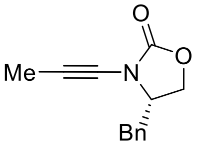

1b

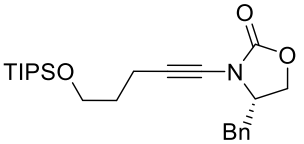

1c

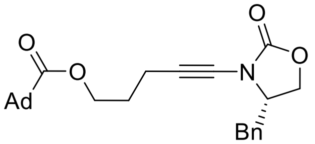

1d

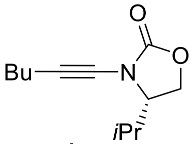

1e

(*S*)-4-(4-benzyl-2-oxooxazolidin-3-yl)but-3-yn-1-yl diisopropylcarbamate (**1f**)

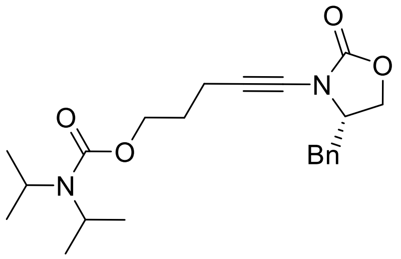

The product was obtained as a colorless oil (78%). ^{1}H NMR (400 MHz, $CDCl_3$) δ = 7.37 – 7.26 (m, 3H), 7.25 – 7.19 (m, 2H), 4.31 (t, *J* = 8.3 Hz, 1H), 4.26 – 4.16 (m, 3H), 4.10 - 3.94 (dd overlapped with bs, *J* = 8.4 Hz, 5.5, 2H), 3.79 (bs, 1H), 3.21 (dd, J^1 = 13.9 Hz, J^2 = 3.9 Hz, 1H), 2.93 (dd, J^1 = 13.9 Hz, J^2 = 8.1 Hz, 1H), 2.49 (t, *J* = 7.2 Hz, 2H), 1.97 – 1.86 (m, 2H), 1.21 (d, *J* = 6.8 Hz, 12H); ^{13}C NMR (101 MHz, $CDCl_3$) δ 155.98, 155.36, 134.27, 129.28 (*2), 128.73 (*2), 127.19, 71.83, 69.54, 67.10, 63.06, 58.05, 45.43 (*2), 37.57, 28.30, 20.95 (*4), 15.51; HRMS TOF MS ES+: $[MH]^+$ calcd. for $C_{22}H_{31}N_2O_4$ 387.2284; found 387.2234.

(*S*)-4-(4-benzyl-2-oxooxazolidin-3-yl)but-3-yn-1-yl isobutyrate (**1g**)

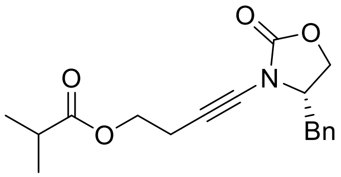

The product was obtained as a colorless oil (60%). ^{1}H NMR (400 MHz, $CDCl_3$) δ 7.38 – 7.27 (m, 3H), 7.25 – 7.18 (m, 2H), 4.32 (t, J = 8.3 Hz, 1H), 4.26 – 4.18 (m, 3H), 4.14 – 4.08 (m, 1H), 3.20 (dd, J^1 = 13.9 Hz, J^2 = 3.9 Hz, 1H), 2.93 (dd, J^1 = 13.9 Hz, J^2 = 8.1 Hz, 1H), 2.71 (t, J = 6.8 Hz, 2H), 2.58 (dt, J^1 = 14.0 Hz, J^2 = 7.0 Hz, 1H), 1.19 (d, J = 7.0 Hz, 6H); ^{13}C NMR (101 MHz, $CDCl_3$) δ 176.97, 156.09, 134.31, 129.45 (*2), 129.08 (*2), 127.58, 70.43, 69.27, 67.37, 62.20, 58.28, 37.84, 34.00, 19.23, 19.05 (*2); HRMS TOF MS ES+: $[MH]^+$ calcd. for $C_{18}H_{22}NO_4$ 316.1549; found 316.1515.

(*S*)-4-benzyl-3-(cyclopropylethynyl)oxazolidin-2-one (**1h**)

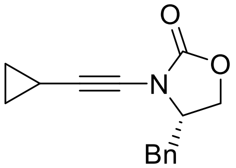

The product was obtained as a white solid (65%). mp: 46 °C; ^{1}H NMR (400 MHz, $CDCl_3$) δ 7.43 – 7.26 (m, 3H), 7.22 (d, J = 6.9 Hz, 2H), 4.30 (t, J = 8.3 Hz, 1H), 4.26 – 4.16 (m, 1H), 4.09 (dd, J^1 = 8.5 Hz, J^2 = 5.7 Hz, 1H), 3.22 (dd, J^1 = 13.9 Hz, J^2 = 3.9 Hz, 1H), 2.92 (dd, J^1 = 13.9 Hz, J^2 = 8.3 Hz, 1H), 1.41 (tt, J^1 = 8.1 Hz, J^2 = 4.9 Hz, 1H), 0.91 – 0.82 (m, 2H), 0.80 – 0.70 (m, 2H); ^{13}C NMR (101 MHz, $CDCl_3$) δ 156.34, 134.47, 129.41 (*2), 128.93 (*2), 127.39, 77.37, 67.20, 64.52, 58.30, 37.77, 8.94, 8.89, -0.73; HRMS TOF MS ES+: $[MH]^+$ calcd. for $C_{15}H_{16}NO_2$ 242.1181; found 242.1173.

General procedures for the allylation of stereodefined trisubstituted copper enolate

1- One-pot carbocupration/oxidation with *t*-BuOOH/allylation sequence (Method A)

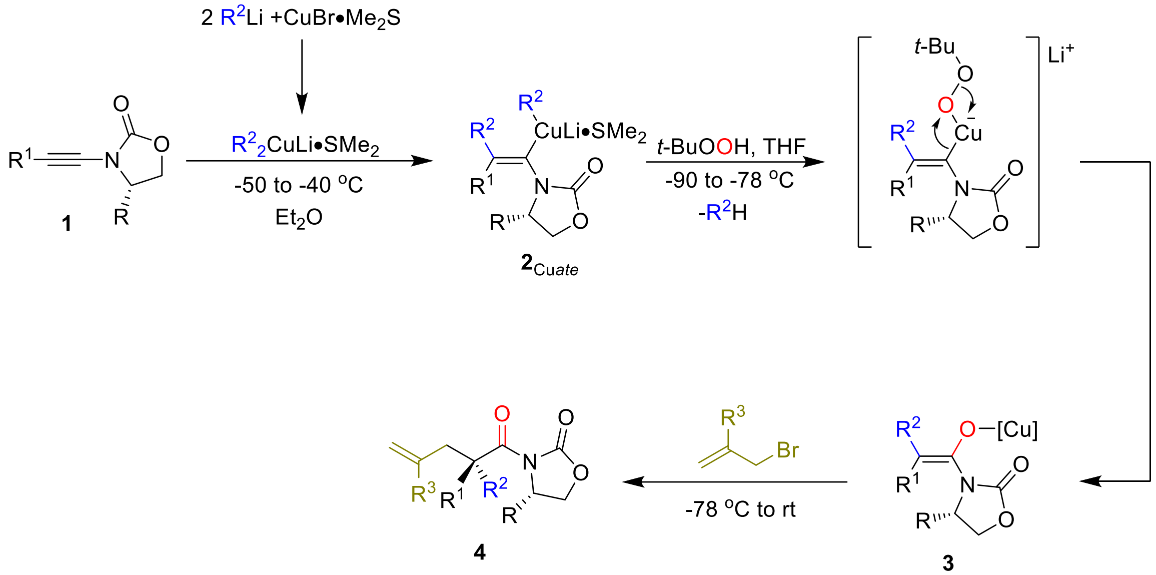

Supplementary Figure 1: One-pot sequence of carbocupration, direct oxidation with *t*-BuOOH and allylation of the stereodefined copper enolate (Method A).

CuBr•Me$_2$S complex (1.10 mmol, 1.1 equiv) was placed into a flame-dried three-necked round-bottomed flask equipped with a magnetic stirring bar and connected to an argon line. Dry diethyl ether (10 ml) was added through a septum, and the system was cooled to -50 °C using acetone-liquid nitrogen bath. A solution of alkyllithium (2.20 mmol, 2.2 equiv), was added dropwise, and the reaction mixture was allowed to warm to -40 °C and stirred for 30 min at this temperature to form a clear colourless or pale-yellow solution of dimethyl cuprate – lithium bromide complex. The reaction mixture was cooled to -50 °C and a solution of the starting alkynyl carbamate **1** (1.00 mmol, 1.0 equiv) in 3 ml of diethyl ether was added dropwise; the reaction mixture was allowed to warm to -40 °C and stirred at this temperature for 1 h (monitored by TLC on silica gel using 20% ethyl acetate in hexane as eluent) to form to form pale-yellow clear solution of vinylcuprate (Supplementary Fig. 1, first step). Upon completion of the first step, the reaction mixture was cooled to -90 °C and THF (5 ml) followed by solution of *tert*-butylhydroperoxide in nonane (1.20 mmol, 1.2 equiv) were added. The resulting reaction mixture was allowed to warm up slowly to -78 °C, and stirred at the indicated temperature for 2 h (monitored by

TLC on silica gel using 20% ethyl acetate in hexane as eluent) to form a dark-brown opaque solution (Supplementary Fig. 1, second step). Upon completion of the second step, allyl bromide derivative (3.00 mmol, 3.00 equiv.) was added neat to the reaction mixture at -78 °C and stirring was continued at the indicated temperature for 2 h (monitored by TLC on silica gel using 20% ethyl acetate in hexane as eluent). The reaction was allowed to warm to room temperature and was quenched with a 2:1 mixture of aqueous saturated solution of NH_4Cl and NH_4OH (30%). Phases were separated, and the aqueous phase was extracted three times with diethyl ether (10 ml). The combined organic phases were dried over anhydrous Na_2SO_4, filtered, and concentrated under reduced pressure. The residue was purified by column chromatography (solvent gradient 0-20% ethyl acetate in hexane unless otherwise indicated).

2- Carbocupration/oxidation with *t*-BuOOLi/allylation sequence (Method B)

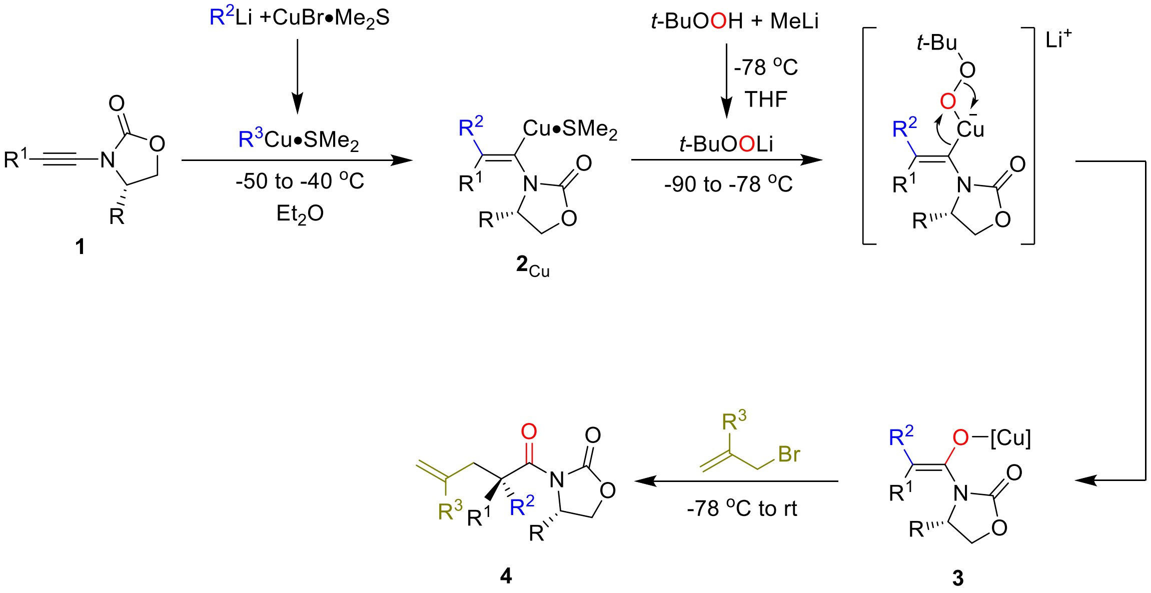

Supplementary Figure 2: One-pot sequence of carbocupration, oxidation with *t*-BuOOLi and allylation of the stereodefined copper enolate (Method B).

CuBr•Me_2S complex (1.10 mmol, 1.1 equiv) was placed into a flame-dried three-necked round-bottomed flask equipped with a magnetic stirring bar and connected to an argon line. Dry diethyl ether (10.0 ml) was added through a septum, and the system was cooled to -50 °C using acetone-liquid nitrogen bath. A solution of alkyllithium (1.30 mmol,

1.3 equiv), was added dropwise, and the reaction mixture was allowed to warm to -40 °C and stirred for 30 min at this temperature to form a bright-yellow opaque solution in case of methyllithium, dark-yellow in case of *n*-butyllithium, orange to dark-brown in case of *n*-pentyl and *n*-hexyllithium. The reaction mixture was cooled to -50 °C and a solution of the starting alkynyl carbamate **1** (1.00 mmol, 1.0 equiv) in 3 ml of diethyl ether was added dropwise; the reaction mixture was allowed to warm to -40 °C, and stirred at this temperature for 1 h (monitored by TLC on silica gel using 20% ethyl acetate in hexane as eluent) to form a solution of vinylcuprate (Supplementary Fig. 2, first step).

In a separate flask connected to an argon line, a solution of 1.54 mmol (1.54 equiv) of *t*-BuOOLi was prepared as follow. Dry THF (5.0 ml) was added to flame-dried three-necked round-bottomed flask *via* a syringe followed by addition of a solution of *tert*-butyl hydroperoxide in nonane (0.28 ml of commercially available 5.5 M solution; 1.54 equiv, 1.54 mmol). The resulting solution was cooled to –90 °C and a solution of methyllithium (1.60 M in diethyl ether, 1.80 mmol, 1.80 equiv) was added dropwise keeping the indicated temperature. The resulting solution of lithium *tert*-butylperoxide was allowed to warm to -80 °C and stir for 10 min prior to use in the following reaction step. Upon completion of the first step, the reaction mixture was cooled to -90 °C and the freshly prepared solution of lithium *tert*-butylperoxide in THF (1.54 mmol, 1.54 equiv) was transferred *via* a cannula. The resulting reaction mixture was allowed to warm up slowly to -78 °C and stirred at the indicated temperature for 2 h (monitored by TLC on silica gel using 20% ethyl acetate in hexane as eluent) to form a brown opaque solution (Supplementary Fig. 2, second step). Upon completion of the second step, allyl bromide derivative (3.00 mmol, 3.00 equiv.) was added neat to the reaction mixture at -78 °C and stirring was continued at the indicated temperature for 2 h (monitored by TLC on silica gel using 20% ethyl acetate in hexane as eluent). Upon the completion of the third step the reaction mixture was warmed to room temperature and handled as mentioned in Path A.

Optimization reactions

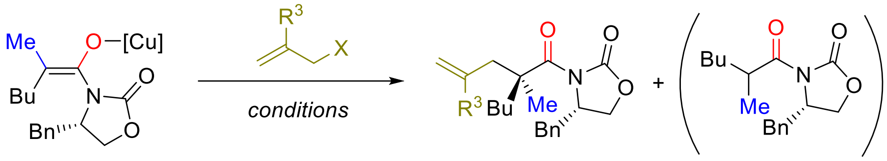

3a **4** **4'**

Entry[a]	R3-X[b]	Additive	Conditions	Conv.[c,d]	*d.r.*[c]
1	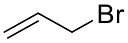	none	-78 °C to r.t. 12h	n.r.	-
2	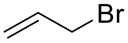	THF (5 ml)	-78 °C to r.t. 12h	n.r.	-
3	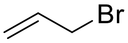	TMEDA (2 ml)	-78 °C to r.t. 12h	n.r.	-
4	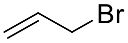	HMPA (2 ml)	-78 °C to r.t. 12h	n.r.	-
5	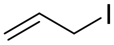	none	-78 °C to r.t. 12h	n.r.	-
6	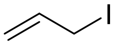	THF (5 ml)	-78 °C to r.t. 12h	n.r.	-
7	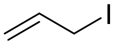	DMPU (2 ml)	-78 °C to r.t. 12h	n.r.	-
8	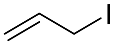	TMEDA (2 ml)	-78 °C to r.t. 12h	>20%	n.d.
9	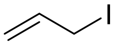	HMPA (5 equiv.)	-78 °C to r.t. 3h	>90%	50:50
10	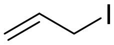	HMPA (2 ml)	-78 °C to r.t. 2h	>99%	75:25
11	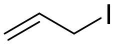	HMPA (5 ml)	-78 °C to r.t. 2h	>99%	75:25
12	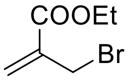	none	-50 to -20 °C 2h	>99%	96:4
13	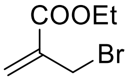	DMPU (4 equiv.)	-50 to -20 °C 2h	>99%	87:13
14	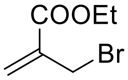	none	-78 °C to r.t. 2h	>99%	99:1

[a] General procedure 2 for the allylic alkylation was followed. [b] 3.00 equiv. of the electrophile was added. [c] Conversion and diastereomeric ratio (*d.r.*) were determined by NMR and HPLC analysis of the crude. [d] In case of no reaction (n.r.), the hydrolyzed product **4'** was isolated.

Products of the enantioselective allylic alkylation reaction

(*S*)-3-((*S*)-2-allyl-2-methylhexanoyl)-4-benzyloxazolidin-2-one (**4a**)

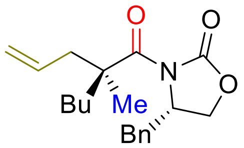

The product was obtained as a colorless oil and inseparable mixture of diastereomers (Method B: 62%, *d.r.* 75:25). Major diastereomer: ^{1}H NMR (300 MHz, $CDCl_3$) δ 7.35 – 7.16 (m, 5H), 5.85 – 5.62 (m, 1H), 5.13 – 4.95 (m, 2H), 4.71 – 4.56 (m, 1H), 4.14 – 4.03 (m, 2H), 3.27 (dd, J^1 = 13.1 Hz, J^2 = 3.1 Hz, 1H), 2.85 (dd, J^1 = 14.2 Hz, J^2 = 7.9 Hz, 1H), 2.69 – 2.56 (m, 1H), 2.41 – 2.29 (m, 1H), 2.26 – 2.14 (m, 1H), 1.65 (m, 1H), 1.40 – 1.21 (m, 6H), 1.19 – 1.06 (m, 1H), 0.87 (t, *J* = 7.0 Hz, 3H); ^{13}C NMR (75 MHz, $CDCl_3$) δ 176.88, 152.35, 135.76, 134.45, 129.45, 128.93, 127.26, 117.83, 66.18, 57.97, 49.20, 40.87, 38.06, 35.98, 26.90, 23.20, 22.73, 14.11.

Ethyl-(*S*)-4-((*S*)-4-benzyl-2-oxooxazolidine-3-carbonyl)-4-methyl-2-methyleneoctanoate (**4b**)

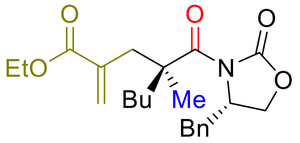

The product was obtained as a colorless oil (Method A: 65%, *d.r.* 99:1; Method B: 78%, *d.r.* 99:1). ^{1}H NMR (400 MHz, $CDCl_3$) δ 7.29 – 7.23 (m, 2H), 7.22 – 7.15 (m, 3H), 6.15 (d, *J* = 1.3 Hz, 1H), 5.51 (s, 1H), 4.57 (qd, J^1 = 6.6 Hz, J^2 = 3.1 Hz, 1H), 4.16 – 4.00 (m, 4H), 3.27 (d, *J* = 14.4 Hz, 2H), 2.75 (d, *J* = 14.0 Hz, 1H), 2.60 (dd, J^1 = 13.2 Hz, J^1 = 10.6 Hz, 1H), 2.19 – 2.03 (m, 1H), 1.47 – 1.34 (m, 1H), 1.28 – 1.14 (m, 9H), 1.05 – 0.93 (m, 1H), 0.81 (t, *J* = 7.1 Hz, 3H); ^{13}C NMR (101 MHz, $CDCl_3$) δ 176.70, 167.48, 152.63, 137.74, 136.11, 129.43 (*2), 128.90 (*2), 128.10, 127.17, 66.29, 60.75, 58.19, 49.07, 38.01, 37.88, 36.65, 27.00, 23.15, 22.35, 14.16, 14.03; HRMS TOF MS ES+: $[MH]^+$ calcd. for $C_{23}H_{32}NO_5$ 402.2280; found 402.2258; HPLC: t_R(major) = 8.632 min; t_R (minor) = 9.304 min; CHIRALPAK® AD-H (97:3 *n*-hexane – *i*-PrOH), flow rate 1 ml/min.

Ethyl (*R*)-4-((*S*)-4-benzyl-2-oxooxazolidine-3-carbonyl)-4-methyl-2-methyleneoctanoate (**4c**)

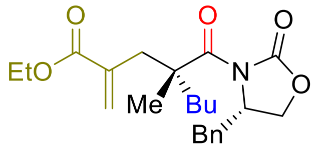

The product was obtained as a colorless oil (Method A: 60%, *d.r.* 99:1; Method B: 87%, *d.r.* 99:1). ^{1}H NMR (400 MHz, $CDCl_3$) δ 7.29 – 7.22 (m, 2H), 7.21 – 7.15 (m, 3H), 6.13 (d, J = 1.5 Hz, 1H), 5.49 (s, 1H), 4.57 (ddt, J^1 = 10.5 Hz, J^2 = 7.5 Hz, J^3 = 2.9 Hz, 1H), 4.16 (t, J^1 = 8.1 Hz, 1H), 4.08 – 4.01 (m, 3H), 3.39 (d, J = 14.1 Hz, 1H), 3.24 (dd, J^1 = 13.1 Hz, J^2 = 3.1 Hz, 1H), 2.59 (dd, J^1 = 13.1 Hz, J^2 = 10.5 Hz, 1H), 2.50 – 2.36 (m, 2H), 1.45 – 1.36 (m, 1H), 1.31 – 1.22 (m, 3H), 1.21 – 1.14 (m, 7H), 1.11 – 1.01 (m, 1H), 0.83 (t, J = 7.1 Hz, 3H); ^{13}C NMR (75 MHz, $CDCl_3$) δ 176.32, 167.26, 152.79, 137.45, 135.91, 129.31 (*2), 128.75 (*2), 128.25, 127.03, 66.10, 60.60, 57.97, 48.67, 38.21, 38.04, 36.83, 26.88, 23.06, 22.78, 14.02, 13.93; HRMS TOF MS ES+: [MH]$^+$ calcd. for $C_{23}H_{32}NO_5$ 402.2280; found 402.2251; HPLC: t_R(major) = 9.284 min; t_R (minor) = 8.640 min; CHIRALPAK® AD-H (97:3 *n*-hexane – *i*-PrOH), flow rate 1 ml/min.

Ethyl (*S*)-4-((*S*)-4-benzyl-2-oxooxazolidine-3-carbonyl)-4-ethyl-2-methyleneoctanoate (**4d**)

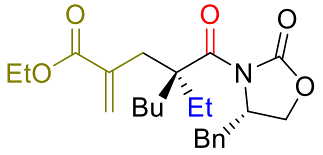

The product was obtained as a colorless oil (Method A: 61%, *d.r.* 97:3). ^{1}H NMR (400 MHz, $CDCl_3$) δ 7.29 – 7.22 (m, 2H), 7.22 – 7.15 (m, 3H), 6.13 (s, 1H), 5.48 (s, 1H), 4.58 (qd, J^1 = 6.4 Hz, J^2 = 2.9 Hz, 1H), 4.14 – 3.99 (m, 4H), 3.28 (dd, J^1 = 13.1 Hz, J^2 = 3.0 Hz, 1H), 3.09 (d, J =14.5 Hz, 1H), 2.86 (d, J = 14.5 Hz, 1H), 2.57 (dd, J^1 = 13.0 Hz, J^2 =10.8 Hz, 1H), 2.08 (dq, J^1 = 14.8 Hz, J^2 = 7.4 Hz, 1H), 1.88 – 1.76 (m, 1H), 1.75 – 1.67 (m, 1H), 1.67 – 1.58 (m, 1H), 1.26 – 1.14 (m, 6H), 1.11 – 1.04 (m, 1H), 0.83 (t, J = 7.2 Hz, 3H), 0.78 (t, J = 7.4 Hz, 3H); ^{13}C NMR (101 MHz, $CDCl_3$) δ 176.83, 167.51, 152.80, 137.94, 136.17, 129.40 (*2), 128.89 (*2), 127.70, 127.14, 66.23, 60.74, 58.38, 52.22, 38.11, 34.09, 31.37, 26.05, 24.72, 23.16, 14.14, 14.10, 8.62; HRMS TOF MS ES+: [M+Na]$^+$ calcd. for

$C_{24}H_{33}NO_5Na$ 438.2256; found 438.2259; HPLC: t_R(major) = 6.151 min; t_R (minor) = 7.116 min; CHIRALPAK® AD-H (97:3 *n*-hexane – *i*-PrOH), flow rate 1 ml/min.

Ethyl (*R*)-4-((*S*)-4-benzyl-2-oxooxazolidine-3-carbonyl)-4-butyl-2-methylenenonanoate (**4e**)

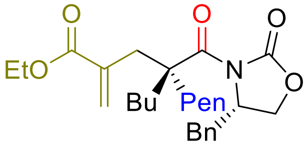

The product was obtained as a colorless oil (Method B: 76%, *d.r.* 94:6). ^{1}H NMR (400 MHz, $CDCl_3$) δ 7.28 – 7.21 (m, 2H), 7.21 – 7.14 (m, 3H), 6.12 (d, *J* = 1.5 Hz, 1H), 5.46 (d, *J* = 0.9 Hz, 1H), 4.58 (ddt, J^1 = 7.3 Hz, J^2 = 6.1 Hz, J^3 = 3.0 Hz, 1H), 4.15 – 3.98 (m, 4H), 3.27 (dd, J^1 = 13.1 Hz, J^2 = 3.2 Hz, 1H), 3.11 (d, *J* = 14.5 Hz, 1H), 2.83 (d, *J* = 14.5 Hz, 1H), 2.57 (dd, J^1 = 13.1 Hz, J^2 = 10.7 Hz, 1H), 2.08 – 1.97 (m, 1H), 1.85 – 1.74 (m, 1H), 1.70 – 1.57 (m, 2H), 1.27 – 1.06 (m, 13H), 0.86 – 0.78 (m, 6H); ^{13}C NMR (101 MHz, $CDCl_3$) δ 176.90, 167.49, 152.78, 137.95, 136.16, 129.41 (*2), 128.88 (*2), 127.67, 127.13, 66.20, 60.73, 58.35, 51.88, 38.08, 34.48, 32.29, 32.05, 31.94, 26.06, 23.83, 23.15, 22.57, 14.14, 14.11, 14.08; HRMS TOF MS ES+: $[M+Na]^+$ calcd. for $C_{27}H_{39}NO_5Na$ 480.2726; found 480.2720; HPLC: t_R(major) = 8.004 min; t_R (minor) = 7.615 min; CHIRALPAK® AD-H (98:2 *n*-hexane – *i*-PrOH), flow rate 1 ml/min.

Ethyl (*R*)-4-((*S*)-4-benzyl-2-oxooxazolidine-3-carbonyl)-4-butyl-2-methylenedecanoate (**4f**)

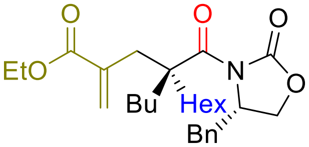

The product was obtained as a colorless oil (Method B: 76%, *d.r.* 92:8). ^{1}H NMR (400 MHz, $CDCl_3$) δ 7.29 – 7.23 (m, 2H), 7.20 – 7.16 (m, 3H), 6.12 (d, *J* = 1.4 Hz, 1H), 5.46 (s, 1H), 4.58 (tt, J^1 = 7.1 Hz, J^2 = 2.9 Hz, 1H), 4.16 – 3.97 (m, 4H), 3.27 (dd, J^1 = 13.1 Hz, J^2 = 3.0 Hz, 1H), 3.11 (d, *J* = 14.5 Hz, 1H), 2.83 (d, *J* = 14.5 Hz, 1H), 2.57 (dd, J^1 = 13.0 Hz, J^2 = 10.8 Hz, 1H), 2.10 – 1.98 (m, 1H), 1.87 – 1.74 (m, 1H), 1.69 – 1.58 (m, 2H), 1.27 – 1.05 (m, 15H), 0.87 – 0.77 (m, 6H); ^{13}C NMR (101 MHz, $CDCl_3$) δ 176.93, 167.51, 152.78,

137.95, 136.17, 129.42 (*2), 128.89 (*2), 127.68, 127.14, 66.21, 60.75, 58.37, 51.90, 38.08, 34.47, 32.12, 31.94, 31.74, 29.78, 26.08, 24.12, 23.16, 22.67, 14.15, 14.12, 14.07; HRMS TOF MS ES+: $[MH]^+$ calcd. for $C_{28}H_{42}NO_5$ 472.3062; found 472.3063; HPLC: t_R(major) = 6.502 min; t_R (minor) = 5.809 min; CHIRALPAK® AD-H (97:3 *n*-hexane – *i*-PrOH), flow rate 1 ml/min.

Ethyl (*S*)-4-((*S*)-4-benzyl-2-oxooxazolidine-3-carbonyl)-4-butyl-2-methylenedecanoate (**4g**)

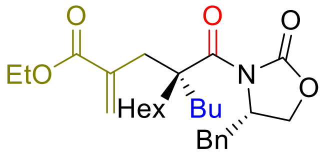

The product was obtained as a colorless oil (Method B: 79%, *d.r.* 94:6). ^{1}H NMR (300 MHz, $CDCl_3$) δ 7.35 – 7.27 (m, 2H), 7.27 – 7.14 (m, 3H), 6.17 (s, 1H), 5.51 (s, 1H), 4.62 (ddd, J^1 =10.2 Hz, J^2 = 6.2 Hz, J^3 = 2.9 Hz, 1H), 4.23 – 4.04 (m, 4H), 3.32 (dd, J^1 = 13.1 Hz, J^2 = 3.2 Hz, 1H), 3.14 (d, J = 14.5 Hz, 1H), 2.90 (d, J = 14.5 Hz, 1H), 2.66 – 2.56 (m, 1H), 2.16 – 2.00 (m, 1H), 1.91 – 1.79 (m, 1H), 1.75 – 1.60 (m, 2H), 1.40 – 1.01 (m, 15H), 0.91 – 0.82 (m, 6H); ^{13}C NMR (75 MHz, $CDCl_3$) δ 176.81, 167.36, 152.65, 137.76, 136.03, 129.29 (*2), 128.76 (*2), 127.62, 127.01, 66.08, 60.63, 58.26, 51.74, 37.92, 34.31, 32.05, 31.64, 29.64, 26.21, 23.68, 23.03, 22.56, 14.03, 13.98, 13.95; HRMS TOF MS ES+: $[MH]^+$ calcd. for $C_{28}H_{42}NO_5$ 472.3062; found 472.3072; HPLC: t_R(major) = 5.830 min; t_R (minor) = 6.542 min; CHIRALPAK® AD-H (97:3 *n*-hexane – *i*-PrOH), flow rate 1 ml/min.

Ethyl (*S*)-4-((*S*)-4-benzyl-2-oxooxazolidine-3-carbonyl)-4-methyl-2-methylenedecanoate (**4h**)

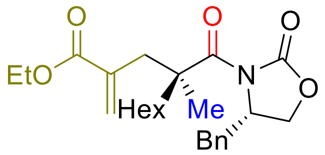

The product was obtained as a colorless oil (Method B: 81%, *d.r.* 98:2). ^{1}H NMR (400 MHz, $CDCl_3$) δ 7.29 – 7.22 (m, 2H), 7.22 – 7.15 (m, 3H), 6.15 (d, J = 1.4 Hz, 1H), 5.50 (s, 1H), 4.57 (qd, J^1 = 6.6 Hz, J^2 = 3.2 Hz, 1H), 4.16 – 3.99 (m, 4H), 3.27 (d, J = 13.8 Hz, 2H), 2.74 (d, J = 14.0 Hz, 1H), 2.60 (dd, J^1 = 13.2 Hz, J^1 = 10.6 Hz, 1H), 2.20 – 2.03 (m, 1H),

1.44 – 1.35 (m, 1H), 1.30 – 1.15 (m, 13H), 1.07 – 0.95 (m, 1H), 0.79 (t, *J* = 6.6 Hz, 3H); ^{13}C NMR (101 MHz, $CDCl_3$) δ 176.79, 167.57, 152.72, 137.85, 136.22, 129.53 (*2), 129.00 (*2), 128.18, 127.26, 66.38, 60.84, 58.29, 49.21, 38.13, 37.97, 37.04, 31.78, 29.85, 24.83, 22.70, 22.45, 14.26, 14.14; HRMS TOF MS ES+: $[MH]^+$ calcd. for $C_{25}H_{36}NO_5$ 430.2593; found 430.2593; HPLC: t_R(major) = 7.610 min; t_R (minor) = 8.433 min; CHIRALPAK® AD-H (97:3 *n*-hexane – *i*-PrOH), flow rate 1 ml/min.

Ethyl (*R*)-4-((*S*)-4-benzyl-2-oxooxazolidine-3-carbonyl)-4-methyl-2-methylenedecanoate (**4i**)

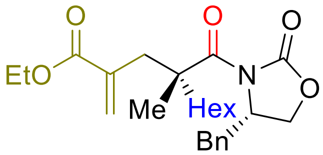

The product was obtained as a colorless oil (Method B: 83%, *d.r.* 99:1). ^{1}H NMR (300 MHz, $CDCl_3$) δ 7.33 – 7.27 (m, 2H), 7.26 – 7.20 (m, 3H), 6.18 (s, 1H), 5.53 (s, 1H), 4.61 (qd, J^1 = 6.9 Hz, J^2 = 3.2 Hz, 1H), 4.20 (t, *J* = 8.1 Hz, 1H), 4.13 – 4.05 (m, 3H), 3.44 (d, *J* = 14.1 Hz, 1H), 3.28 (dd, J^1 = 13.1 Hz, J^2 = 3.5 Hz, 1H), 2.63 (dd, J^1 = 13.3 Hz, J^2 = 10.3 Hz, 1H), 2.56 – 2.35 (m, 2H), 1.50 – 1.37 (m, 1H), 1.36 – 1.15 (m, 13H), 1.15 – 1.00 (m, 1H), 0.85 (t, *J* = 6.6 Hz, 3H); ^{13}C NMR (75 MHz, $CDCl_3$) δ 176.31, 167.25, 152.79, 137.45, 135.92, 129.31 (*2), 128.75 (*2), 128.24, 127.03, 66.10, 60.59, 57.96, 48.70, 38.23, 38.03, 37.12, 31.60, 29.66, 24.65, 22.79, 22.50, 14.02, 13.96; HRMS TOF MS ES+: $[MH]^+$ calcd. for $C_{25}H_{36}NO_5$ 430.2593; found 430.2560; HPLC: t_R(major) = 8.483 min; t_R (minor) = 7.669 min; CHIRALPAK® AD-H (97:3 *n*-hexane – *i*-PrOH), flow rate 1 ml/min.

Ethyl (*R*)-5-((*S*)-4-benzyl-2-oxooxazolidin-3-yl)-4-cyclopropyl-4-methyl-2-methylene-5-oxopentanoate (**4j**)

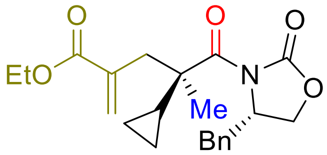

The product was obtained as a colorless oil (Method B: 74%, *d.r.* 99:1). ^{1}H NMR (400 MHz, $CDCl_3$) δ 7.28 – 7.22 (m, 2H), 7.21 – 7.14 (m, *J* = 6.8 Hz, 3H), 6.15 (s, 1H), 5.55 (s, 1H), 4.57 (td, J^1 = 7.4 Hz, J^2 = 3.6 Hz, 1H), 4.17 – 4.00 (m, 4H), 3.38 (d, *J* = 13.8 Hz, 1H),

3.27 (dd, J^1 = 13.2 Hz, J^2 = 2.8 Hz, 1H), 2.93 (d, J = 13.8 Hz, 1H), 2.66 (dd, J^1 = 13.1 Hz, J^2 = 10.6 Hz, 1H), 1.72 – 1.64 (m, 1H), 1.19 (t, J = 7.1 Hz, 3H), 0.82 (s, 3H), 0.38 – 0.28 (m, 3H), 0.17 – 0.09 (m, 1H); ^{13}C NMR (101 MHz, $CDCl_3$) δ 177.10, 167.45, 152.88, 137.72, 136.28, 129.57 (*2), 128.97 (*2), 128.27, 127.23, 66.36, 60.81, 58.47, 48.14, 38.66, 37.88, 17.32, 15.86, 14.27, 1.90, 1.21; HRMS TOF MS ES+: $[MH]^+$ calcd. for $C_{22}H_{28}NO_5$ 386.1967; found 386.1967; HPLC: t_R(major) = 12.974 min; CHIRALPAK® AD-H (97:3 *n*-hexane – *i*-PrOH), flow rate 1 ml/min.

Ethyl (*S*)-5-((*S*)-4-benzyl-2-oxooxazolidin-3-yl)-4-cyclopropyl-4-methyl-2-methylene-5-oxopentanoate (**4k**)

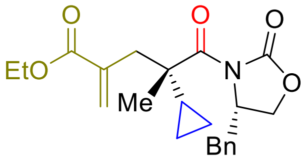

The product was obtained as a colorless oil (Method B: 70%, *d.r.* 97:3). ^{1}H NMR (400 MHz, $CDCl_3$) δ 7.12 – 7.07 (m, 2H), 7.06 – 7.00 (m, 3H), 5.99 (dd, J^1 = 7.6 Hz, J^2 = 1.6 Hz, 1H), 5.36 (s, 1H), 4.47 (ddt, J^1 = 10.5 Hz, J^2 = 7.6 Hz, J^3 = 3.0 Hz, 1H), 4.03 (t, J = 8.2 Hz, 1H), 3.95 – 3.85 (m, 3H), 3.41 (d, J = 14.1 Hz, 1H), 3.02 (dd, J^1 = 13.3 Hz, J^2 = 3.2 Hz, 1H), 2.56 – 2.43 (m, 2H), 1.94 – 1.83 (m, 1H), 1.03 (t, J = 7.1 Hz, 3H), 0.59 (s, 3H), 0.25 – 0.15 (m, 3H), 0.05 – -0.04 (m, 1H); ^{13}C NMR (101 MHz, $CDCl_3$) δ 176.71, 167.38, 153.09, 137.69, 136.04, 129.57 (*2), 128.93 (*2), 128.38, 127.24, 66.33, 60.78, 57.97, 47.78, 38.83, 38.24, 17.50, 15.60, 14.24, 1.76, 1.03; HRMS TOF MS ES+: $[MH]^+$ calcd. for $C_{22}H_{27}NO_5Na$ 408.1787; found 408.1736; HPLC: t_R(major) = 16.420 min; t_R (minor) = 13.241 min; CHIRALPAK® AD-H (97:3 *n*-hexane – *i*-PrOH), flow rate 1 ml/min.

Ethyl (*R*)-4-((*S*)-4-benzyl-2-oxooxazolidine-3-carbonyl)-6-(isobutyryloxy)-4-methyl-2-methylenehexanoate (**4l**)

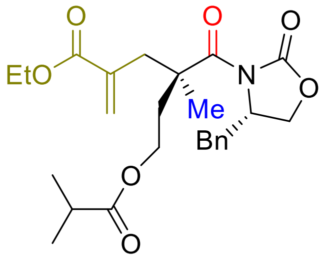

The product was obtained as a colorless oil (Method B: 60%, *d.r.* 95:5). ^{1}H NMR (400 MHz, $CDCl_3$) δ 7.28 – 7.23 (m, 2H), 7.22 – 7.15 (m, 3H), 6.19 (d, *J* = 1.4 Hz, 1H), 5.54 (s, 1H), 4.59 (ddt, J^1 = 7.3 Hz, J^2 = 6.2 Hz, J^3 = 3.0 Hz, 1H), 4.18 (t, *J* = 8.2 Hz, 1H), 4.13 – 3.98 (m, 5H), 3.25 (dd, J^1 = 13.2 Hz, J^2 = 3.2 Hz, 1H), 3.17 (d, *J* = 14.0 Hz, 1H), 2.92 (d, *J* = 13.9 Hz, 1H), 2.66 – 2.51 (m, 2H), 2.45 – 2.34 (m, 1H), 1.72 (dt, J^1 = 14.2 Hz, J^2 = 7.0 Hz, 1H), 1.29 (s, 3H), 1.22 – 1.18 (t, *J* = 7.11 Hz, 3H), 1.07 (dd, J^1 = 7.0 Hz, J^2 = 0.4 Hz, 6H); ^{13}C NMR (101 MHz, $CDCl_3$) δ 191.30, 177.11, 175.87, 167.36, 152.92, 137.18, 136.08, 129.52 (*2), 129.03 (*2), 128.91, 127.32, 66.55, 61.33, 60.96, 58.25, 47.77, 38.30, 38.08, 35.86, 34.07, 22.38, 19.05, 19.04, 14.25; HRMS TOF MS ES+: $[MH]^+$ calcd. for $C_{25}H_{34}NO_5$ 460.2335; found 460.2384; HPLC: t_R(major) = 10.634 min; t_R (minor) = 9.775 min; CHIRALPAK® AD-H (90:10 *n*-hexane – *i*-PrOH), flow rate 1 ml/min.

Ethyl (*S*)-4-((*S*)-4-benzyl-2-oxooxazolidine-3-carbonyl)-4-methyl-2-methylene-7-((triisopropylsilyl)oxy)heptanoate (**4m**)

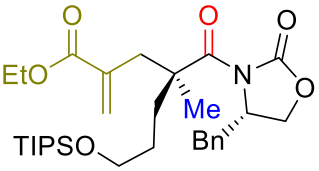

The product was obtained as a colorless oil (Method B: 85%, *d.r.* 99:1). ^{1}H NMR (400 MHz, $CDCl_3$) δ 7.28 – 7.23 (m, 2H), 7.22 – 7.15 (m, 3H), 6.16 (d, *J* = 1.5 Hz, 1H), 5.52 (s, 1H), 4.57 (qd, J^1 = 6.7 Hz, J^2 = 3.2 Hz, 1H), 4.14 – 3.99 (m, 4H), 3.63 – 3.50 (m, 2H), 3.30 – 3.20 (m, 2H), 2.81 (d, *J* = 14.0 Hz, 1H), 2.60 (dd, J^1 = 13.2 Hz, J^2 = 10.5 Hz, 1H), 2.21 – 2.10 (m, 1H), 1.62 – 1.38 (m, 3H), 1.37 – 1.27 (m, 1H), 1.25 (s, 3H), 1.20 (m, 4H), 1.04 – 1.00 (m, 1H), 0.99 – 0.94 (m, 18H); ^{13}C NMR (101 MHz, $CDCl_3$) δ 176.65, 167.55, 152.72, 137.73, 136.23, 129.55 (*2), 129.02 (*2), 128.35, 127.29, 66.40, 63.68, 60.87,

58.25, 48.90, 38.19, 38.04, 33.43, 28.64, 22.48, 18.15 (*6), 14.28, 12.11 (*3); HRMS TOF MS ES+: $[MH]^+$ calcd. for $C_{31}H_{50}NO_6Si$ 560.3407; found 560.3420; HPLC: t_R(major) = 12.913 min; t_R (minor) = 12.371 min; CHIRALCEL® OZ-H (99:1 *n*-hexane – *i*-PrOH), flow rate 0.7 ml/min.

(*S*)-4-((*S*)-4-benzyl-2-oxooxazolidine-3-carbonyl)-6-(ethoxycarbonyl)-4-methylhept-6-en-1-yl (*3R*)-adamantane-1-carboxylate (**4n**)

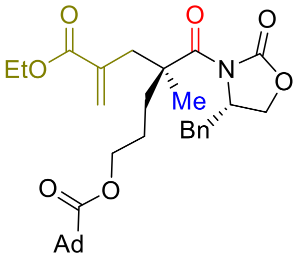

The product was obtained as a colorless oil (Method B: 71%, *d.r.* 99:1). ^{1}H NMR (400 MHz, $CDCl_3$) δ 7.29 – 7.23 (m, 2H), 7.22 – 7.15 (m, 3H), 6.17 (s, 1H), 5.52 (s, 1H), 4.58 (td, J^1 = 7.4 Hz, J^2 = 3.7 Hz, 1H), 4.17 – 4.02 (m, 4H), 3.94 (t, J = 5.9 Hz, 2H), 3.30 – 3.17 (m, 2H), 2.84 (d, J = 14.0 Hz, 1H), 2.61 (dd, J^1 = 13.0 Hz, J^2 = 10.6 Hz, 1H), 2.19 (t, J = 10.9 Hz, 1H), 1.98 – 1.87 (m, 3H), 1.84 – 1.75 (m, 6H), 1.67 – 1.58 (m, 6H), 1.50 – 1.38 (m, 3H), 1.26 (s, 3H), 1.20 (t, J = 7.1 Hz, 3H); ^{13}C NMR (101 MHz, $CDCl_3$) δ 177.77, 176.28, 167.40, 152.69, 137.55, 136.11, 129.49 (*2), 128.97 (*2), 128.40, 127.26, 66.42, 64.19, 60.85, 58.17, 48.71, 40.78, 38.89 (*3), 38.07, 37.99, 36.59 (*3), 33.50, 28.04 (*3), 24.33, 22.27, 14.23; HRMS TOF MS ES+: $[MH]^+$ calcd. for $C_{33}H_{44}NO_7$ 566.3118; found 566.3132; HPLC: t_R(major) = 15.557 min; t_R (minor) = 13.951 min; CHIRALPAK® AD-H (97:3 *n*-hexane – *i*-PrOH), flow rate 1 ml/min.

Ethyl (*R*)-4-((*S*)-4-benzyl-2-oxooxazolidine-3-carbonyl)-6-((diisopropylcarbamoyl)oxy)-4-methyl-2-methylenehexanoate (**4o**)

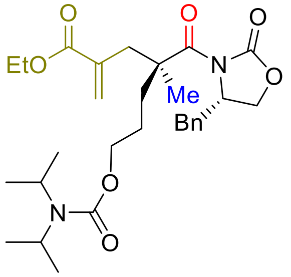

The product was obtained as a colorless oil (Method A: 52%, *d.r.* 98:2). ^{1}H NMR (400 MHz, $CDCl_3$) δ 7.29 – 7.22 (m, 2H), 7.21 – 7.14 (m, 3H), 6.16 (d, *J* = 1.4 Hz, 1H), 5.52 (s, 1H), 4.58 (qd, J^1 = 6.3 Hz, J^2 = 3.0 Hz, 1H), 4.18 – 3.89 (m, 7H), 3.72 (bs, 1H), 3.30 – 3.16 (m, 2H), 2.85 (d, *J* = 14.0 Hz, 1H), 2.61 (dd, J^1 = 13.2 Hz, J^2 = 10.5 Hz, 1H), 2.23 (t, *J* = 10.7 Hz, 1H), 1.64 – 1.56 (m, 1H), 1.52 – 1.37 (m, 2H), 1.26 (s, 3H), 1.20 (t, *J* = 7.1 Hz, 3H), 1.13 (dd, J^1 = 6.8 Hz, J^2 = 1.8 Hz, 12H); ^{13}C NMR (101 MHz, $CDCl_3$) δ 176.34, 167.40, 155.70, 152.69, 137.53, 136.11, 129.49 (*2), 128.96 (*2), 128.40, 127.23, 66.41, 64.78, 60.83, 58.15, 48.72, 46.12 (*2), 38.13, 37.96, 33.89, 24.70, 22.31, 20.88 (*4), 14.23; HRMS TOF MS ES+: $[MH]^+$ calcd. for $C_{29}H_{42}N_2O_7$ 531.3070; found 531.3070; HPLC: t_R(major) = 18.730 min; t_R (minor) = 16.692 min; CHIRALPAK® AD-H (97:3 *n*-hexane – *i*-PrOH), flow rate 1 ml/min.

Benzyl (*S*)-4-((*S*)-4-benzyl-2-oxooxazolidine-3-carbonyl)-4-methyl-2-methyleneoctanoate (**4p**)

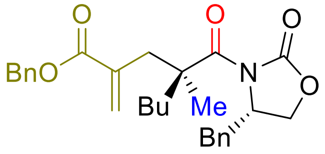

The product was obtained as a white solid (Method B: 84%, *d.r.* 99:1). mp: 56 °C; ^{1}H NMR (400 MHz, $CDCl_3$) δ 7.31 – 7.08 (m, 10H), 6.22 (d, *J* = 1.4 Hz, 1H), 5.55 (s, 1H), 5.11 – 5.01 (m, 2H), 4.55 – 4.42 (m, 1H), 4.07 – 3.95 (m, 2H), 3.30 – 3.19 (m, 2H), 2.79 (d, *J* = 14.0 Hz, 1H), 2.57 (dd, J^1 = 13.1 Hz, J^2 = 10.6 Hz, 1H), 2.14 – 2.03 (m, 1H), 1.47 – 1.34 (m, 1H), 1.28 – 1.14 (m, 6H), 1.07 – 0.92 (m, 1H), 0.80 (t, *J* = 7.1 Hz, 3H);^{13}C NMR (101 MHz, $CDCl_3$) δ 176.70, 167.33, 152.75, 137.51, 136.19, 136.12, 129.53 (*2), 128.96 (*2), 128.88, 128.61 (*2), 128.22, 128.08 (*2), 127.24, 66.61, 66.38, 58.20, 49.06, 38.17, 37.96, 36.85, 27.06, 23.24, 22.41, 14.14; HRMS TOF MS ES+: $[MH]^+$ calcd. for $C_{28}H_{34}NO_5$ 464.2437; found 464.2490; HPLC: t_R(major) = 10.547 min; t_R (minor) = 11.442 min; CHIRALPAK® AD-H (95:5 *n*-hexane – *i*-PrOH), flow rate 1 ml/min. Single crystals of the title compound **4p** suitable for X-ray diffraction analysis were obtained by crystallization at room temperature from (1:1) mixture of *n*-hexane and ethyl acetate (slow evaporation of solvents). Detailed crystallographic data reported in the corresponding section of the Supplementary Information.

(*S*)-1-((*S*)-4-benzyl-2-oxooxazolidin-3-yl)-2-butyl-2-methyl-4-methylenehexane-1,5-dione (**4q**)

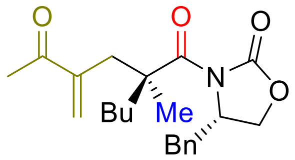

The product was obtained as a white solid (Method B: 67%, *d.r.* 99:1). mp: 63 °C; ^{1}H NMR (400 MHz, $CDCl_3$) δ 7.28 – 7.22 (m, 2H), 7.21 – 7.13 (m, 3H), 6.01 (s, 1H), 5.73 (s, 1H), 4.56 (ddd, J^1 = 10.3 Hz, J^2 = 7.0 Hz, J^3 = 3.3 Hz, 1H), 4.15 – 4.01 (m, 2H), 3.31 – 3.19 (m, 2H), 2.74 – 2.58 (m, 2H), 2.22 (s, 3H), 2.14 – 2.04 (m, 1H), 1.36 – 1.19 (m, 4H), 1.16 (s, 3H), 1.04 – 0.92 (m, 1H), 0.80 (t, *J* = 7.1 Hz, 3H); ^{13}C NMR (101 MHz, $CDCl_3$) δ 199.86, 176.81, 152.77, 146.34, 136.29, 129.53 (*2), 128.93 (*2), 128.46, 127.18, 66.36, 58.21, 48.71, 37.97, 37.35, 37.17, 27.08, 25.91, 23.25, 22.64, 14.11; HRMS TOF MS ES+: $[MH]^+$ calcd. for $C_{22}H_{29}NO_4$ 372.2175; found 372.2160; HPLC: t_R(major) = 7.970 min; t_R (minor) = 7.437 min; CHIRALCEL OD (90:10 *n*-hexane – *i*-PrOH), flow rate 1 ml/min.

(*R*)-1-((*S*)-4-benzyl-2-oxooxazolidin-3-yl)-2-cyclopropyl-2-methyl-4-methylenehexane-1,5-dione (**4r**)

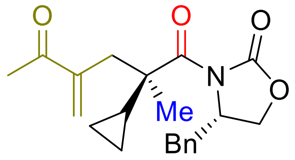

The product was obtained as a colorless oil (Method B: 60%, *d.r.* 99:1). ^{1}H NMR (400 MHz, $CDCl_3$) δ 7.15 – 7.09 (m, 2H), 7.07 – 7.01 (m, 3H), 5.89 (s, 1H), 5.65 (s, 1H), 4.50 – 4.39 (m, 1H), 4.02 (t, *J* = 8.0 Hz, 1H), 3.93 (dd, J^1 = 8.8 Hz, J^2 = 2.3 Hz, 1H), 3.21 (d, J^1 = 13.6 Hz, 1H), 3.14 (dd, J^1 = 13.3 Hz, J^2 = 3.0 Hz, 1H), 2.73 (d, *J* = 13.6 Hz, 1H), 2.56 (dd, J^1 = 13.2 Hz, J^2 = 10.6 Hz, 1H), 2.09 (s, 3H), 1.50 (dq, J^1 = 7.8 Hz, J^2 = 6.2 Hz, 1H), 0.63 (s, 3H), 0.27 – 0.11 (m, 3H), 0.04 – -0.07 (m, 1H); ^{13}C NMR (101 MHz, $CDCl_3$) δ 198.49, 175.83, 151.59, 144.83, 135.02, 128.24 (*2), 127.58 (*2), 127.41, 125.82, 65.00, 57.10, 46.38, 36.60, 36.53, 24.56, 16.07, 14.75, 0.43, 0.00; HRMS TOF MS ES+: $[MH]^+$ calcd. for $C_{21}H_{25}NO_4$ 356.1862; found 356.1836; HPLC: t_R(major) = 9.327 min; t_R (minor) = 9.868 min; CHIRALPAK® AD-H (90:10 *n*-hexane – *i*-PrOH), flow rate 1 ml/min.

Cleavage of oxazolidinone moiety 4

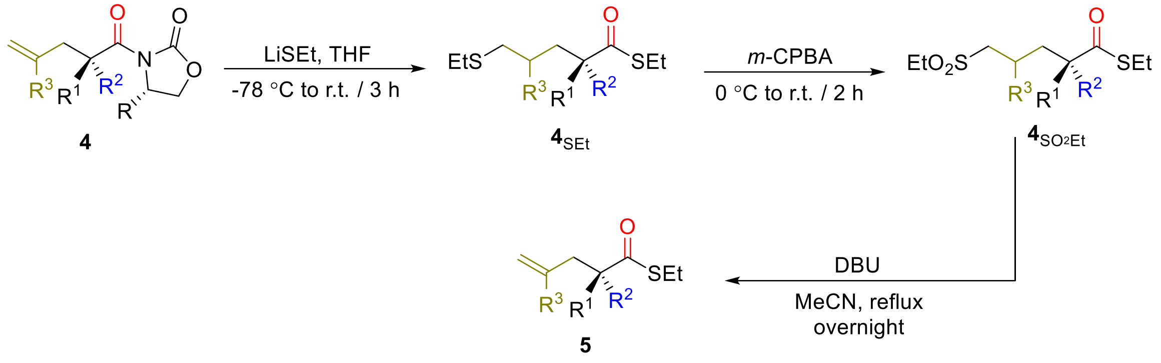

Supplementary Figure 3: Removal of the chiral auxiliary, oxidation and elimination sequence

5 was prepared according to a modified literature procedure.[10] To a suspension of ethanethiol (7.00 equiv.) in THF (0.4 M) at −78 °C was slowly added *n*-BuLi (6.50 equiv., 1.6 M in hexane). After 30 min, a solution of **4** (1.00 equiv.) in THF (5 mL) was slowly added to the reaction mixture at −78 °C, and the contents were allowed to gradually warm up to room temperature over 3 h. Upon completion of the first step (monitored by TLC on silica gel using 20% ethyl acetate in hexane as eluent), the reaction mixture was quenched with sat. $NaHCO_3$ (15 mL) and extracted with Et_2O (3 × 20 mL). The combined organic phases were dried over anhydrous Na_2SO_4, filtered, and concentrated under reduced pressure affording thioester $\mathbf{4}_{SEt}$.

The thioester crude $\mathbf{4}_{SEt}$ was then dissolved in DCM (0.1 M) and cooled to 0 °C followed by the addition of *m*-CPBA (5.00 equiv.). The resulting mixture was slowly warm up to room temperature over 2 h. Upon completion of the second step (monitored by TLC on silica gel using 20% ethyl acetate in hexane as eluent), a solution of 10% Na_2SO_4 was added and the mixture was stirred at room temperature for 20 min. Phases were separated, and the aqueous phase was extracted three times with chloroform (10 ml). The combined organic phases were dried over anhydrous $MgSO_4$, filtered, and concentrated under reduced pressure affording $\mathbf{4}_{SO_2Et}$.

Then, $\mathbf{4}_{SO_2Et}$ was dissolved in acetonitrile (0.1 M) followed by the addition of 1,8-diazabicycloundec-7-ene (1.10 equiv., DBU). After refluxing for 6 h, the reaction mixture was cooled to room temperature and a solution of 5% HCl was added (20 ml). Phases were

separated and the aqueous phase was extracted three times with chloroform (10 ml). The combined organic phases were dried over anhydrous Na_2SO_4, filtered, and concentrated under reduced pressure. The residue was purified by column chromatography (solvent gradient 0-10% ethyl acetate in hexane).

Ethyl (*S*)-4-((ethylthio)carbonyl)-4-methyl-2-methyleneoctanoate (**5b**)

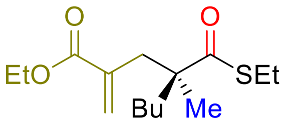

The product was obtained as a colorless oil (76% over 3 steps, *e.r.* 99:1). $[\alpha]_D^{20}$ = - 0.61 (*c* = 0.56, CH_2Cl_2); ^1H NMR (300 MHz, $CDCl_3$) δ 6.18 (d, *J* = 1.5 Hz, 1H), 5.49 (d, *J* = 0.7 Hz, 1H), 4.16 (q, *J* = 7.1 Hz, 2H), 2.81 (q, *J* = 7.4 Hz, 2H), 2.63 (q, *J* = 13.7 Hz, 2H), 1.75 – 1.62 (m, 1H), 1.43 – 1.32 (m, 1H), 1.30 – 1.17 (m, 10H), 1.13 (s, 3H), 0.85 (t, *J* = 6.9 Hz, 3H); ^{13}C NMR (75 MHz, $CDCl_3$) δ 205.79, 167.77, 137.17, 128.15, 60.96, 53.72, 40.07, 39.68, 26.49, 23.33, 23.24, 20.50, 14.76, 14.30, 14.08, 1.16; HRMS TOF MS ES+: $[MH]^+$ calcd. for $C_{15}H_{27}O_3S$ 287.1681; found 287.1655.

Ethyl (*S*)-4-((ethylthio)carbonyl)-4-methyl-2-methylene-7-((triisopropylsilyl)oxy) heptanoate (**5m**)

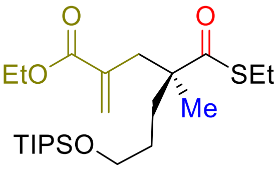

The product was obtained as a colorless oil (72% over 3 steps, *e.r.* 99:1). $[\alpha]_D^{20}$ = + 0.98 (*c* = 0.64, CH_2Cl_2); ^1H NMR (300 MHz, $CDCl_3$) δ 6.19 (d, *J* = 1.5 Hz, 1H), 5.51 (s, 1H), 4.15 (q, *J* = 7.1 Hz, 2H), 3.67 – 3.54 (m, 2H), 2.80 (q, *J* = 7.4 Hz, 2H), 2.64 (dd, J^1 = 32.9 Hz, J^2 = 13.7 Hz, 2H), 1.82 – 1.66 (m, 1H), 1.55 – 1.36 (m, 3H), 1.26 (t, *J* = 7.1 Hz, 3H), 1.19 (t, *J* = 7.4 Hz, 3H), 1.14 (s, 3H), 1.05 – 0.94 (m, 21H); ^{13}C NMR (75 MHz, $CDCl_3$) δ 205.60, 167.70, 137.05, 128.26, 63.62, 60.95, 53.44, 40.00, 36.10, 27.95, 23.24, 20.56, 18.15 (*6), 14.74, 14.29, 12.08 (*3); HRMS TOF MS ES+: $[MH]^+$ calcd. for $C_{23}H_{45}O_4SSi$ 445.2808; found 445.2813.

References

[1] C.-D. Lu and A. Zakarian, *Org. Synth.* 2008, **85**, 158-171.

[2] P. H. Dussault, C. T. Eary and K. R. Woller, *J. Org. Chem.* 1999, **64**, 1789-1797.

[3] M. Niggemann, A. Jelonek, N. Biber, M. Wuchrer and B. Plietker, *J. Org. Chem.* 2008, **73**, 7028-7036.

[4] J. Wu, X. Yang, Z. He, X. Mao, T. A. Hatton and T. F. Jamison, *Angew, Chem. Int. Ed.* 2014, **53**, 8416-8420.

[5] (a) C. Yu, B. Liu and L. Hu, *J. Org. Chem.* 2001, **66**, 5413−5418; (b) J. Villieras and M. Rambaud, *Synthesis* 1982, 924−926.

[6] B. M. O'Leary, T. Szabo, N. Svenstrup, C. A. Schalley, A. Lützen, M. Schäfer and J. Rebek, *J. Am. Chem. Soc.,* 2001, **123**, 11519-11533

[7] (a) B. C. Gerwick, W. K. Brewster, G. J. deBoer, S. C. Fields, P. R. Graupner, D. R. Hahn, C. J. Pearce, P. R. Schmitzer and J. D. Webster, *J. Chem. Ecol.* 2013, **39**, 253–261; (b) F. Ramon, M. Degueil-Castaing, M. Bevilacqua and B. Maillard, *Eur. Polym. J.* 1999, **35**, 547-556.

[8] Y. Minko, M. Pasco, L. Lercher, M. Botoshansky and I. Marek, *Nature*, 2012, **490**, 522-526.

[9] M. O. Frederick, J. A. Mulder, M. R. Tracey, R. P. Hsung, J. Huang, K. C. Kurtz, L. Shen and C. J. Douglas, *J. Am. Chem, Soc.* 2003, **125**, 2368-2369.

[10] (a) T. Bixa, R. Hunter, A. Andrijevic, W. Petersen, H. Su and F. Dhoro, *J. Org. Chem.,* 2015, **80**, 762; (b) X. Cui, X. Xu, H. Lu, S. Zhu, L. Wojtas and X. P. Zhang, *J. Am. Chem. Soc.,* 2011, **133**, 3304; (c) A. L. Krasovsky, V. G. Nenajdenko and E. S. Balenkova, *Tetrahedron*, 2001, **57**, 201.

X-Ray crystallographic analysis data of 4p

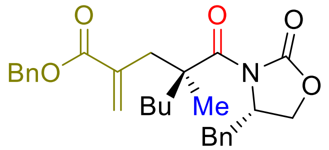

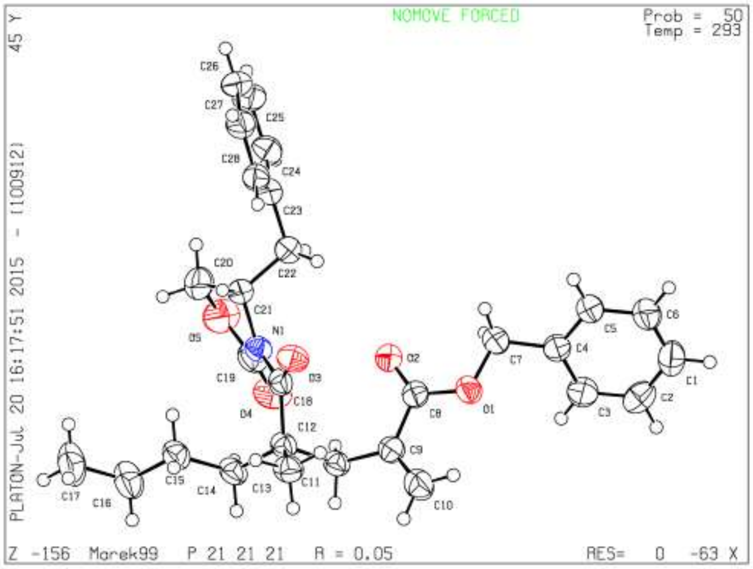

Supplementary Figure 4: Crystal structure of compound 4p

Table 1. Crystal data and structure refinement for Marek99.

Identification code	Marek99	
Empirical formula	C28 H33 N O5	
Formula weight	463.55	
Temperature	293(2) K	
Wavelength	0.71073 A	
Crystal system, space group	Orthorhombic, P 21 21 21	
Unit cell dimensions	a = 6.003(6) A	alpha = 90 deg.
	b = 14.84(3) A	beta = 90 deg.
	c = 28.93(3) A	gamma = 90 deg.
Volume	2577(7) A^3	

Z, Calculated density	4, 1.195 Mg/m^3
Absorption coefficient	0.081 mm^-1
F(000)	992
Crystal size	0.45 x 0.21 x 0.18 mm
Theta range for data collection	1.41 to 24.14 deg.
Limiting indices	0<=h<=6, 0<=k<=16, 0<=l<=32
Reflections collected / unique	2210 / 2210 [R(int) = 0.0650]
Completeness to theta = 24.14	99.1 %
Absorption correction	Semi-empirical from equivalents
Max. and min. transmission	0.9655 and 0.9643
Refinement method	Full-matrix least-squares on F^2
Data / restraints / parameters	2210 / 0 / 309
Goodness-of-fit on F^2	1.070
Final R indices [I>2sigma(I)]	R1 = 0.0460, wR2 = 0.1174
R indices (all data)	R1 = 0.0611, wR2 = 0.1403
Absolute structure parameter	0.0
Largest diff. peak and hole	0.196 and -0.214 e.A^-3

Table 2. Atomic coordinates (x 10^4) and equivalent isotropic displacement parameters (A^2 x 10^3) for shelxl. U(eq) is defined as one third of the trace of the orthogonalized Uij tensor.

	x	y	z	U(eq)
O(1)	-2690(4)	5907(2)	4144(1)	47(1)
O(2)	-1210(4)	4686(2)	3794(1)	50(1)
O(3)	3850(4)	5024(2)	3511(1)	49(1)
O(4)	-839(5)	3511(2)	2745(1)	66(1)
O(5)	1619(6)	2428(2)	2923(1)	71(1)

N(1)	2471(5)	3824(2)	3150(1)	43(1)
C(1)	-8088(8)	6678(3)	5386(2)	62(1)
C(2)	-8446(7)	6626(3)	4918(2)	63(1)
C(3)	-6905(7)	6205(3)	4635(1)	56(1)
C(4)	-4993(6)	5831(2)	4818(1)	45(1)
C(5)	-4643(7)	5894(3)	5292(1)	51(1)
C(6)	-6211(8)	6309(3)	5571(1)	61(1)
C(7)	-3373(7)	5334(3)	4520(1)	51(1)
C(8)	-1709(6)	5479(3)	3790(1)	42(1)
C(9)	-1325(6)	6077(3)	3382(1)	43(1)
C(10)	-1545(8)	6964(3)	3418(2)	64(1)
C(11)	-781(6)	5611(3)	2934(1)	43(1)
C(12)	1741(6)	5424(3)	2837(1)	41(1)
C(13)	3008(6)	6318(3)	2888(1)	49(1)
C(14)	1959(6)	5082(3)	2335(1)	49(1)
C(15)	4296(7)	4795(3)	2181(1)	53(1)
C(16)	4344(8)	4432(4)	1692(1)	72(1)
C(17)	6664(9)	4136(4)	1536(2)	92(2)
C(18)	2695(6)	4765(2)	3195(1)	41(1)
C(19)	916(8)	3294(3)	2917(1)	55(1)
C(20)	3870(8)	2402(3)	3113(2)	69(1)
C(21)	3975(7)	3225(3)	3416(1)	46(1)
C(22)	3129(6)	3047(3)	3908(1)	48(1)
C(23)	4662(6)	2441(3)	4178(1)	45(1)
C(24)	4097(7)	1554(3)	4280(1)	57(1)
C(25)	5538(8)	1005(3)	4528(2)	67(1)
C(26)	7563(8)	1337(3)	4682(2)	63(1)
C(27)	8143(7)	2221(3)	4584(1)	54(1)
C(28)	6721(6)	2756(3)	4329(1)	47(1)

Table 3. Bond lengths [A] and angles [deg] for shelxl.

O(1)-C(8)	1.342(4)
O(1)-C(7)	1.440(5)
O(2)-C(8)	1.214(5)
O(3)-C(18)	1.210(4)
O(4)-C(19)	1.208(5)
O(5)-C(19)	1.354(6)
O(5)-C(20)	1.459(6)
N(1)-C(19)	1.394(5)
N(1)-C(18)	1.409(6)
N(1)-C(21)	1.482(5)
C(1)-C(6)	1.363(6)
C(1)-C(2)	1.373(6)
C(1)-H(1)	0.9300
C(2)-C(3)	1.383(6)
C(2)-H(2)	0.9300
C(3)-C(4)	1.381(6)
C(3)-H(3)	0.9300
C(4)-C(5)	1.389(5)
C(4)-C(7)	1.495(5)
C(5)-C(6)	1.386(6)
C(5)-H(5)	0.9300
C(6)-H(6)	0.9300
C(7)-H(7A)	0.9700
C(7)-H(7B)	0.9700
C(8)-C(9)	1.494(5)
C(9)-C(10)	1.327(6)
C(9)-C(11)	1.506(5)
C(10)-H(10A)	0.9300

C(10)-H(10B)	0.9300
C(11)-C(12)	1.564(5)
C(11)-H(11A)	0.9700
C(11)-H(11B)	0.9700
C(12)-C(18)	1.536(5)
C(12)-C(13)	1.536(6)
C(12)-C(14)	1.543(5)
C(13)-H(13A)	0.9600
C(13)-H(13B)	0.9600
C(13)-H(13C)	0.9600
C(14)-C(15)	1.533(6)
C(14)-H(14A)	0.9700
C(14)-H(14B)	0.9700
C(15)-C(16)	1.514(6)
C(15)-H(15A)	0.9700
C(15)-H(15B)	0.9700
C(16)-C(17)	1.528(7)
C(16)-H(16A)	0.9700
C(16)-H(16B)	0.9700
C(17)-H(17A)	0.9600
C(17)-H(17B)	0.9600
C(17)-H(17C)	0.9600
C(20)-C(21)	1.505(6)
C(20)-H(20A)	0.9700
C(20)-H(20B)	0.9700
C(21)-C(22)	1.533(5)
C(21)-H(21)	0.9800
C(22)-C(23)	1.506(5)
C(22)-H(22A)	0.9700
C(22)-H(22B)	0.9700
C(23)-C(24)	1.390(6)

C(23)-C(28)	1.392(6)
C(24)-C(25)	1.387(6)
C(24)-H(24)	0.9300
C(25)-C(26)	1.386(7)
C(25)-H(25)	0.9300
C(26)-C(27)	1.385(7)
C(26)-H(26)	0.9300
C(27)-C(28)	1.379(5)
C(27)-H(27)	0.9300
C(28)-H(28)	0.9300
C(8)-O(1)-C(7)	115.0(3)
C(19)-O(5)-C(20)	108.6(4)
C(19)-N(1)-C(18)	131.8(3)
C(19)-N(1)-C(21)	108.8(3)
C(18)-N(1)-C(21)	119.2(3)
C(6)-C(1)-C(2)	119.7(4)
C(6)-C(1)-H(1)	120.2
C(2)-C(1)-H(1)	120.2
C(1)-C(2)-C(3)	120.2(4)
C(1)-C(2)-H(2)	119.9
C(3)-C(2)-H(2)	119.9
C(4)-C(3)-C(2)	120.7(4)
C(4)-C(3)-H(3)	119.6
C(2)-C(3)-H(3)	119.6
C(3)-C(4)-C(5)	118.5(4)
C(3)-C(4)-C(7)	121.1(3)
C(5)-C(4)-C(7)	120.3(4)
C(6)-C(5)-C(4)	120.2(4)
C(6)-C(5)-H(5)	119.9
C(4)-C(5)-H(5)	119.9

C(1)-C(6)-C(5)	120.7(4)
C(1)-C(6)-H(6)	119.7
C(5)-C(6)-H(6)	119.7
O(1)-C(7)-C(4)	109.3(3)
O(1)-C(7)-H(7A)	109.8
C(4)-C(7)-H(7A)	109.8
O(1)-C(7)-H(7B)	109.8
C(4)-C(7)-H(7B)	109.8
H(7A)-C(7)-H(7B)	108.3
O(2)-C(8)-O(1)	124.0(3)
O(2)-C(8)-C(9)	123.2(3)
O(1)-C(8)-C(9)	112.9(3)
C(10)-C(9)-C(8)	120.9(4)
C(10)-C(9)-C(11)	123.0(3)
C(8)-C(9)-C(11)	116.1(3)
C(9)-C(10)-H(10A)	120.0
C(9)-C(10)-H(10B)	120.0
H(10A)-C(10)-H(10B)	120.0
C(9)-C(11)-C(12)	116.4(3)
C(9)-C(11)-H(11A)	108.2
C(12)-C(11)-H(11A)	108.2
C(9)-C(11)-H(11B)	108.2
C(12)-C(11)-H(11B)	108.2
H(11A)-C(11)-H(11B)	107.3
C(18)-C(12)-C(13)	107.5(3)
C(18)-C(12)-C(14)	113.1(3)
C(13)-C(12)-C(14)	109.4(3)
C(18)-C(12)-C(11)	110.7(3)
C(13)-C(12)-C(11)	108.0(3)
C(14)-C(12)-C(11)	108.0(3)
C(12)-C(13)-H(13A)	109.5

C(12)-C(13)-H(13B)	109.5
H(13A)-C(13)-H(13B)	109.5
C(12)-C(13)-H(13C)	109.5
H(13A)-C(13)-H(13C)	109.5
H(13B)-C(13)-H(13C)	109.5
C(15)-C(14)-C(12)	116.3(3)
C(15)-C(14)-H(14A)	108.2
C(12)-C(14)-H(14A)	108.2
C(15)-C(14)-H(14B)	108.2
C(12)-C(14)-H(14B)	108.2
H(14A)-C(14)-H(14B)	107.4
C(16)-C(15)-C(14)	112.9(3)
C(16)-C(15)-H(15A)	109.0
C(14)-C(15)-H(15A)	109.0
C(16)-C(15)-H(15B)	109.0
C(14)-C(15)-H(15B)	109.0
H(15A)-C(15)-H(15B)	107.8
C(15)-C(16)-C(17)	113.3(4)
C(15)-C(16)-H(16A)	108.9
C(17)-C(16)-H(16A)	108.9
C(15)-C(16)-H(16B)	108.9
C(17)-C(16)-H(16B)	108.9
H(16A)-C(16)-H(16B)	107.7
C(16)-C(17)-H(17A)	109.5
C(16)-C(17)-H(17B)	109.5
H(17A)-C(17)-H(17B)	109.5
C(16)-C(17)-H(17C)	109.5
H(17A)-C(17)-H(17C)	109.5
H(17B)-C(17)-H(17C)	109.5
O(3)-C(18)-N(1)	116.1(3)
O(3)-C(18)-C(12)	121.3(3)

N(1)-C(18)-C(12)	122.3(3)
O(4)-C(19)-O(5)	122.0(4)
O(4)-C(19)-N(1)	129.3(4)
O(5)-C(19)-N(1)	108.7(4)
O(5)-C(20)-C(21)	103.7(3)
O(5)-C(20)-H(20A)	111.0
C(21)-C(20)-H(20A)	111.0
O(5)-C(20)-H(20B)	111.0
C(21)-C(20)-H(20B)	111.0
H(20A)-C(20)-H(20B)	109.0
N(1)-C(21)-C(20)	99.1(3)
N(1)-C(21)-C(22)	112.5(3)
C(20)-C(21)-C(22)	112.7(4)
N(1)-C(21)-H(21)	110.7
C(20)-C(21)-H(21)	110.7
C(22)-C(21)-H(21)	110.7
C(23)-C(22)-C(21)	112.5(3)
C(23)-C(22)-H(22A)	109.1
C(21)-C(22)-H(22A)	109.1
C(23)-C(22)-H(22B)	109.1
C(21)-C(22)-H(22B)	109.1
H(22A)-C(22)-H(22B)	107.8
C(24)-C(23)-C(28)	117.9(4)
C(24)-C(23)-C(22)	121.8(4)
C(28)-C(23)-C(22)	120.3(4)
C(25)-C(24)-C(23)	120.9(4)
C(25)-C(24)-H(24)	119.6
C(23)-C(24)-H(24)	119.6
C(26)-C(25)-C(24)	120.3(4)
C(26)-C(25)-H(25)	119.9
C(24)-C(25)-H(25)	119.9

C(27)-C(26)-C(25)	119.4(4)
C(27)-C(26)-H(26)	120.3
C(25)-C(26)-H(26)	120.3
C(28)-C(27)-C(26)	119.9(4)
C(28)-C(27)-H(27)	120.0
C(26)-C(27)-H(27)	120.0
C(27)-C(28)-C(23)	121.6(4)
C(27)-C(28)-H(28)	119.2
C(23)-C(28)-H(28)	119.2

Symmetry transformations used to generate equivalent atoms:

Table 4. Anisotropic displacement parameters (A^2 x 10^3) for shelxl. The anisotropic displacement factor exponent takes the form: -2 pi^2 [h^2 a*^2 U11 + ... + 2 h k a* b* U12]

	U11	U22	U33	U23	U13	U12
O(1)	57(2)	47(1)	38(1)	-3(1)	4(1)	2(1)
O(2)	51(2)	49(2)	49(2)	5(1)	8(1)	7(1)
O(3)	49(2)	56(2)	41(1)	2(1)	-13(1)	-5(1)
O(4)	68(2)	68(2)	61(2)	1(2)	-20(2)	-16(2)
O(5)	98(2)	54(2)	62(2)	-16(2)	-19(2)	4(2)
N(1)	48(2)	47(2)	36(2)	0(1)	-4(1)	-1(2)
C(1)	62(3)	58(3)	67(3)	-9(2)	17(2)	3(2)
C(2)	48(2)	58(3)	82(3)	0(2)	-5(2)	7(2)
C(3)	55(2)	60(3)	53(2)	-2(2)	-5(2)	0(2)
C(4)	52(2)	37(2)	47(2)	1(2)	5(2)	-1(2)
C(5)	56(2)	52(2)	43(2)	-1(2)	2(2)	6(2)
C(6)	76(3)	60(3)	47(2)	-5(2)	6(2)	8(2)
C(7)	61(2)	50(2)	42(2)	3(2)	9(2)	6(2)
C(8)	36(2)	49(2)	42(2)	-2(2)	-3(2)	0(2)
C(9)	38(2)	47(2)	42(2)	2(2)	-1(2)	0(2)
C(10)	76(3)	59(3)	57(2)	6(2)	16(2)	0(2)
C(11)	41(2)	52(2)	36(2)	4(2)	-2(2)	0(2)
C(12)	36(2)	53(2)	35(2)	2(2)	-2(2)	-1(2)
C(13)	43(2)	54(2)	51(2)	9(2)	-1(2)	-2(2)
C(14)	44(2)	68(3)	34(2)	4(2)	-3(2)	0(2)
C(15)	53(2)	68(3)	39(2)	0(2)	1(2)	2(2)

C(16)	66(3)	100(4)	49(2)	-14(3)	5(2)	-1(3)
C(17)	83(3)	135(5)	59(3)	-26(3)	14(3)	15(4)
C(18)	34(2)	50(2)	38(2)	-1(2)	4(2)	-1(2)
C(19)	71(3)	54(3)	41(2)	-5(2)	-2(2)	-3(2)
C(20)	85(4)	67(3)	56(2)	-10(2)	3(2)	21(3)
C(21)	46(2)	50(2)	42(2)	6(2)	2(2)	10(2)
C(22)	47(2)	52(2)	43(2)	4(2)	3(2)	5(2)
C(23)	47(2)	47(2)	39(2)	1(2)	6(2)	6(2)
C(24)	57(2)	50(2)	64(2)	6(2)	5(2)	-6(2)
C(25)	75(3)	48(2)	77(3)	18(2)	11(3)	3(2)
C(26)	67(3)	59(3)	62(3)	20(2)	7(2)	18(2)
C(27)	50(2)	65(3)	48(2)	4(2)	-3(2)	5(2)
C(28)	51(2)	42(2)	48(2)	3(2)	6(2)	0(2)

Table 5. Hydrogen coordinates (x 10^4) and isotropic displacement parameters (A^2 x 10^3) for shelxl.

	x	y	z	U(eq)
H(1)	-9122	6964	5575	75
H(2)	-9727	6876	4790	75
H(3)	-7161	6172	4319	67
H(5)	-3352	5657	5422	61
H(6)	-5981	6337	5889	73
H(7A)	-2083	5159	4701	61
H(7B)	-4063	4793	4399	61
H(10A)	-1943	7220	3699	77
H(10B)	-1301	7329	3162	77
H(11A)	-1362	5974	2682	51
H(11B)	-1565	5039	2928	51
H(13A)	4550	6226	2814	74
H(13B)	2882	6529	3201	74
H(13C)	2384	6757	2682	74
H(14A)	965	4572	2297	58
H(14B)	1446	5554	2129	58
H(15A)	5289	5309	2201	64
H(15B)	4847	4335	2390	64
H(16A)	3803	4893	1483	86
H(16B)	3339	3921	1671	86
H(17A)	6578	3887	1230	138
H(17B)	7225	3687	1745	138
H(17C)	7645	4646	1535	138
H(20A)	4976	2431	2869	83

H(20B)	4106	1858	3292	83
H(21)	5490	3471	3423	55
H(22A)	2979	3616	4069	57
H(22B)	1666	2772	3891	57
H(24)	2734	1327	4182	69
H(25)	5144	412	4590	80
H(26)	8524	971	4850	75
H(27)	9489	2452	4689	65
H(28)	7147	3342	4257	56

Table 6. Torsion angles [deg] for shelxl.

Analytical HPLC analysis data for the products of enantioselective allylic alkylation

Compound **4b**

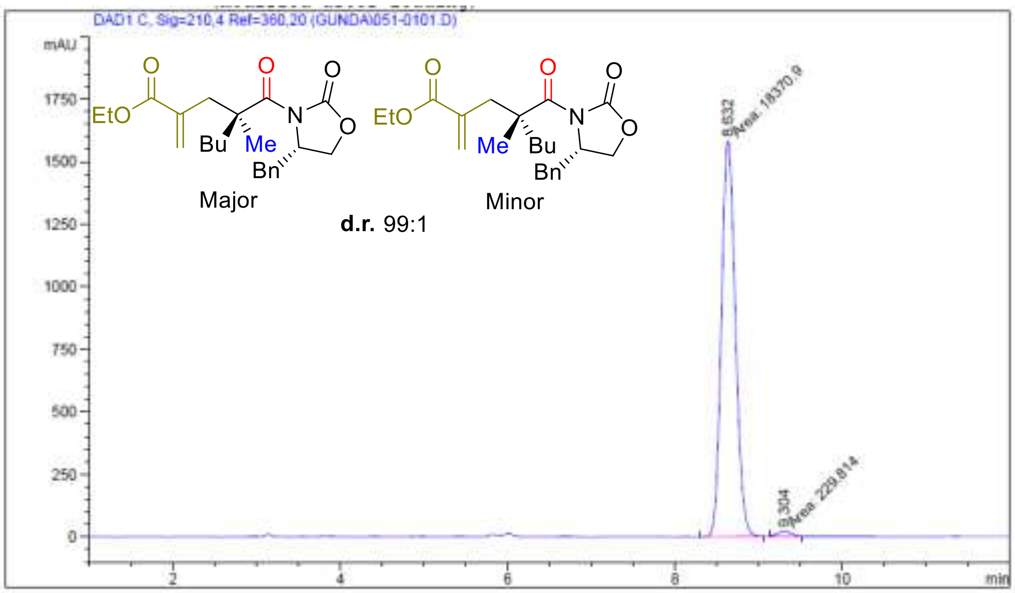

Peak #	RetTime [min]	Type	Width [min]	Area [mAU*s]	Height [mAU]	Area %
1	8.632	MM	0.1934	1.83709e4	1583.38513	98.7645
2	9.304	MM	0.1909	229.81447	20.06591	1.2355
Totals :				1.86007e4	1603.45104	

Compound **4c**

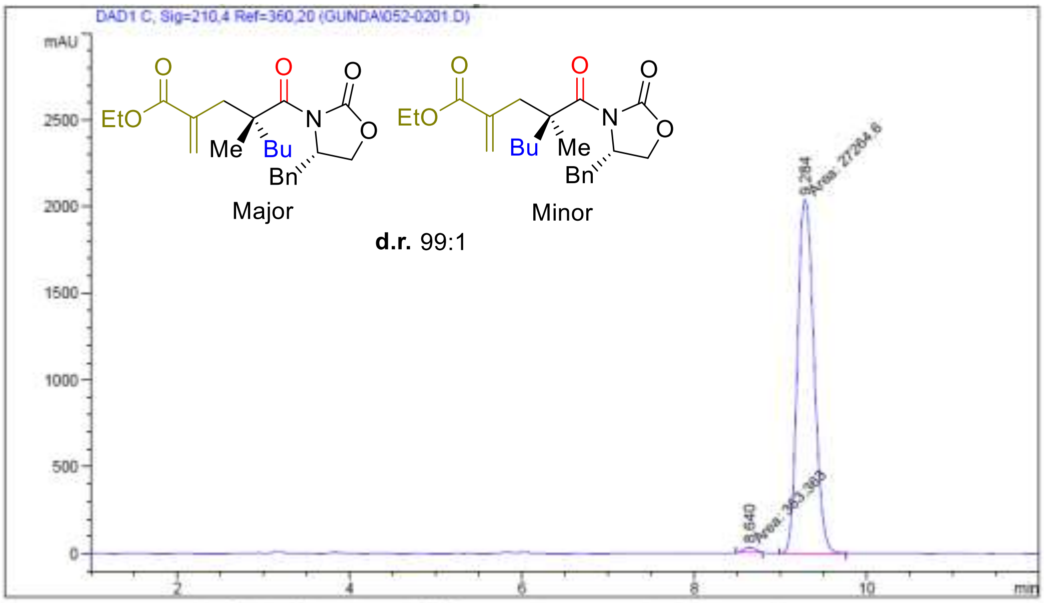

Peak #	RetTime [min]	Type	Width [min]	Area [mAU*s]	Height [mAU]	Area %
1	8.640	MM	0.1740	363.36325	34.80334	1.3152
2	9.284	MM	0.2225	2.72646e4	2042.09912	98.6848
Totals :				2.76280e4	2076.90246	

Compound **4b** and **4c**

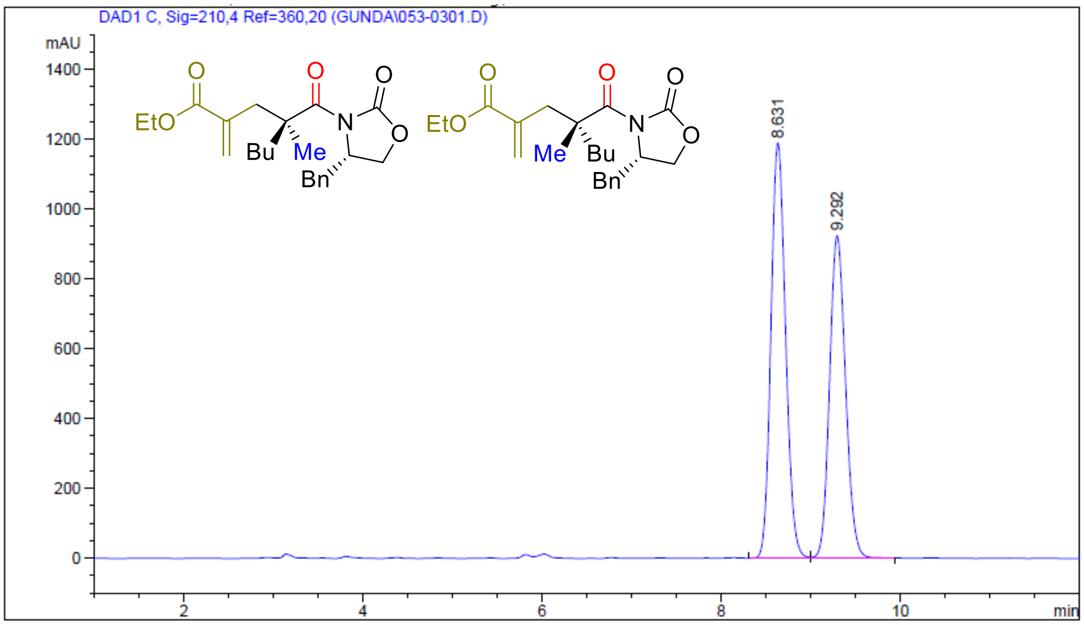

Peak #	RetTime [min]	Type	Width [min]	Area [mAU*s]	Height [mAU]	Area %
1	8.631	VV	0.1763	1.34952e4	1190.31921	54.0563
2	9.292	VB	0.1927	1.14699e4	924.84631	45.9437

Totals : 2.49652e4 2115.16553

v

Compound **4d**

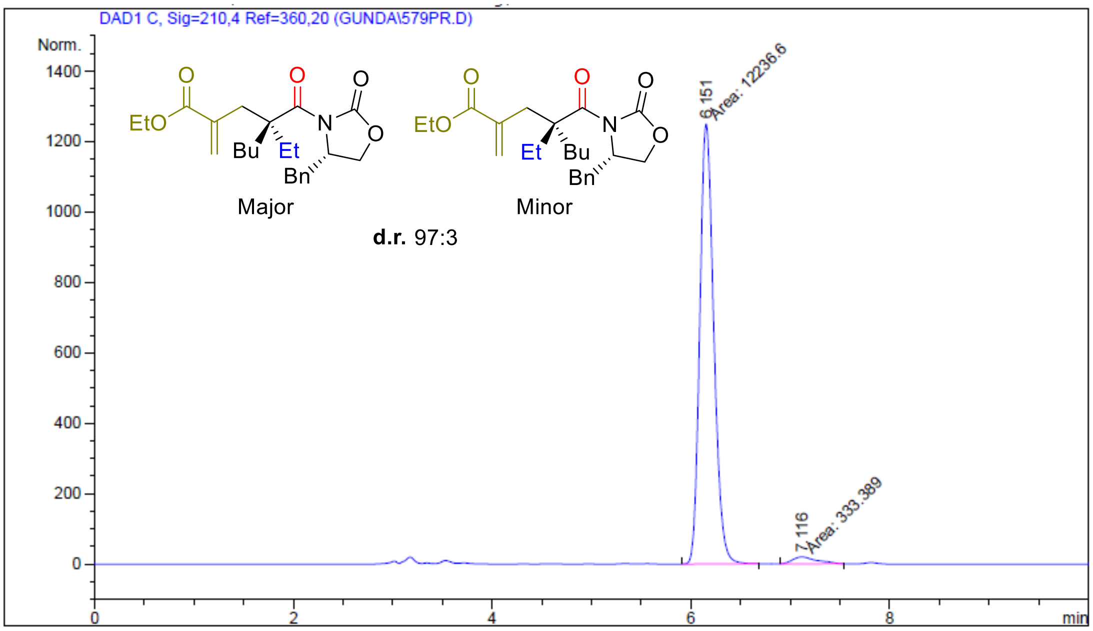

Peak #	RetTime [min]	Type	Width [min]	Area [mAU*s]	Height [mAU]	Area %
1	6.151	MM	0.1631	1.22366e4	1250.45911	97.3477
2	7.116	MM	0.2836	333.38879	19.59052	2.6523

Totals : 1.25700e4 1270.04963

Compound **4e**

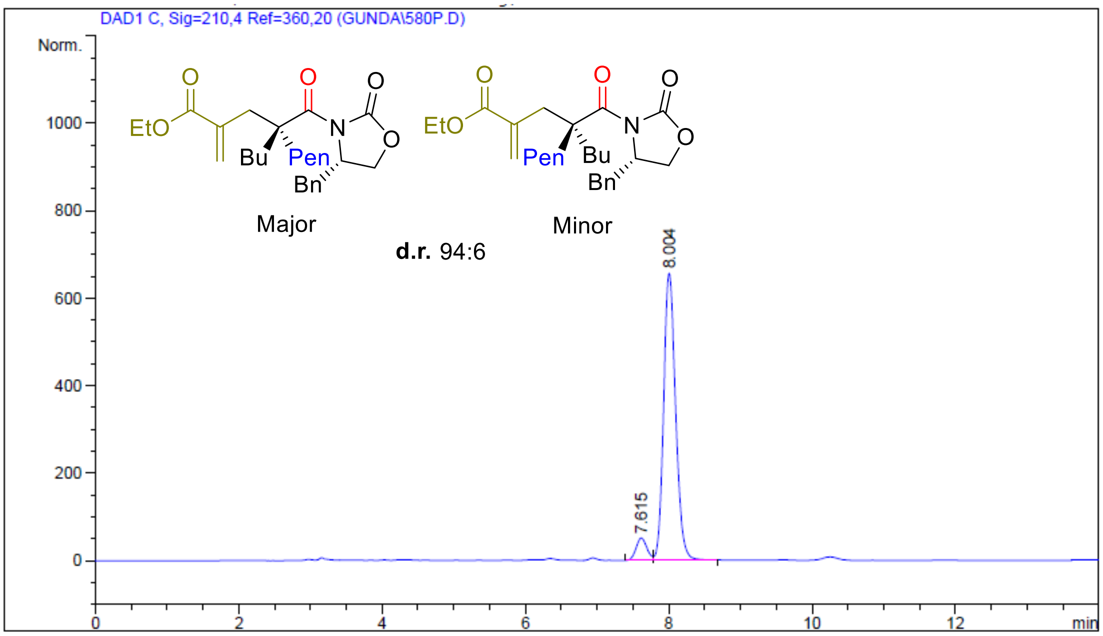

Peak #	RetTime [min]	Type	Width [min]	Area [mAU*s]	Height [mAU]	Area %
1	7.615	PV	0.1624	536.76361	51.17183	6.5673
2	8.004	VB	0.1797	7636.47217	656.62415	93.4327

Totals : 8173.23578 707.79597

Compound **4f**

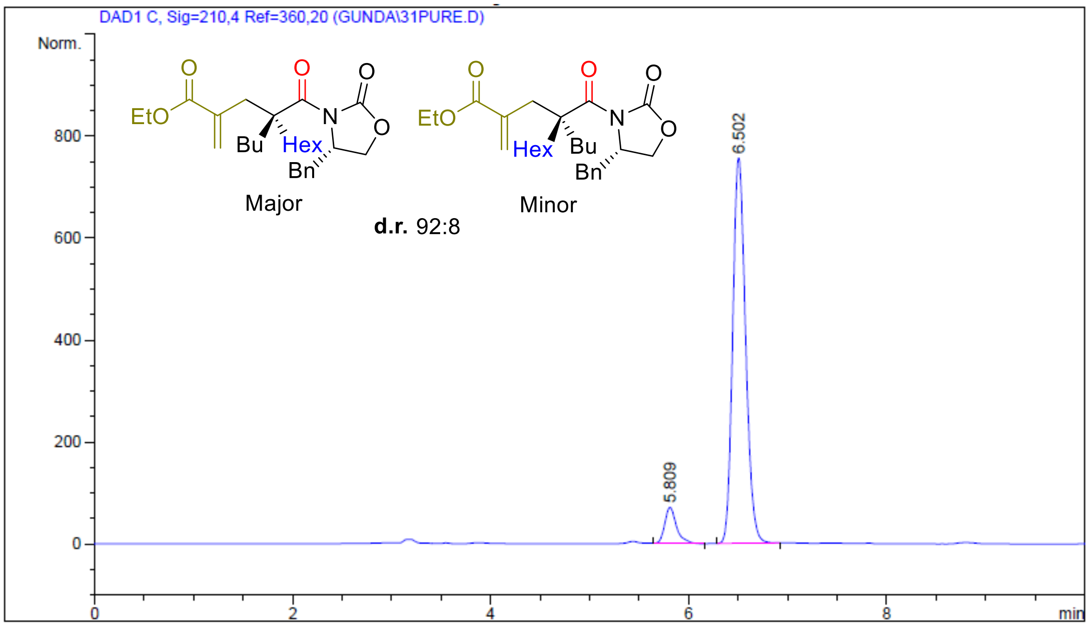

Peak #	RetTime [min]	Type	Width [min]	Area [mAU*s]	Height [mAU]	Area %
1	5.809	VP	0.1245	587.30554	70.99325	7.9184
2	6.502	BB	0.1393	6829.64258	756.60345	92.0816
Totals :				7416.94812	827.59670	

Compound **4g**

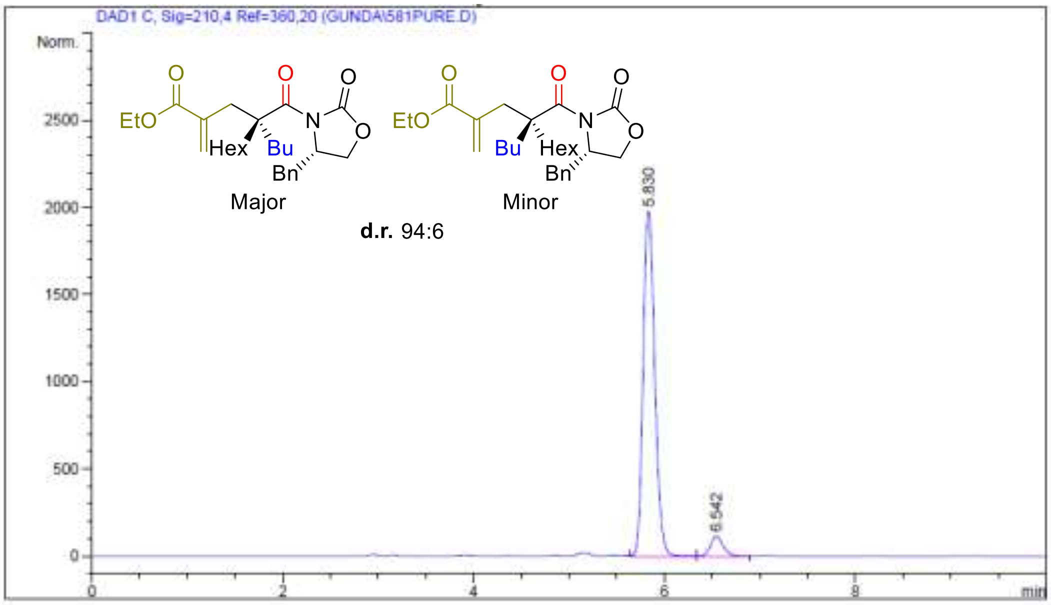

Peak #	RetTime [min]	Type	Width [min]	Area [mAU*s]	Height [mAU]	Area %
1	5.830	VV	0.1323	1.66833e4	1980.04260	93.9840
2	6.542	VV	0.1430	1067.91956	114.35373	6.0160
Totals :				1.77512e4	2094.39633	

Compounds **4g** and **4f**

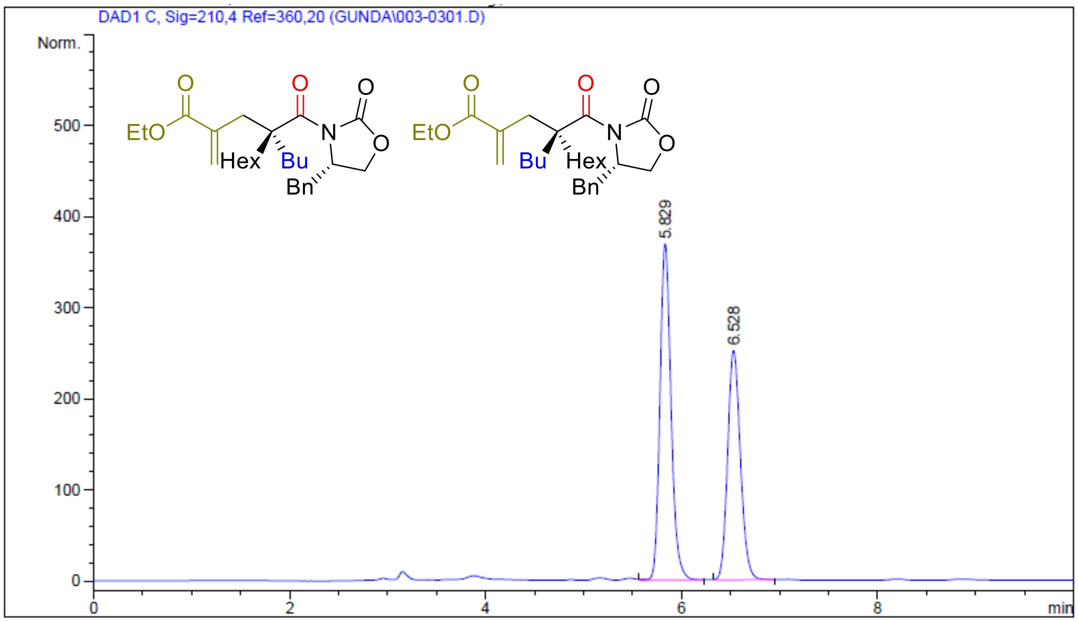

Peak #	RetTime [min]	Type	Width [min]	Area [mAU*s]	Height [mAU]	Area %
1	5.829	VB	0.1219	2908.48926	369.27066	55.9844
2	6.528	BB	0.1417	2286.68530	252.35075	44.0156

Totals : 5195.17456 621.62141

Compound **4h**

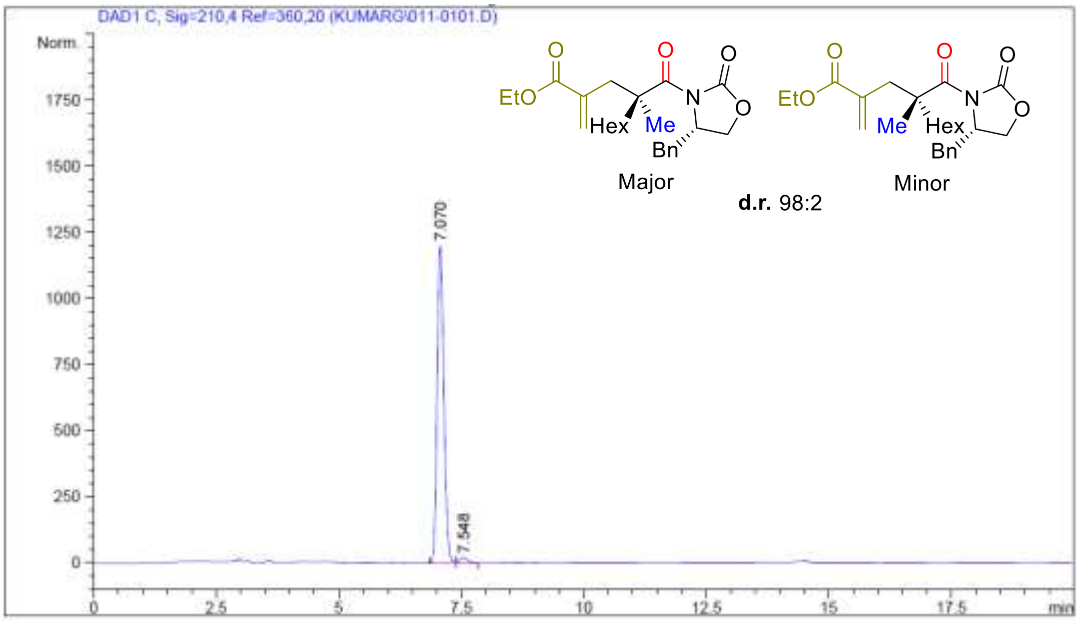

Peak #	RetTime [min]	Type	Width [min]	Area [mAU*s]	Height [mAU]	Area %
1	7.070	BV	0.1470	1.14160e4	1200.27759	97.9211
2	7.548	VV	0.1765	242.37015	20.71601	2.0789
Totals :				1.16584e4	1220.99359	

Compound **4i**

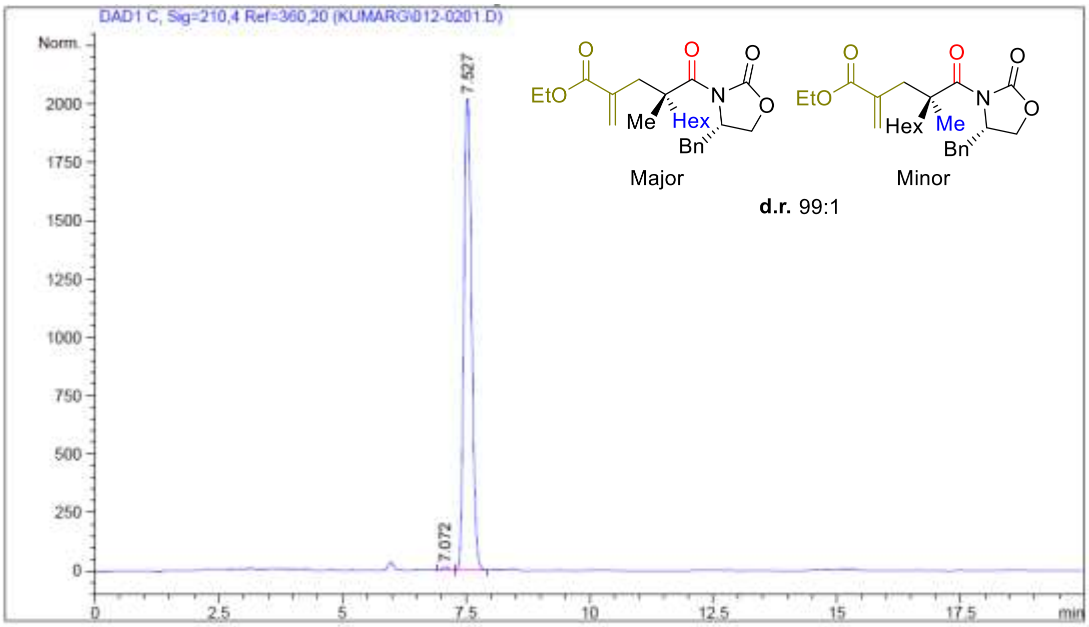

Peak #	RetTime [min]	Type	Width [min]	Area [mAU*s]	Height [mAU]	Area %
1	7.072	VV	0.1505	153.20645	15.60636	0.6842
2	7.527	VV	0.1741	2.22401e4	2025.18823	99.3158
Totals :				2.23933e4	2040.79460	

Compound **4h** and **4i**

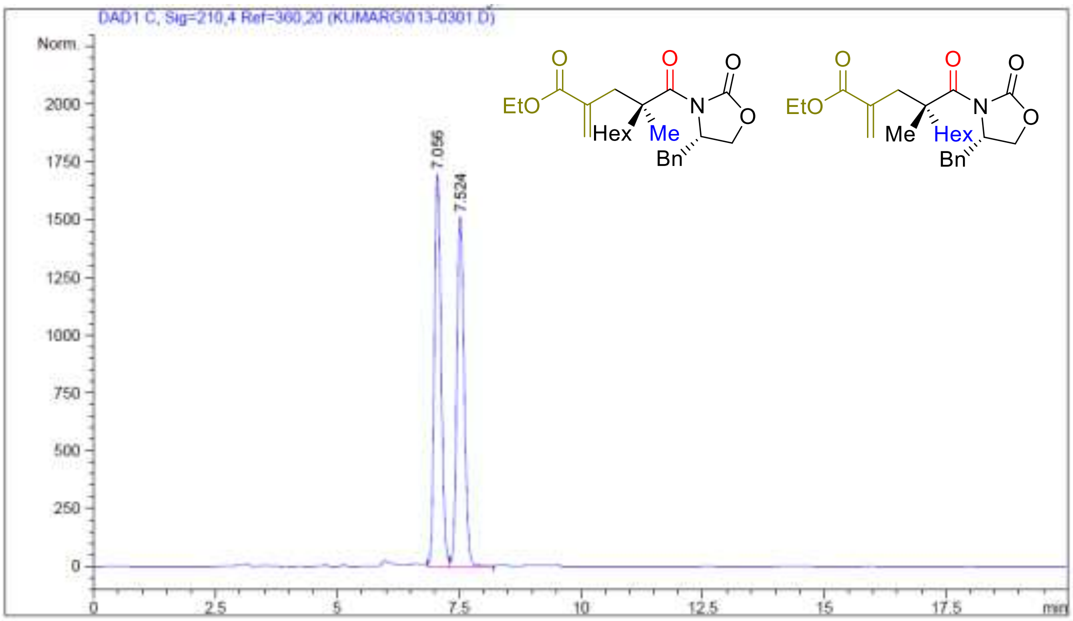

Peak #	RetTime [min]	Type	Width [min]	Area [mAU*s]	Height [mAU]	Area %
1	7.056	VV	0.1519	1.65791e4	1698.83167	51.0846
2	7.524	VV	0.1644	1.58751e4	1513.38733	48.9154

Totals : 3.24542e4 3212.21899

Compound **4j**

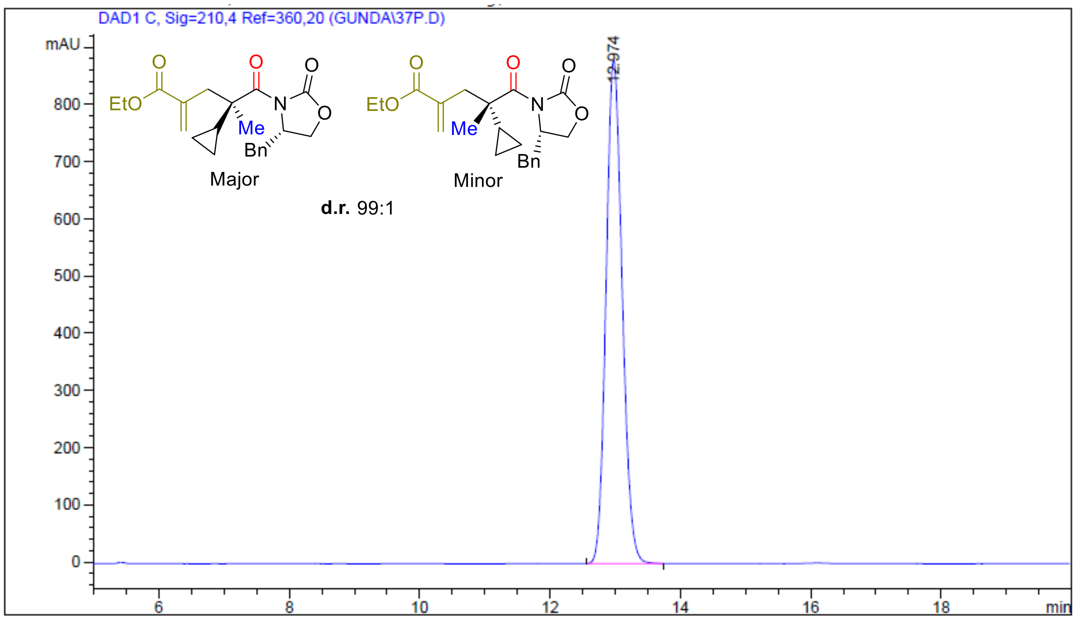

Peak #	RetTime [min]	Type	Width [min]	Area [mAU*s]	Height [mAU]	Area %
1	12.974	PB	0.2669	1.51528e4	880.16132	100.0000
Totals :				1.51528e4	880.16132	

Compound **4k**

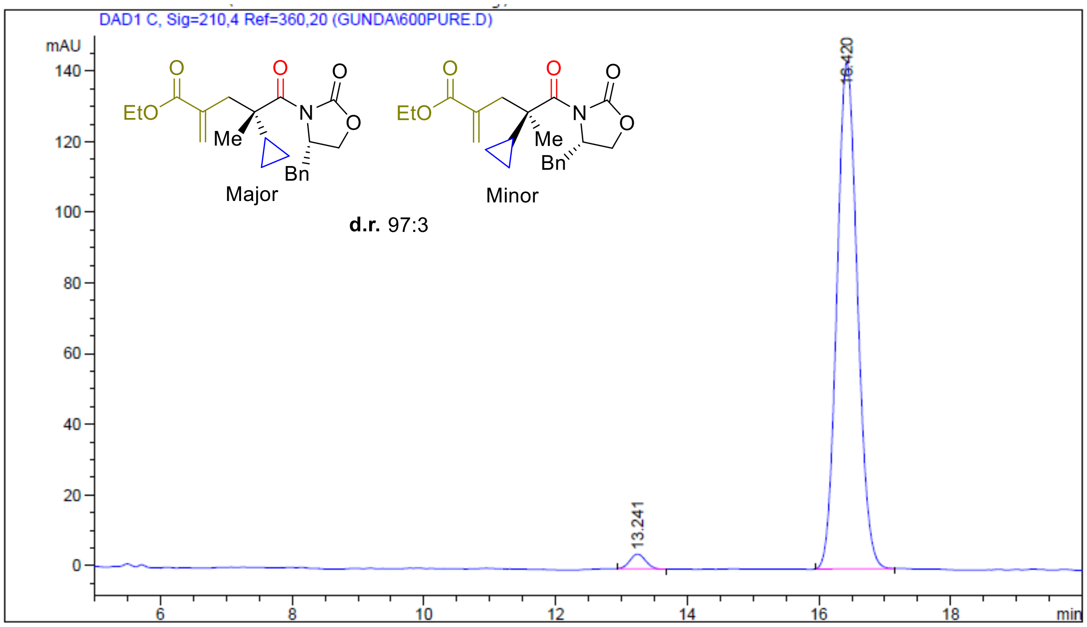

Peak #	RetTime [min]	Type	Width [min]	Area [mAU*s]	Height [mAU]	Area %
1	13.241	BP	0.2551	70.56165	4.21962	2.2207
2	16.420	BB	0.3364	3106.90381	143.59395	97.7793
Totals :				3177.46545	147.81357	

Compound **4j** and **4k**

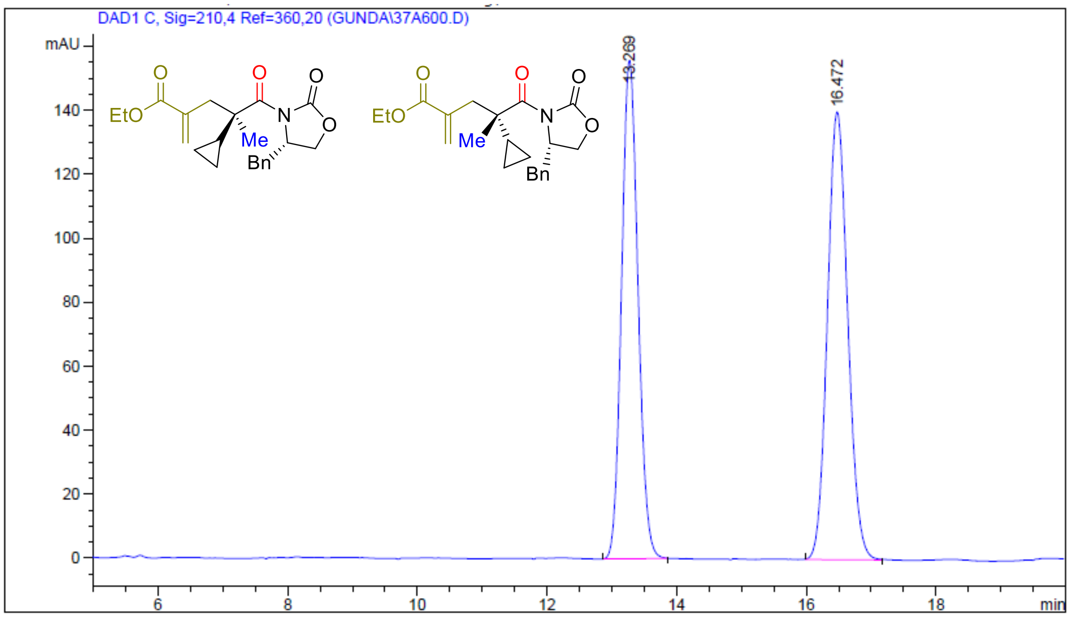

Peak #	RetTime [min]	Type	Width [min]	Area [mAU*s]	Height [mAU]	Area %
1	13.269	PB	0.2734	2745.83545	155.93945	47.3781
2	16.472	BB	0.3381	3049.74170	140.04997	52.6219

Totals : 5795.57715 295.98943

Compound **4l**

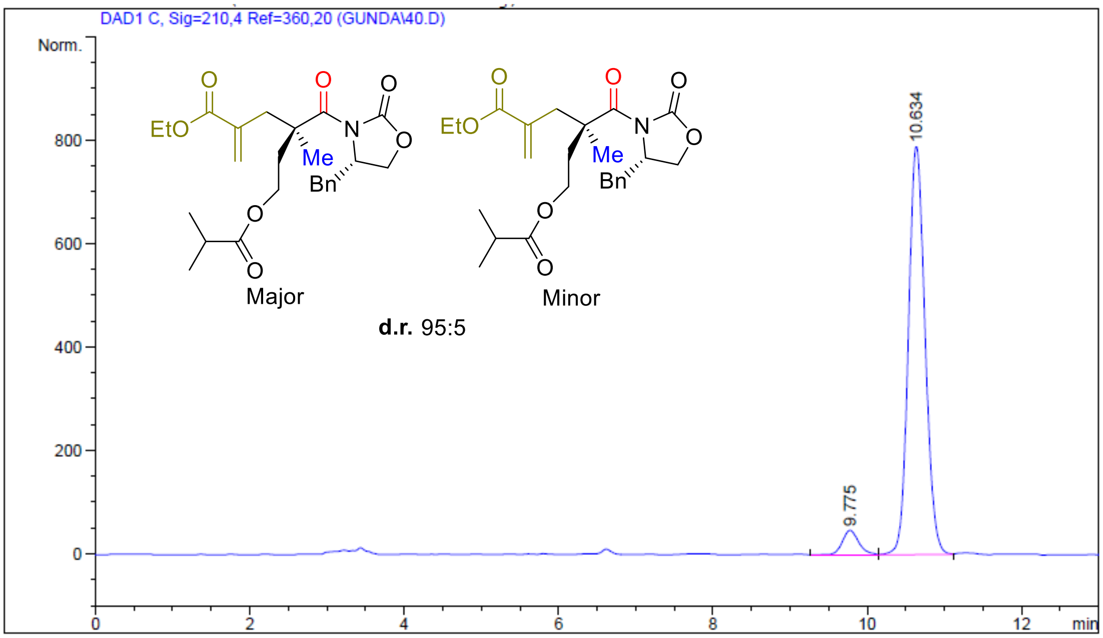

Peak #	RetTime [min]	Type	Width [min]	Area [mAU*s]	Height [mAU]	Area %
1	9.775	VV	0.2272	700.89154	47.21531	5.5791
2	10.634	VV	0.2333	1.18620e4	789.58185	94.4209
Totals :				1.25629e4	836.79716	

Compound **4m**

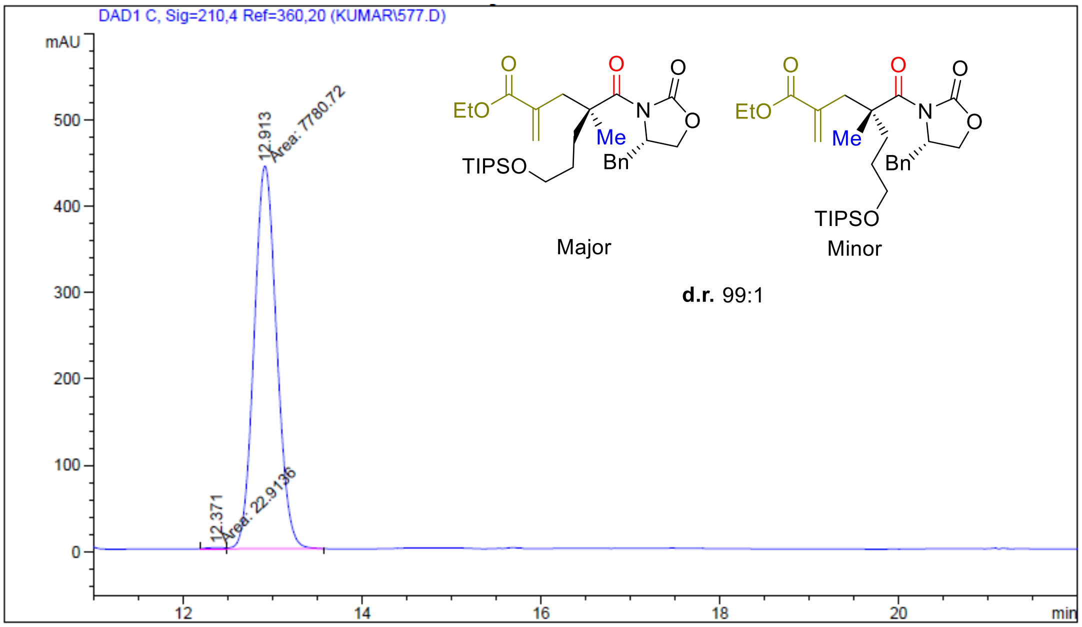

Peak #	RetTime [min]	Type	Width [min]	Area [mAU*s]	Height [mAU]	Area %
1	12.371	MF	0.2300	22.91360	1.66019	0.2936
2	12.913	FM	0.2926	7780.72021	443.19312	99.7064

Totals : 7803.63382 444.85331

Compound **4n**

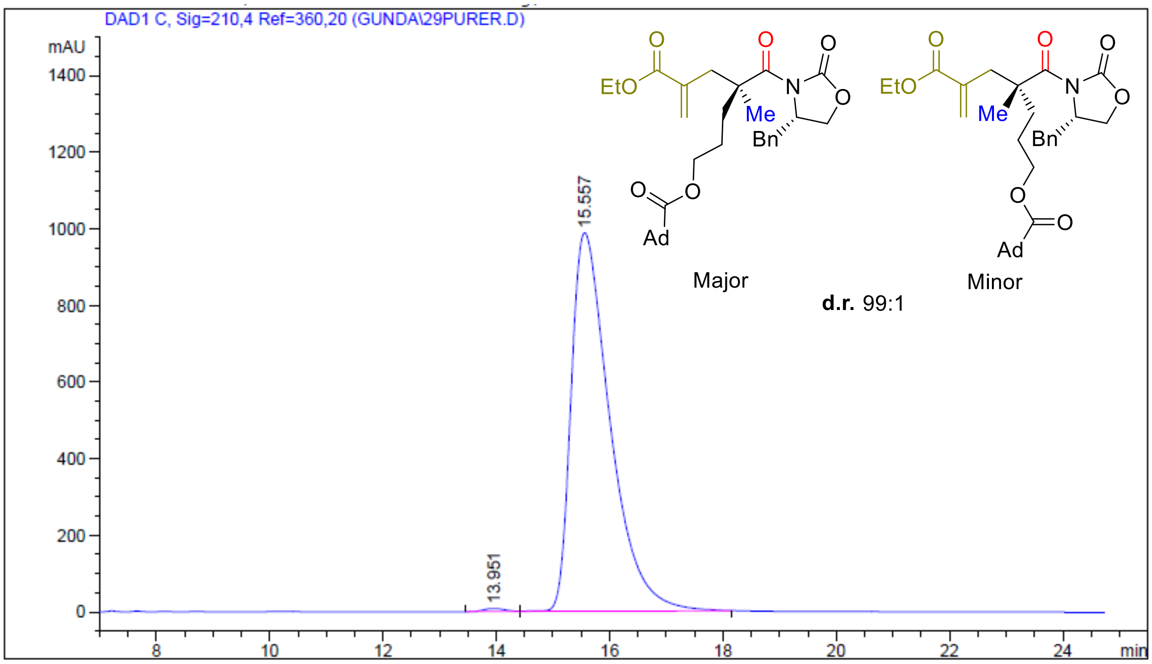

Peak #	RetTime [min]	Type	Width [min]	Area [mAU*s]	Height [mAU]	Area %
1	13.951	BV	0.4144	231.31723	8.58482	0.4872
2	15.557	VB	0.7296	4.72450e4	988.52643	99.5128

Totals : 4.74763e4 997.11125

Compound **4o**

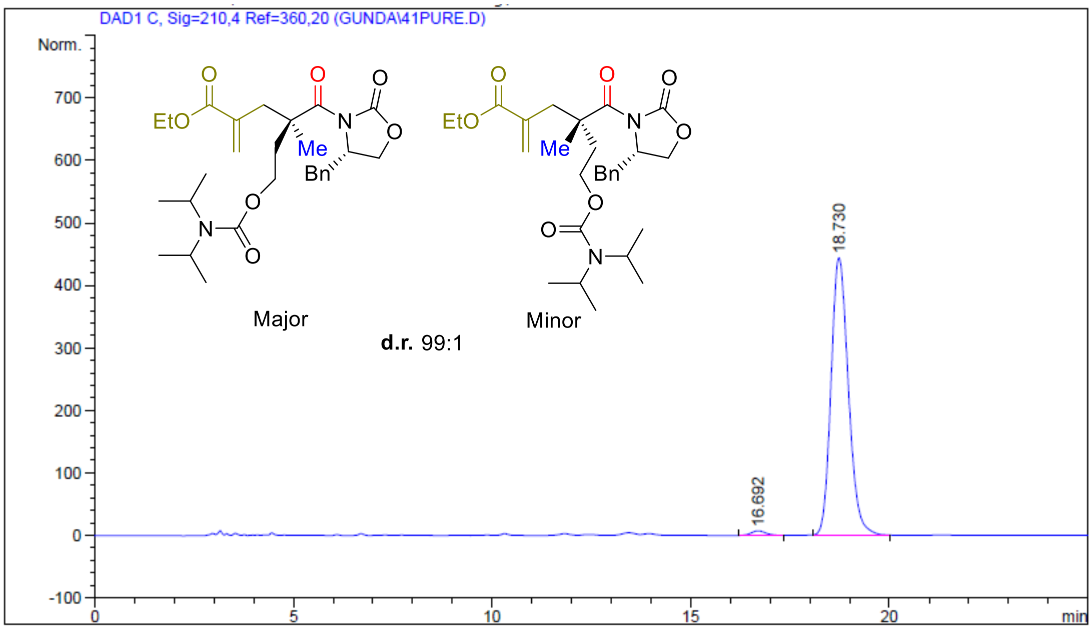

Peak #	RetTime [min]	Type	Width [min]	Area [mAU*s]	Height [mAU]	Area %
1	16.692	BB	0.3773	182.78043	7.37132	1.3375
2	18.730	BB	0.4700	1.34825e4	444.27875	98.6625
Totals :				1.36653e4	451.65007	

Compound **4p**

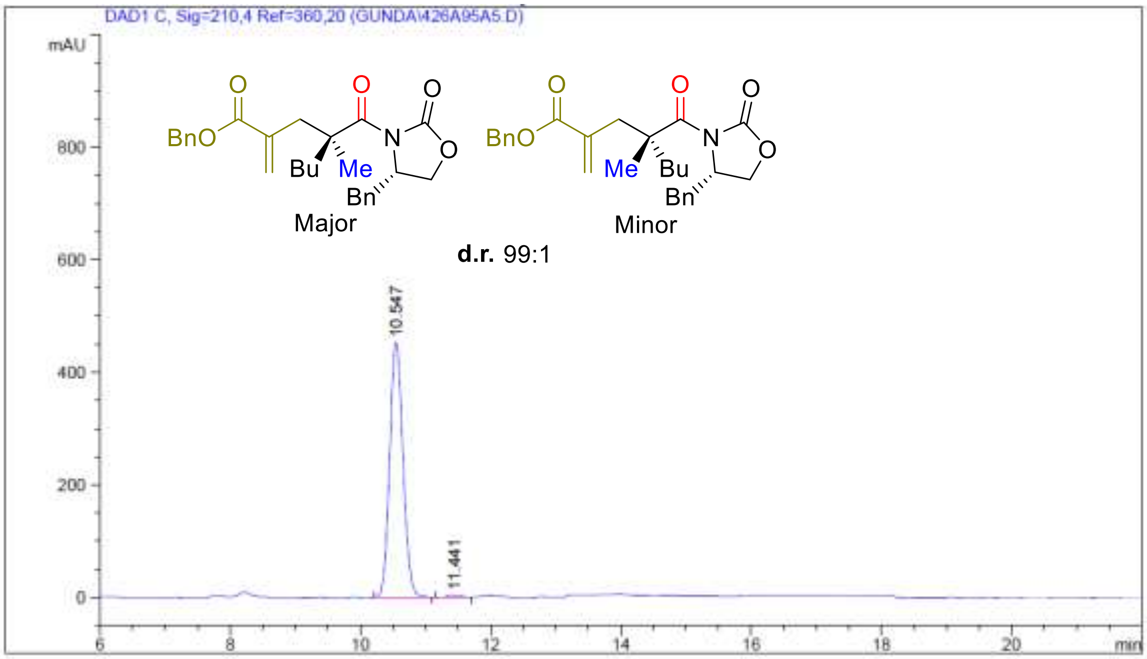

Peak #	RetTime [min]	Type	Width [min]	Area [mAU*s]	Height [mAU]	Area %
1	10.547	BB	0.2202	6470.37354	454.12494	98.9485
2	11.441	BV	0.2350	68.76198	4.43239	1.0515
Totals :				6539.13551	458.55733	

Compound **4q**

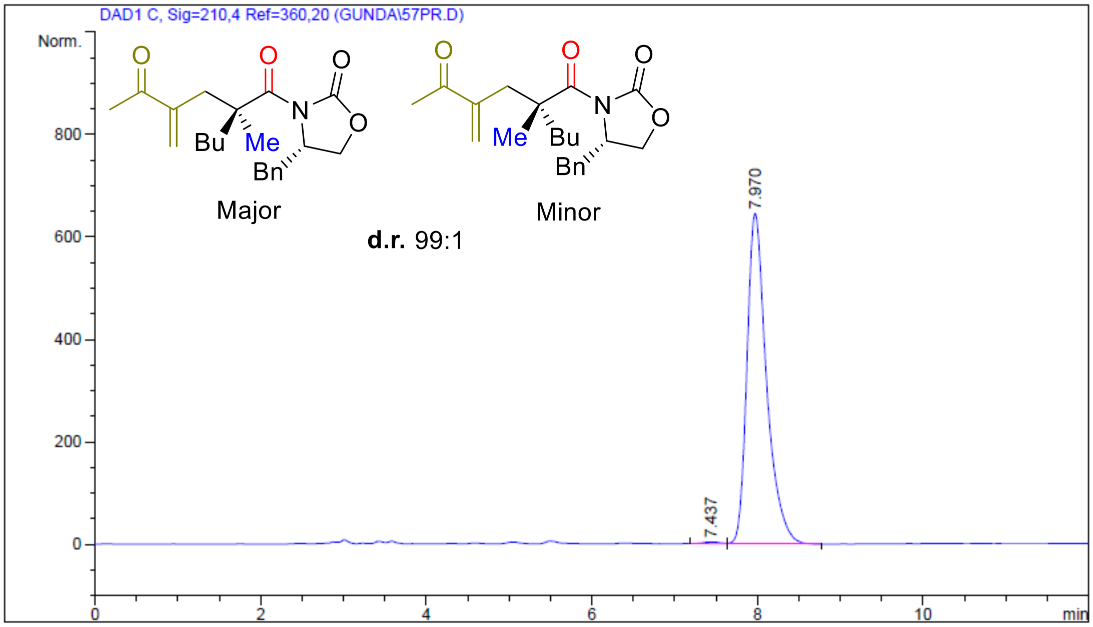

Peak #	RetTime [min]	Type	Width [min]	Area [mAU*s]	Height [mAU]	Area %
1	7.437	BV	0.2174	50.16170	3.49616	0.4711
2	7.970	VP	0.2477	1.05967e4	645.03699	99.5289

Totals : 1.06468e4 648.53315

Compound **4r**

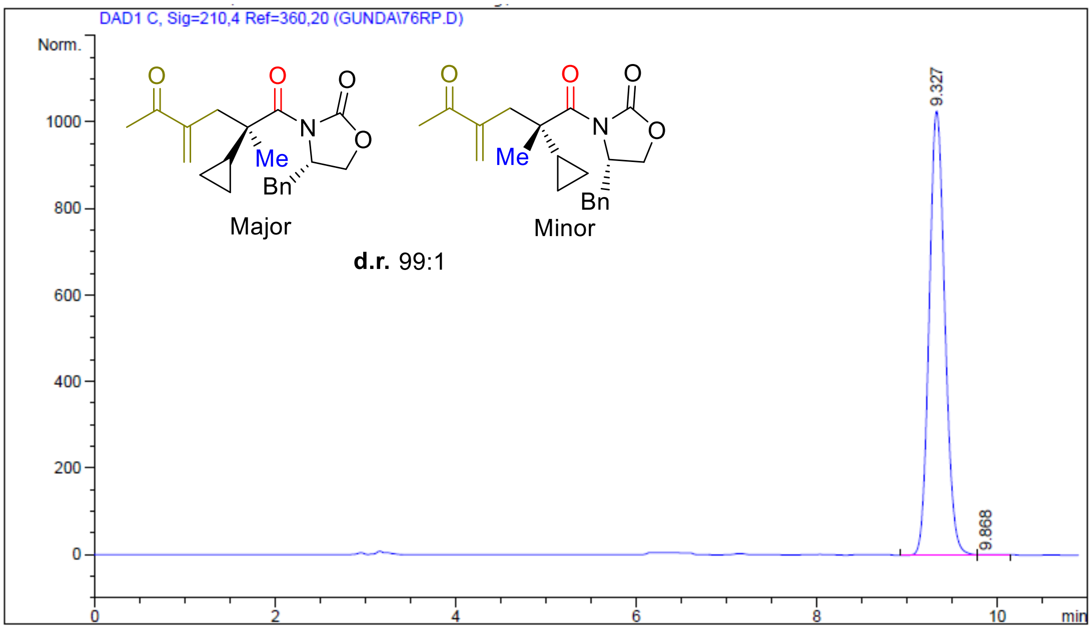

Peak #	RetTime [min]	Type	Width [min]	Area [mAU*s]	Height [mAU]	Area %
1	9.327	VV	0.1892	1.24377e4	1027.41980	99.7790
2	9.868	VB	0.2145	27.54656	1.65240	0.2210

Totals : 1.24653e4 1029.07220

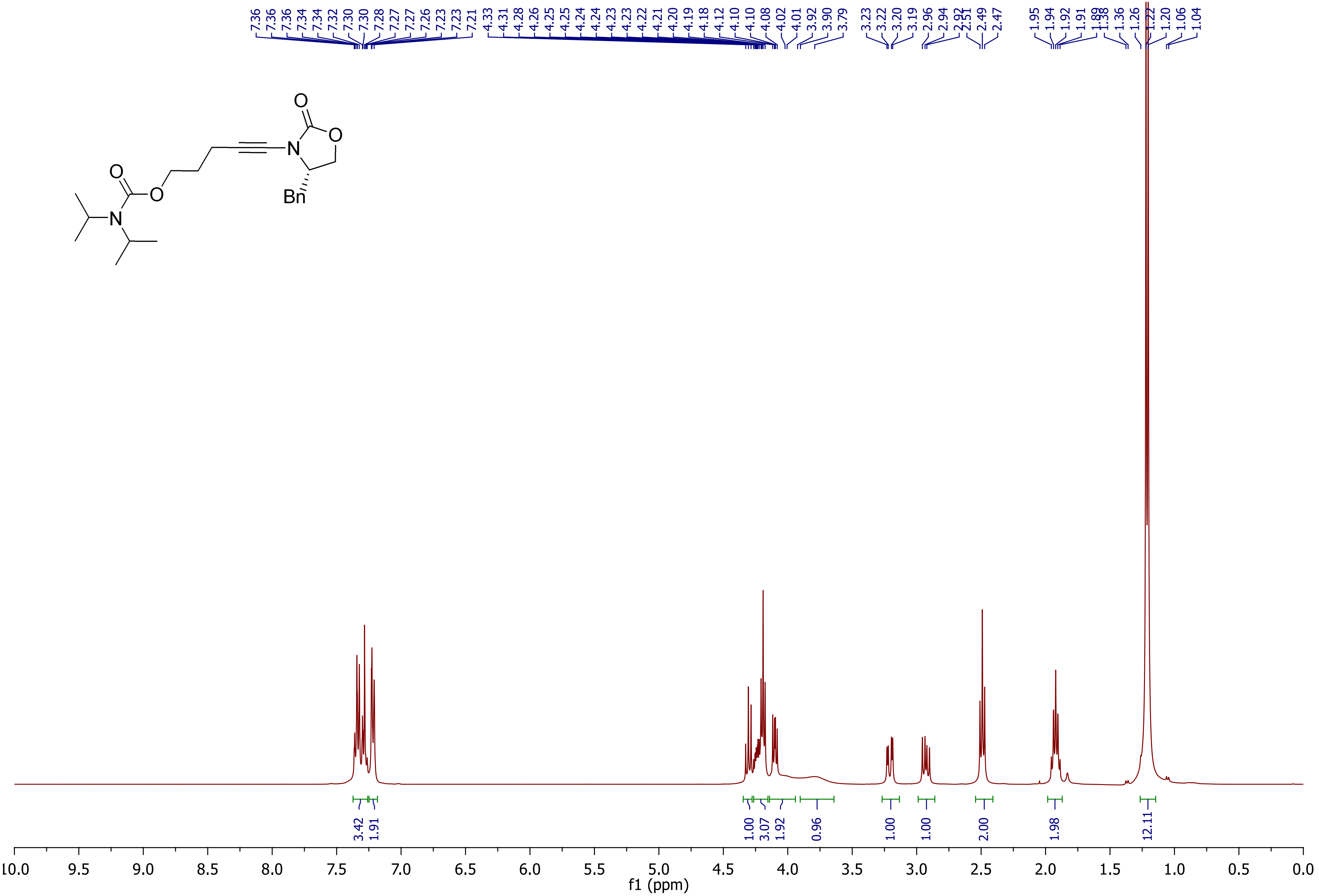
7.36 7.36 7.36 7.34 7.34 7.32 7.30 7.30 7.28 7.27 7.27 7.26 7.23 7.23 7.21
4.33 4.31 4.28 4.26 4.25 4.25 4.24 4.24 4.23 4.23 4.22 4.21 4.20 4.19 4.18 4.12 4.10 4.10 4.08 4.02 4.01 3.92 3.90 3.79
3.23 3.22 3.20 3.19 2.96 2.94 2.92 2.51 2.49 2.47
1.95 1.94 1.92 1.91 1.89 1.38 1.36 1.26 1.22 1.20 1.06 1.04
O
O
N
O
O
N
Bn
3.42 1.91 1.00 3.07 1.92 0.96 1.00 1.00 2.00 1.98 12.11
10.0 9.5 9.0 8.5 8.0 7.5 7.0 6.5 6.0 5.5 5.0 4.5 4.0 3.5 3.0 2.5 2.0 1.5 1.0 0.5 0.0
f1 (ppm)

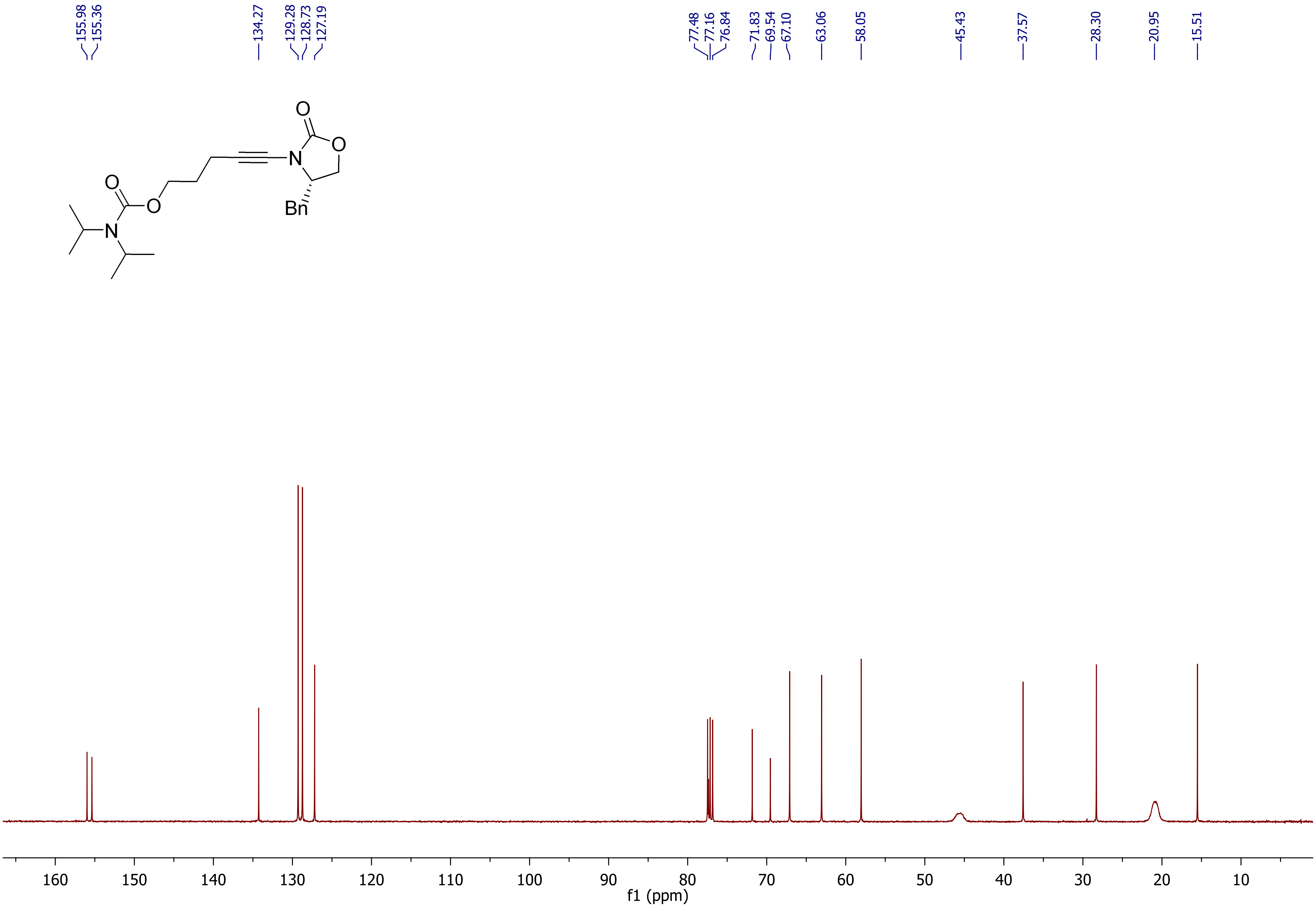

155.98
155.36
134.27
129.28
128.73
127.19
77.48
77.16
76.84
71.83
69.54
67.10
63.06
58.05
45.43
37.57
28.30
20.95
15.51
O
O
N
O
O
N
Bn
160 150 140 130 120 110 100 90 80 70 60 50 40 30 20 10
f1 (ppm)

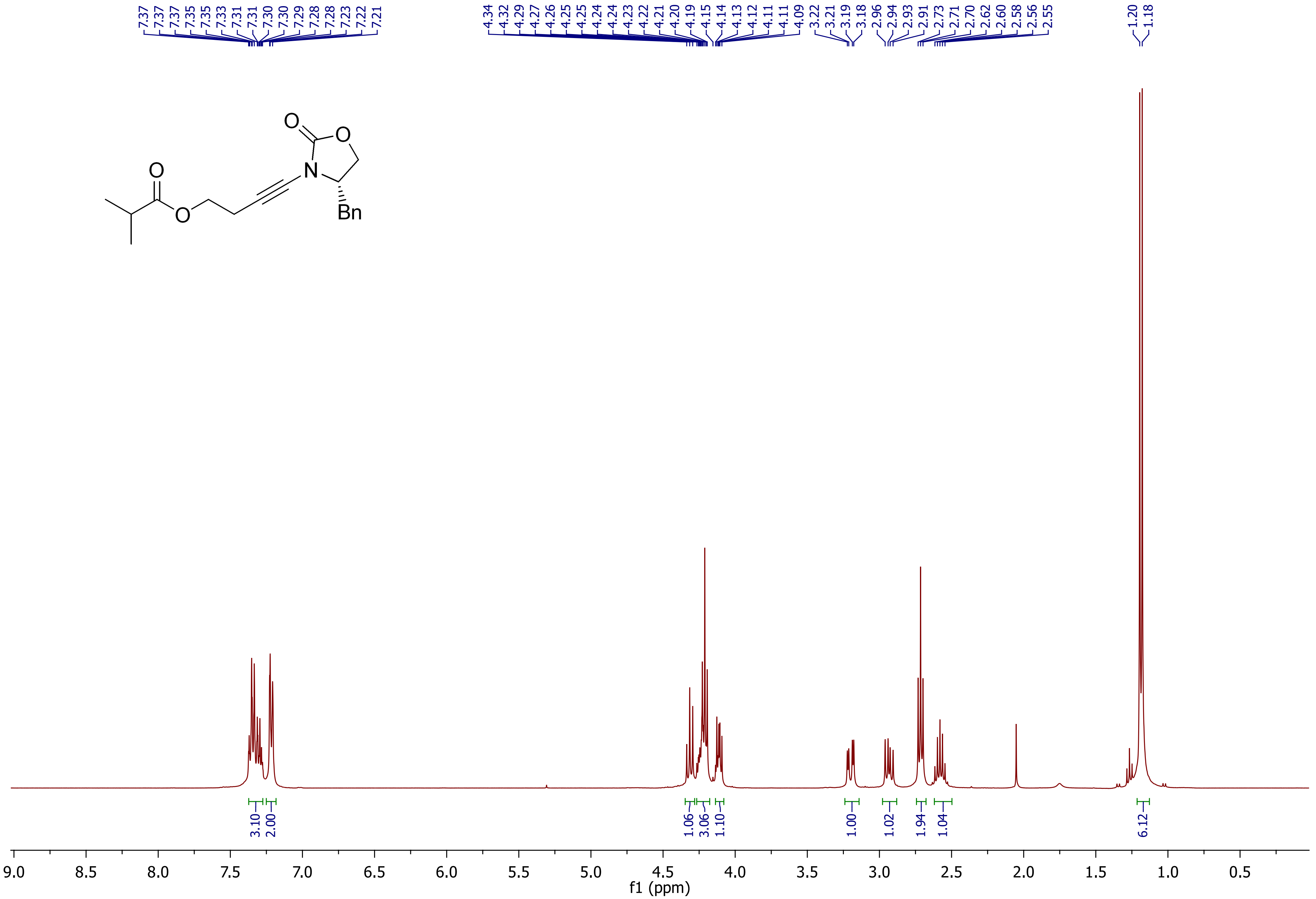

7.37 7.37 7.37 7.35 7.35 7.33 7.31 7.31 7.30 7.30 7.29 7.28 7.28 7.23 7.22 7.21
4.34 4.32 4.29 4.27 4.26 4.25 4.25 4.24 4.24 4.23 4.22 4.21 4.20 4.19 4.15 4.14 4.13 4.12 4.11 4.11 4.09 3.22 3.21 3.19 3.18 2.96 2.94 2.93 2.91 2.73 2.71 2.70 2.62 2.60 2.58 2.56 2.55
1.20 1.18
O
O
N
O
O
Bn
3.10 2.00 1.06 3.06 1.10 1.00 1.02 1.94 1.04 6.12
9.0 8.5 8.0 7.5 7.0 6.5 6.0 5.5 5.0 4.5 4.0 3.5 3.0 2.5 2.0 1.5 1.0 0.5
f1 (ppm)

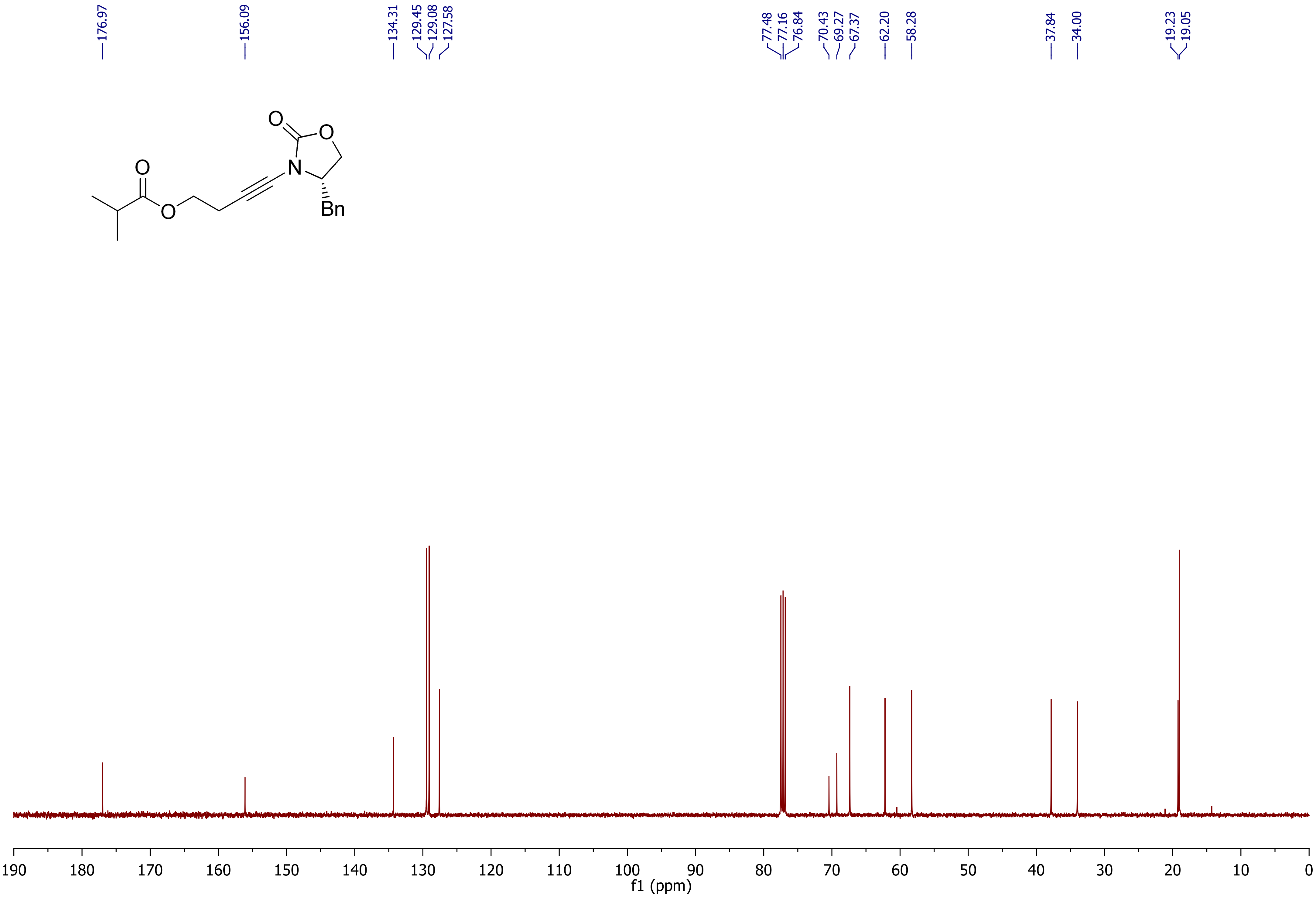
176.97
156.09
134.31
129.45
129.08
127.58
77.48
77.16
76.84
70.43
69.27
67.37
62.20
58.28
37.84
34.00
19.23
19.05
O
O
O
N
O
O
Bn
190
180
170
160
150
140
130
120
110
100
90
80
70
60
50
40
30
20
10
0
f1 (ppm)

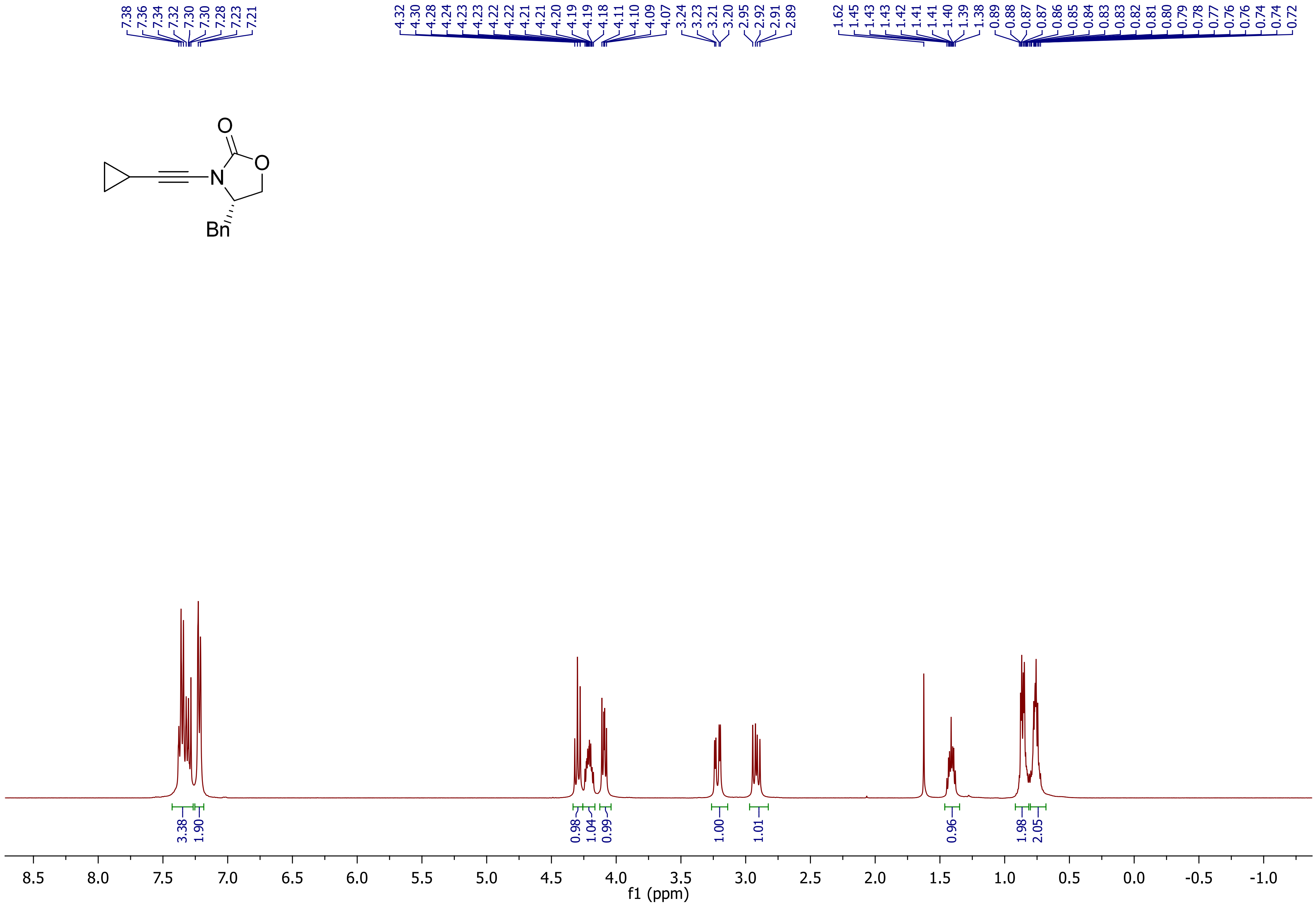
7.38 7.36 7.34 7.32 7.30 7.30 7.28 7.23 7.21
4.32 4.30 4.28 4.24 4.23 4.23 4.22 4.22 4.21 4.21 4.20 4.19 4.19 4.18 4.11 4.10 4.09 4.07 3.24 3.23 3.21 3.20 2.95 2.92 2.91 2.89
1.62 1.45 1.43 1.43 1.42 1.41 1.41 1.40 1.39 1.38 0.89 0.88 0.87 0.87 0.86 0.85 0.84 0.83 0.83 0.82 0.81 0.80 0.79 0.78 0.77 0.76 0.76 0.74 0.74 0.72
O
O
N
Bn
3.38 1.90 0.98 1.04 0.99 1.00 1.01 0.96 1.98 2.05
8.5 8.0 7.5 7.0 6.5 6.0 5.5 5.0 4.5 4.0 3.5 3.0 2.5 2.0 1.5 1.0 0.5 0.0 -0.5 -1.0
f1 (ppm)

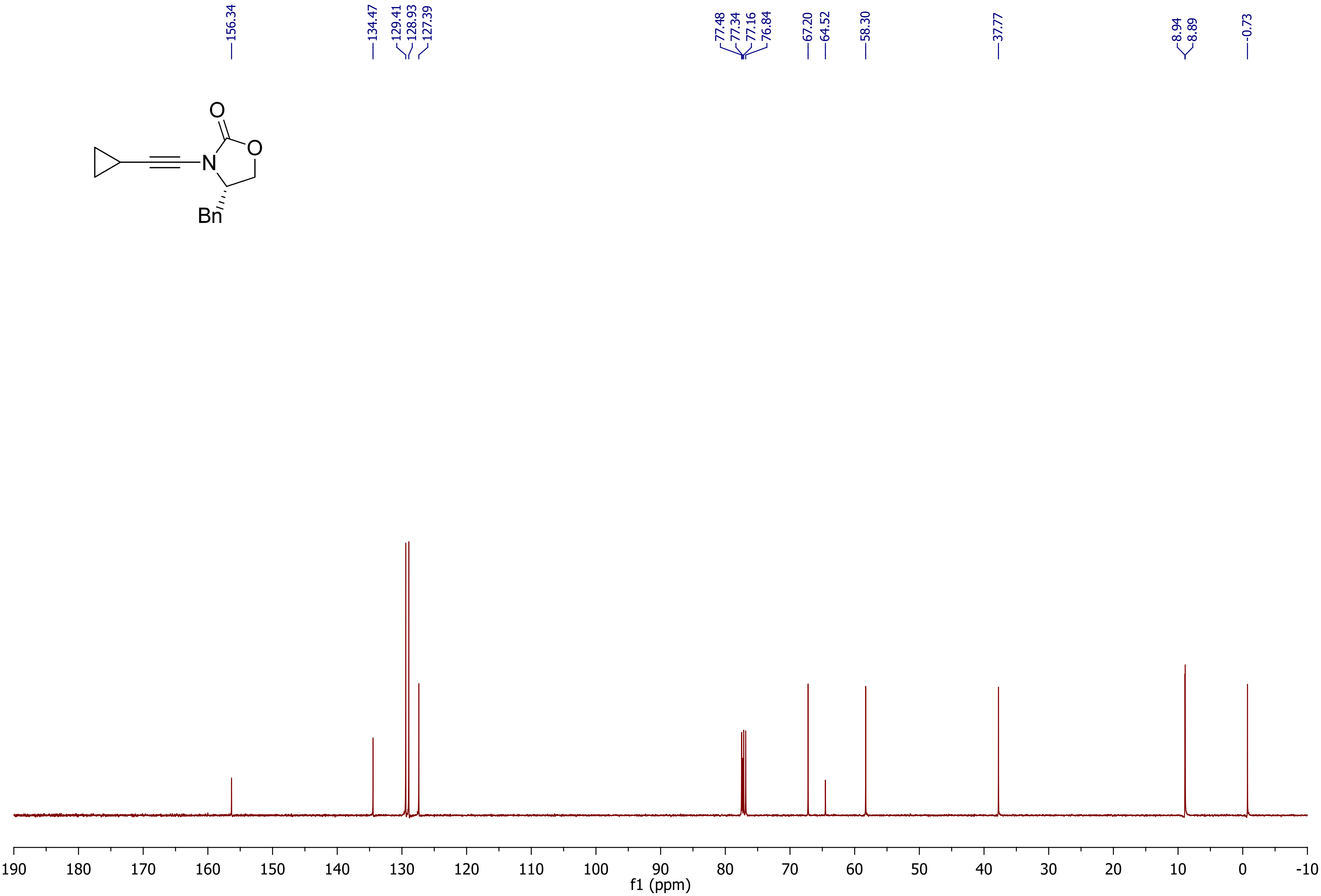
156.34
134.47
129.41
128.93
127.39
77.48
77.34
77.16
76.84
67.20
64.52
58.30
37.77
8.94
8.89
-0.73
O
N
O
Bn
190 180 170 160 150 140 130 120 110 100 90 80 70 60 50 40 30 20 10 0 -10
f1 (ppm)

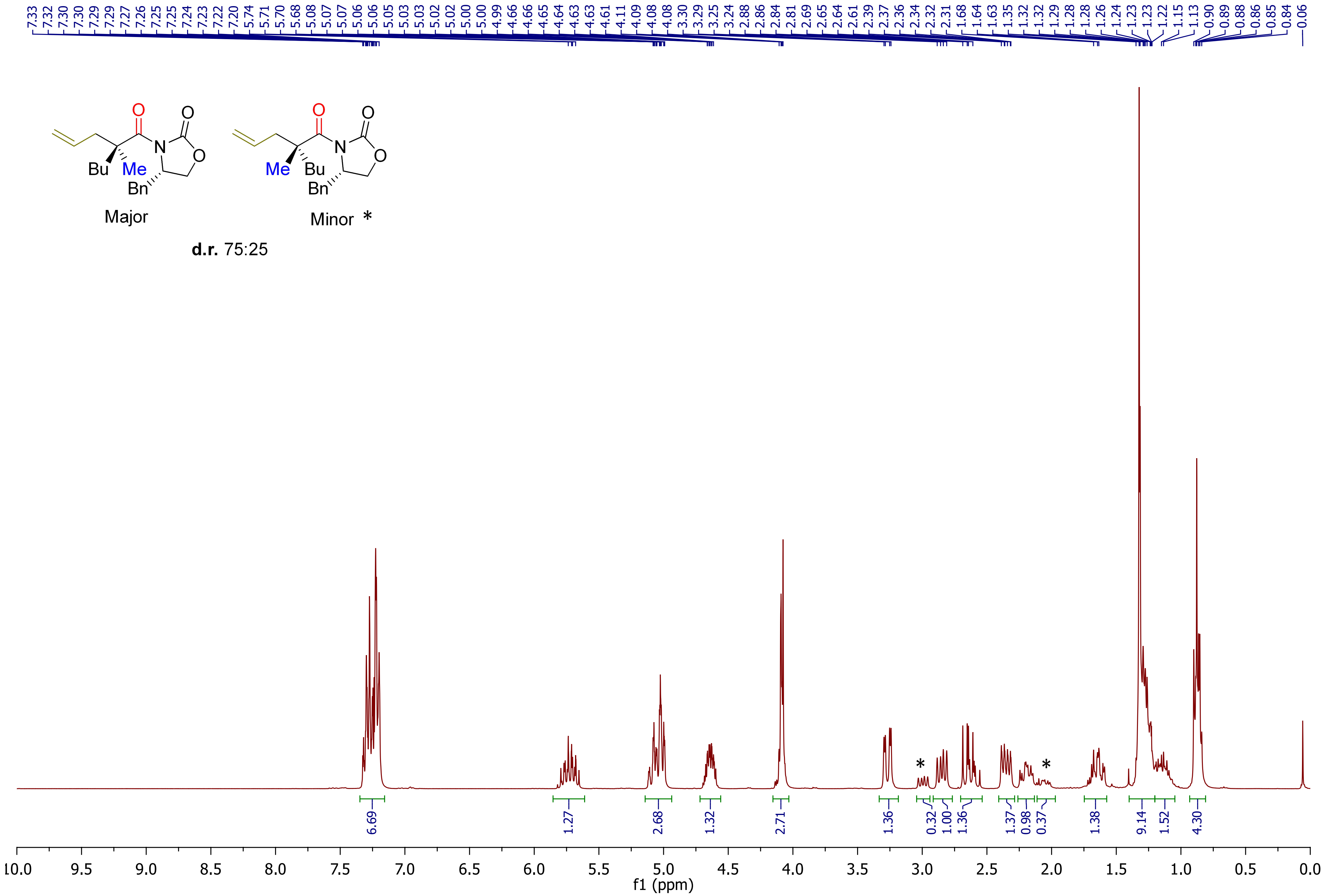
Major
Minor *
d.r. 75:25
f1 (ppm)

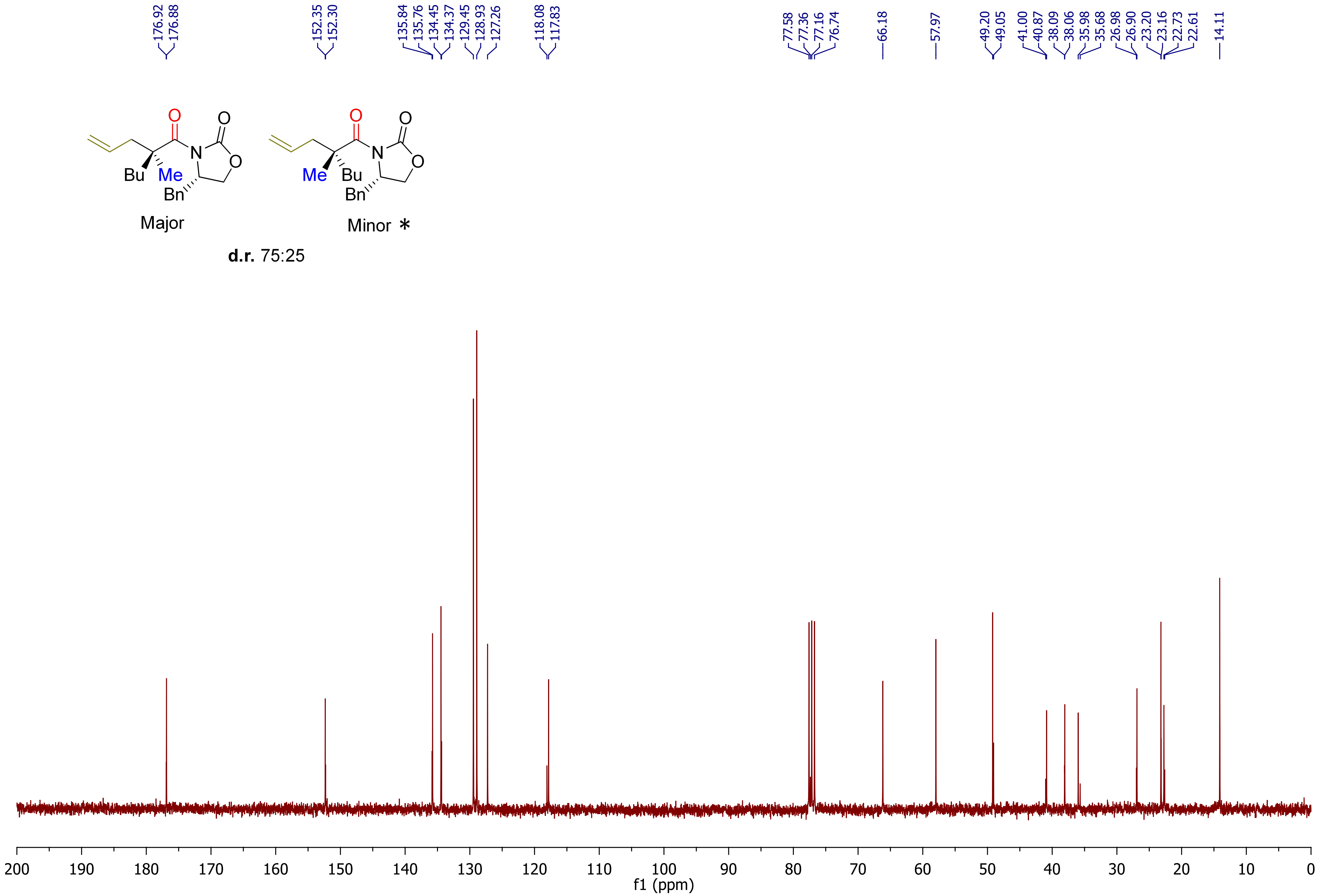
176.92
176.88
152.35
152.30
135.84
135.76
134.45
134.37
129.45
128.93
127.26
118.08
117.83
77.58
77.36
77.16
76.74
66.18
57.97
49.20
49.05
41.00
40.87
38.09
38.06
35.98
35.68
26.98
26.90
23.20
23.16
22.73
22.61
14.11
Major
Minor *
d.r. 75:25
f1 (ppm)

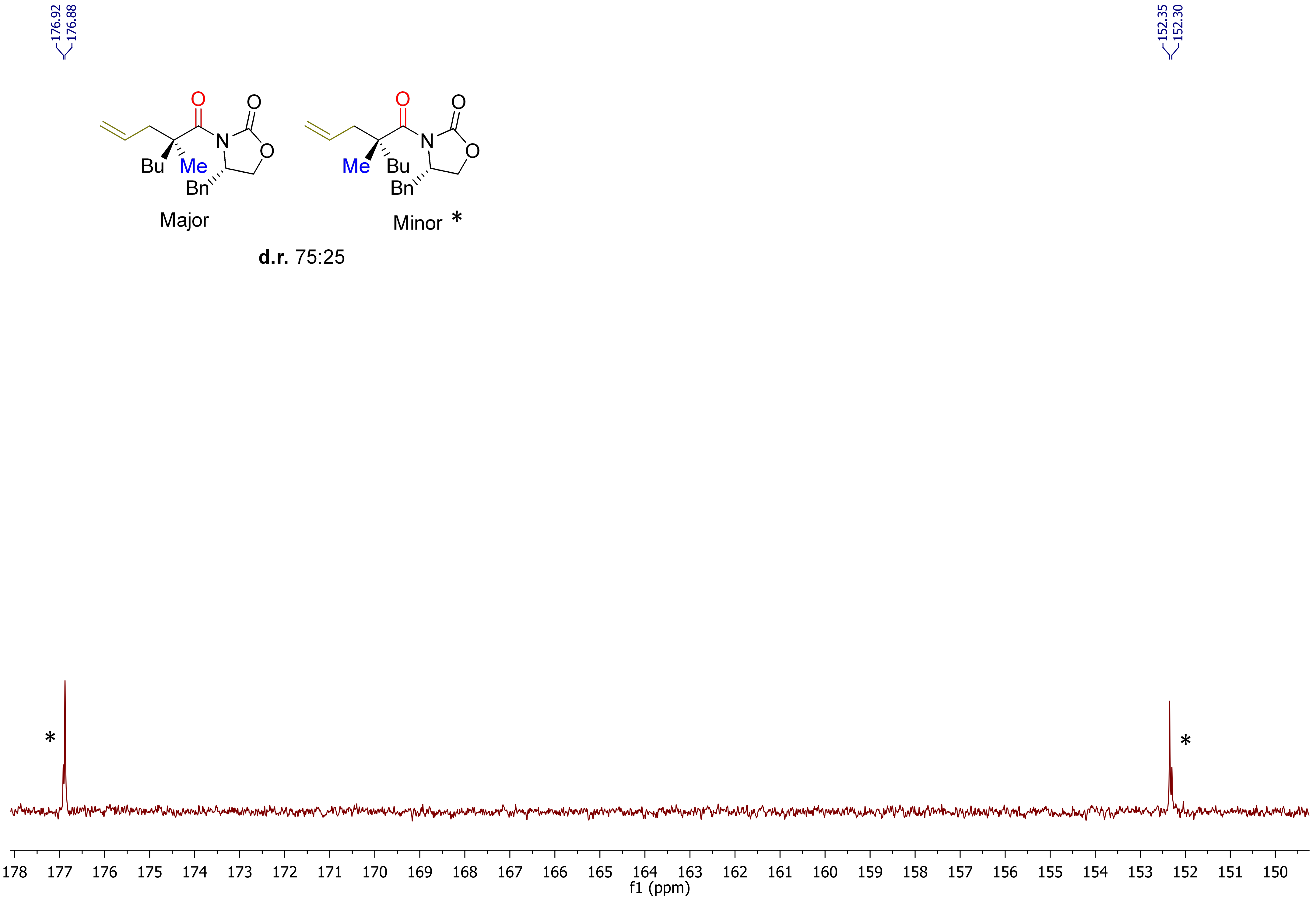
176.92
176.88
152.35
152.30
O
O
N
O
Bu
Me
Bn
Major
Minor *
d.r. 75:25
*
*
178 177 176 175 174 173 172 171 170 169 168 167 166 165 164 163 162 161 160 159 158 157 156 155 154 153 152 151 150
f1 (ppm)

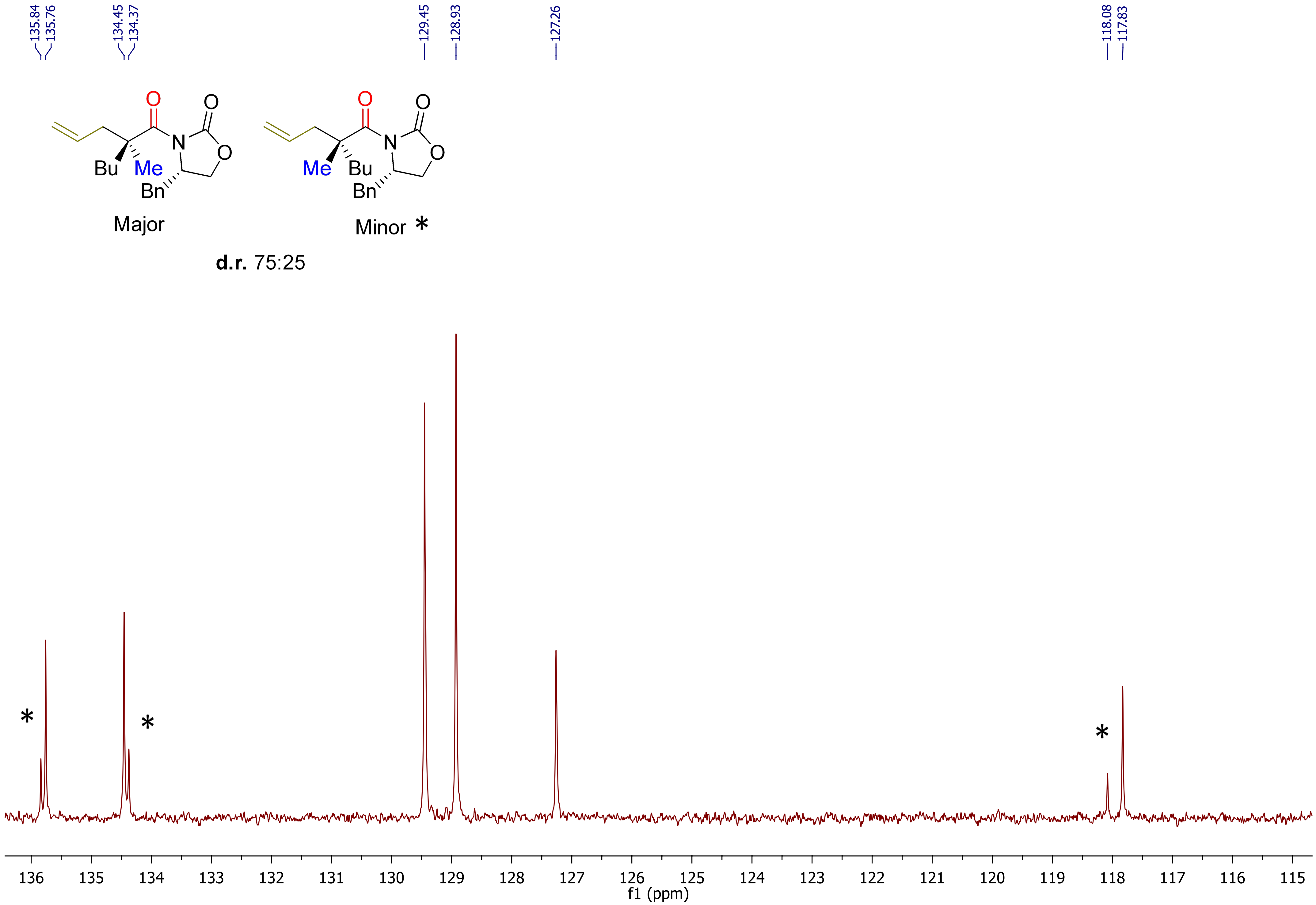
135.84
135.76
134.45
134.37
129.45
128.93
127.26
118.08
117.83
O
O
N
O
Bu
Me
Bn
Major
O
O
N
O
Me
Bu
Bn
Minor *
d.r. 75:25
*
*
*
136
135
134
133
132
131
130
129
128
127
126
125
124
123
122
121
120
119
118
117
116
115
f1 (ppm)

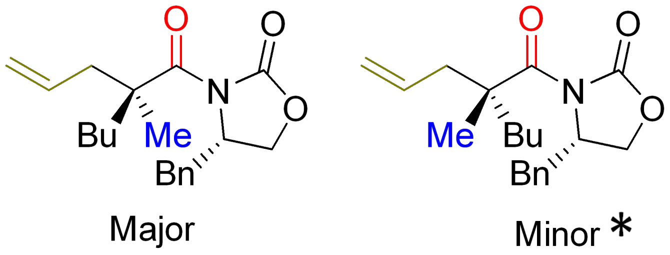

Bu
Me
Bn
Major
Me
Bu
Bn
Minor *

d.r. 75:25

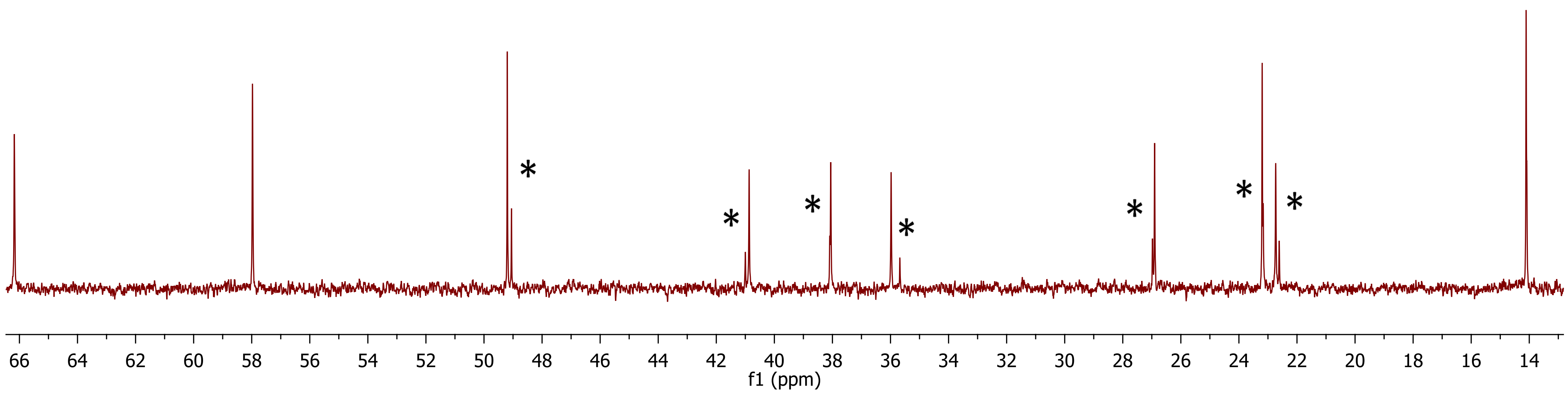

66.18
57.97
49.20
49.05
41.00
40.87
38.09
38.06
35.98
35.68
26.98
26.90
23.20
23.16
22.73
22.61
14.11
f1 (ppm)

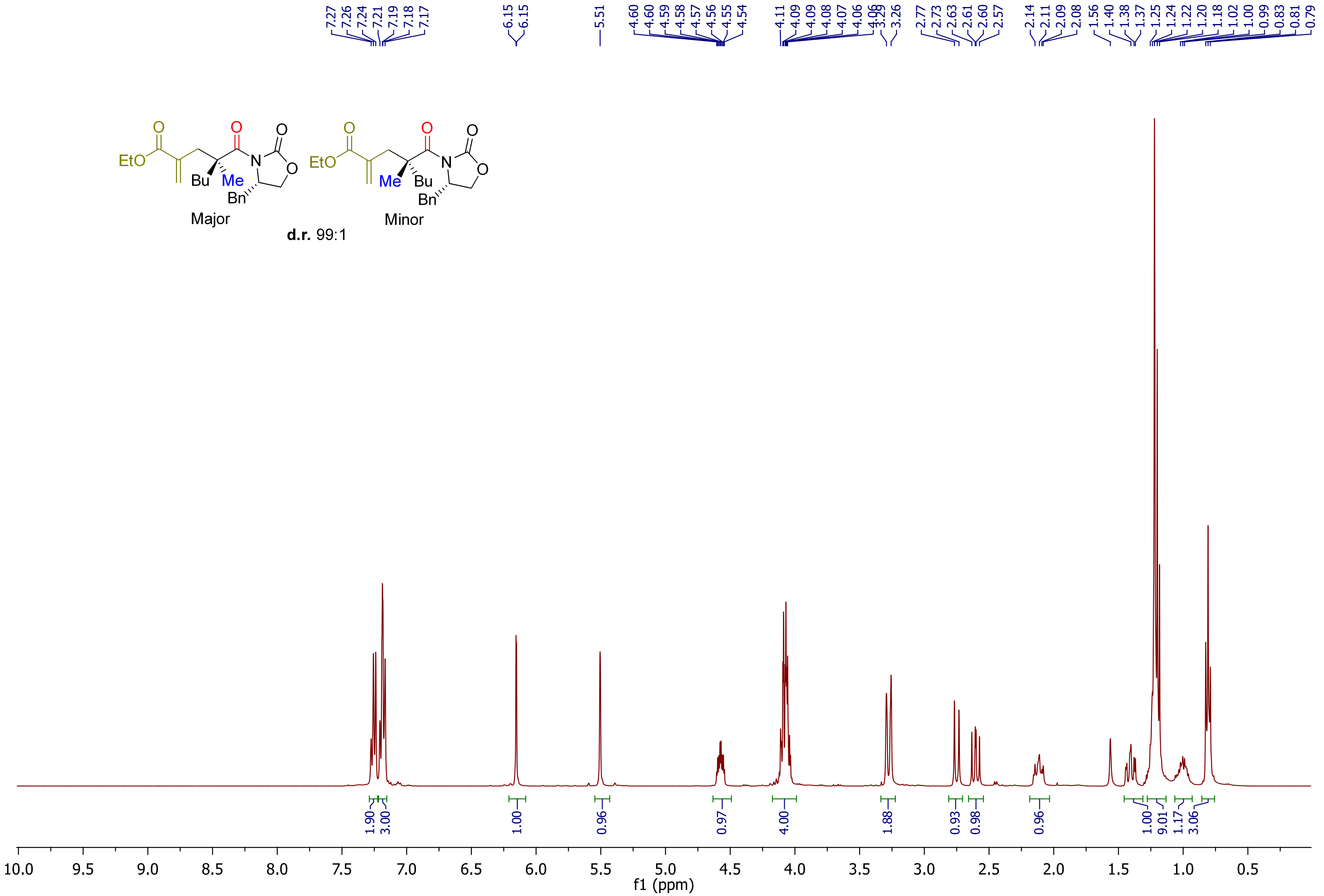
EtO
O
O
N
O
O
Bu
Me
Bn
Major
d.r. 99:1
EtO
O
O
N
O
O
Me
Bu
Bn
Minor
7.27 7.26 7.24 7.21 7.19 7.18 7.17
6.15 6.15
5.51
4.60 4.60 4.59 4.58 4.57 4.56 4.55 4.54
4.11 4.09 4.09 4.08 4.07 4.06
3.29 3.26
2.77 2.73 2.63 2.61 2.60 2.57
2.14 2.11 2.09 2.08
1.56 1.40 1.38 1.37 1.25 1.24 1.22 1.20 1.18 1.02 1.00 0.99 0.83 0.81 0.79
1.90 3.00 1.00 0.96 0.97 4.00 1.88 0.93 0.98 0.96 1.00 9.01 1.17 3.06
10.0 9.5 9.0 8.5 8.0 7.5 7.0 6.5 6.0 5.5 5.0 4.5 4.0 3.5 3.0 2.5 2.0 1.5 1.0 0.5
f1 (ppm)

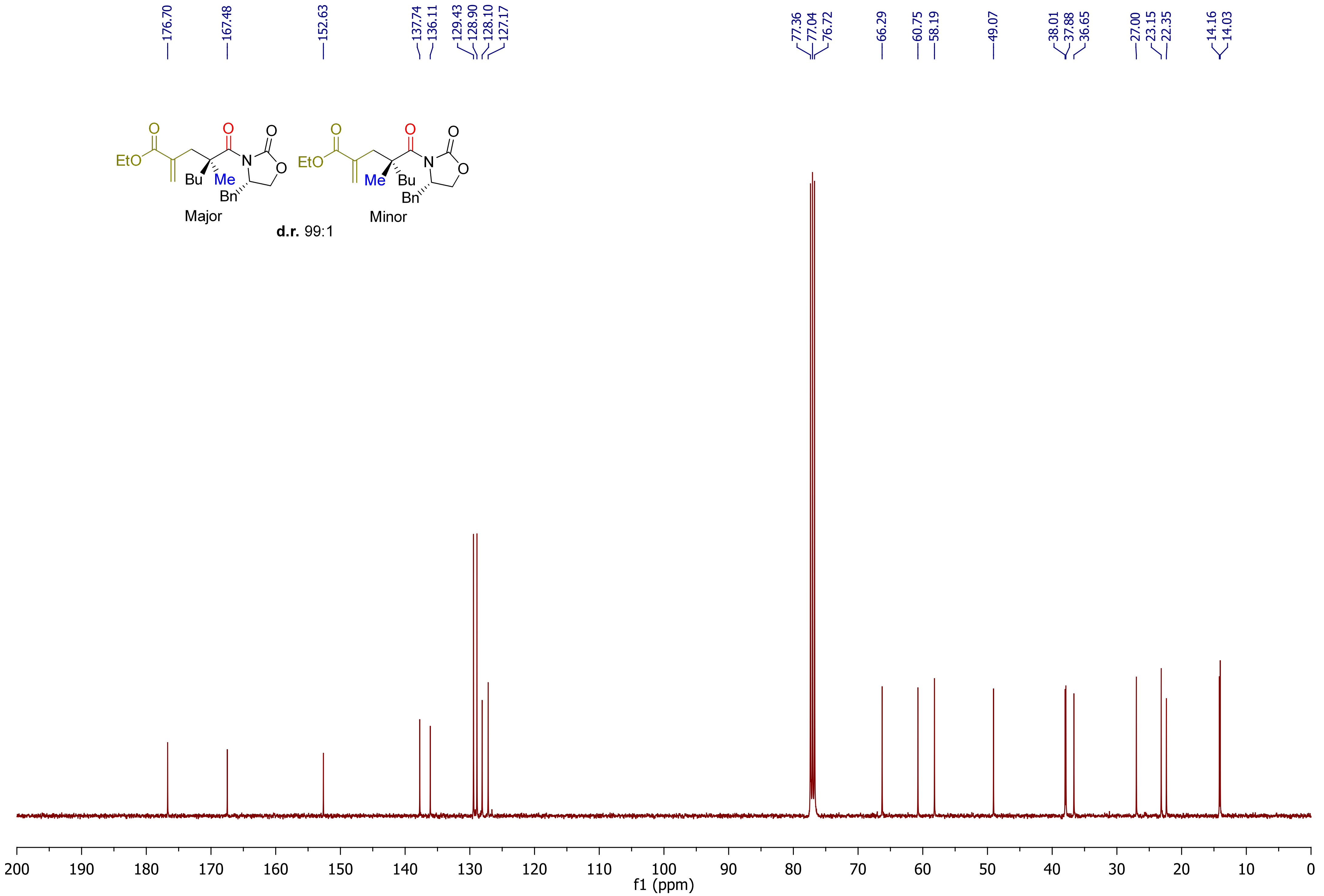

176.70
167.48
152.63
137.74
136.11
129.43
128.90
128.10
127.17
77.36
77.04
76.72
66.29
60.75
58.19
49.07
38.01
37.88
36.65
27.00
23.15
22.35
14.16
14.03
EtO
Bu
Me
Bn
Major
d.r. 99:1
Minor
f1 (ppm)

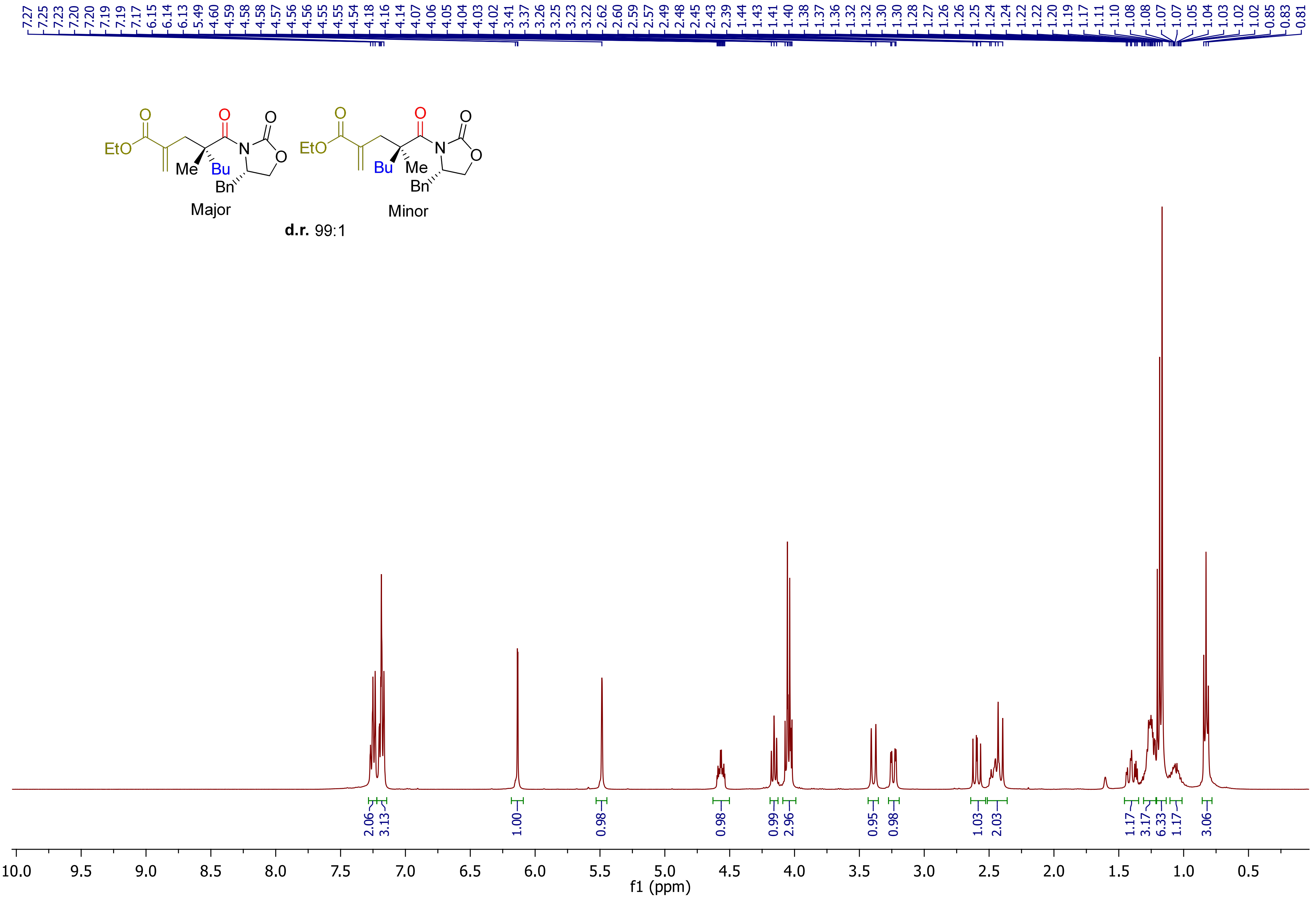
EtO
Me
Bu
Bn
Major
EtO
Bu
Me
Bn
Minor
d.r. 99:1
2.06
3.13
1.00
0.98
0.98
0.99
2.96
0.95
0.98
1.03
2.03
1.17
3.17
6.33
1.17
3.06
f1 (ppm)

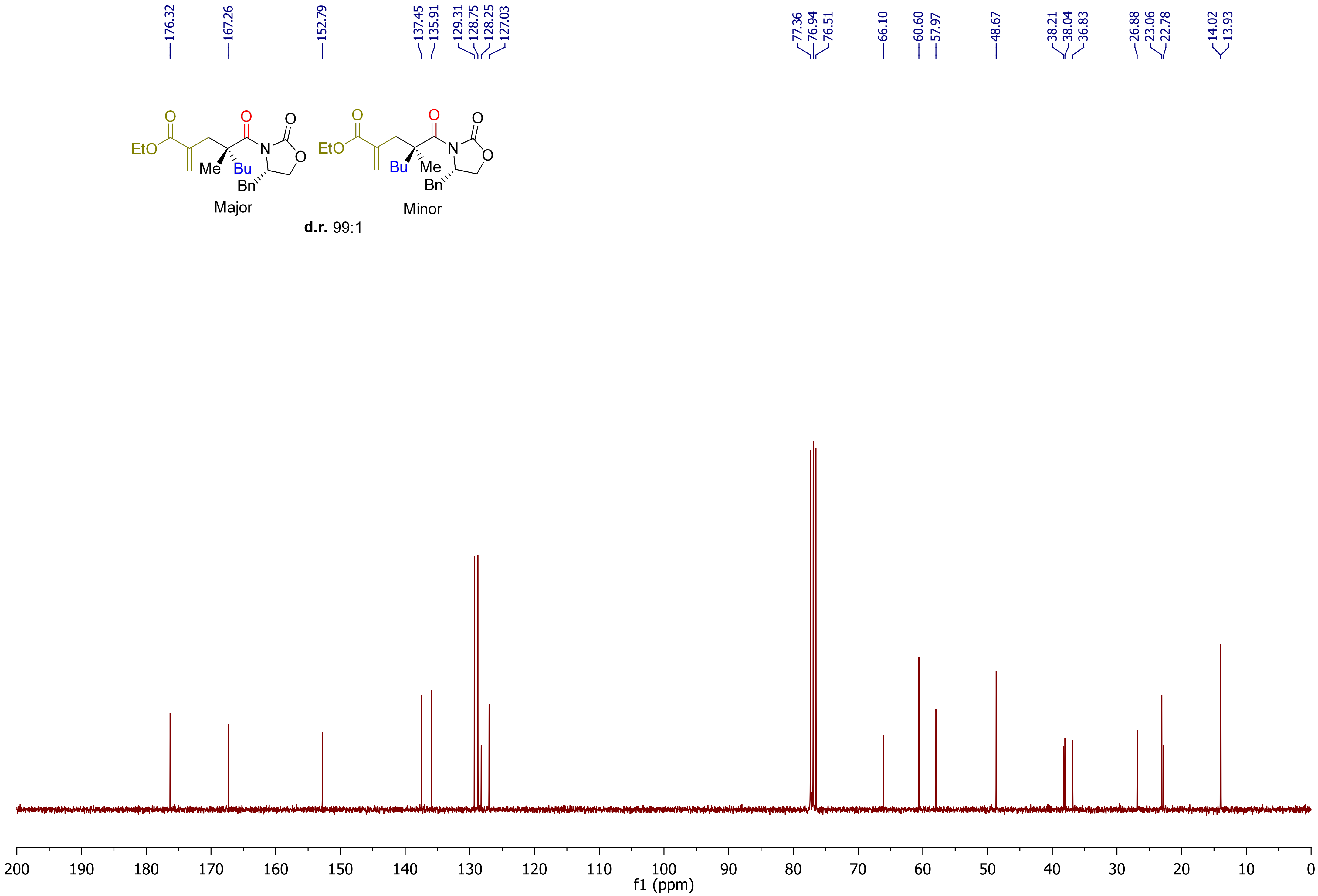
176.32
167.26
152.79
137.45
135.91
129.31
128.75
128.25
127.03
77.36
76.94
76.51
66.10
60.60
57.97
48.67
38.21
38.04
36.83
26.88
23.06
22.78
14.02
13.93
EtO
O
O
O
N
O
Me
Bu
Bn
Major
d.r. 99:1
Minor
200
190
180
170
160
150
140
130
120
110
100
90
80
70
60
50
40
30
20
10
0
f1 (ppm)

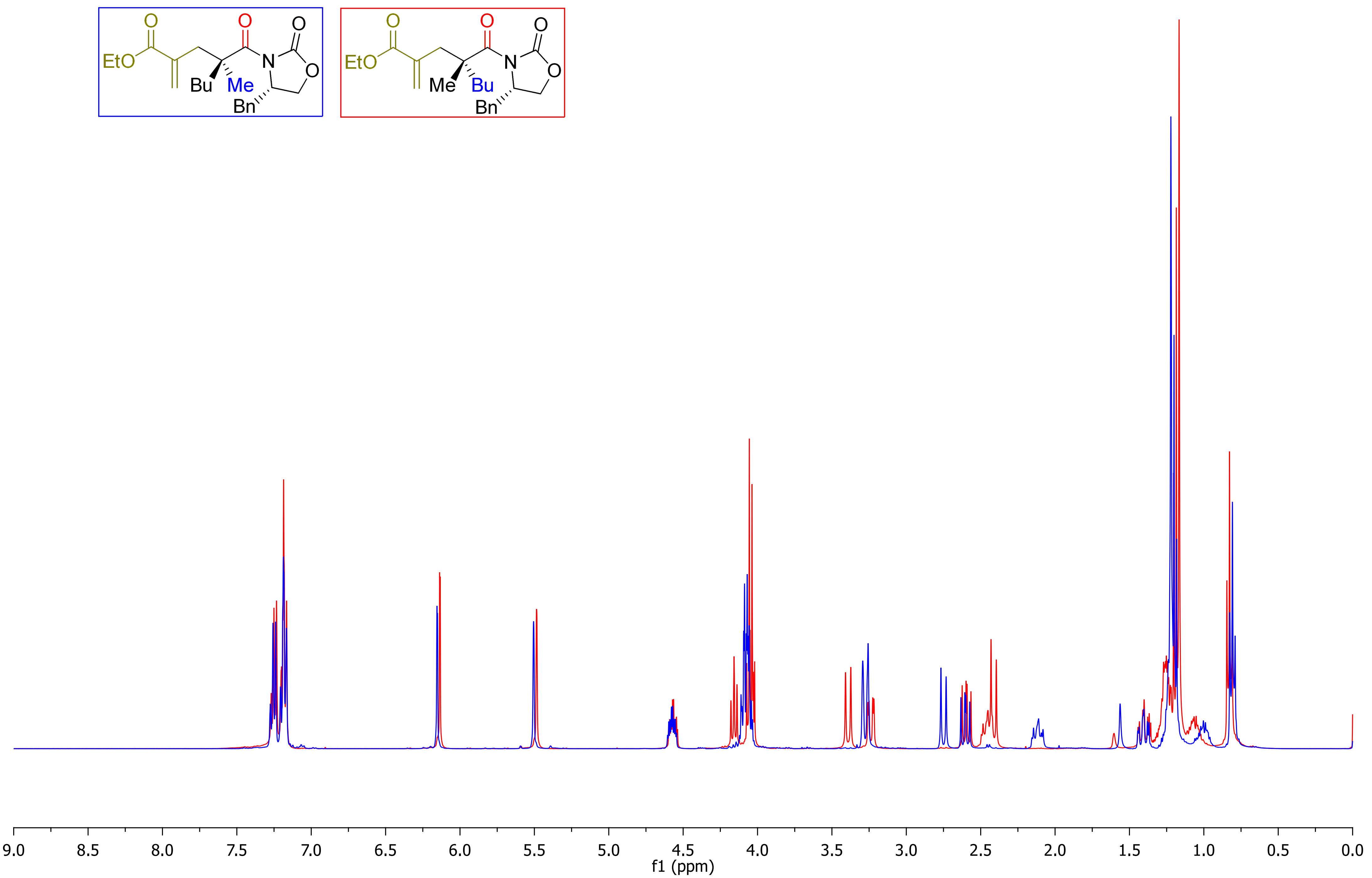
EtO
O
O
O
N
O
Bu
Me
Bn
EtO
O
O
O
N
O
Me
Bu
Bn
9.0
8.5
8.0
7.5
7.0
6.5
6.0
5.5
5.0
4.5
4.0
3.5
3.0
2.5
2.0
1.5
1.0
0.5
0.0
f1 (ppm)

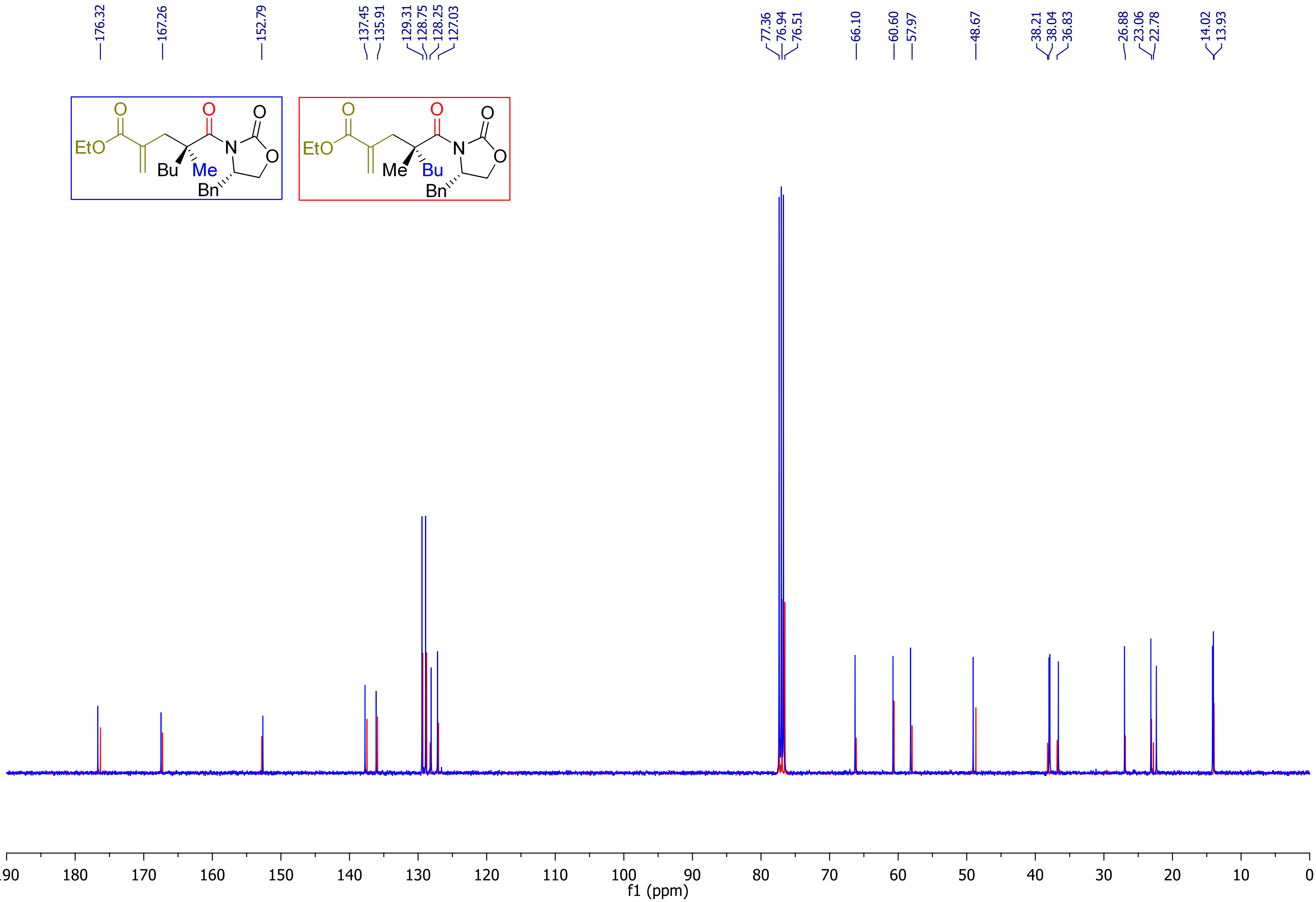

176.32
167.26
152.79
137.45
135.91
129.31
128.75
128.25
127.03
77.36
76.94
76.51
66.10
60.60
57.97
48.67
38.21
38.04
36.83
26.88
23.06
22.78
14.02
13.93
EtO
O
N
Bu
Me
Bn
190 180 170 160 150 140 130 120 110 100 90 80 70 60 50 40 30 20 10 0
f1 (ppm)

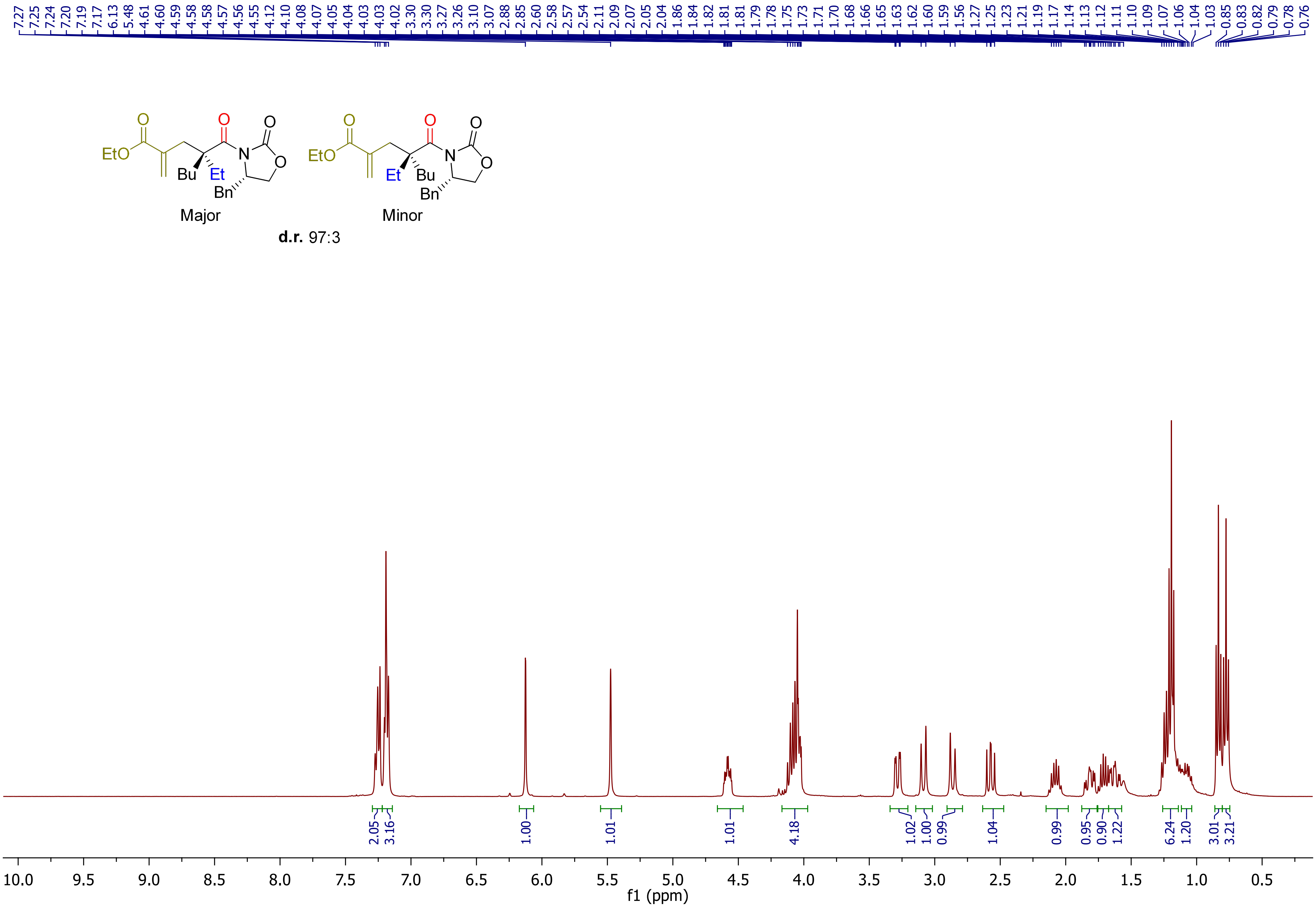

EtO
Bu
Et
Bn
Major
EtO
Et
Bu
Bn
Minor
d.r. 97:3
f1 (ppm)

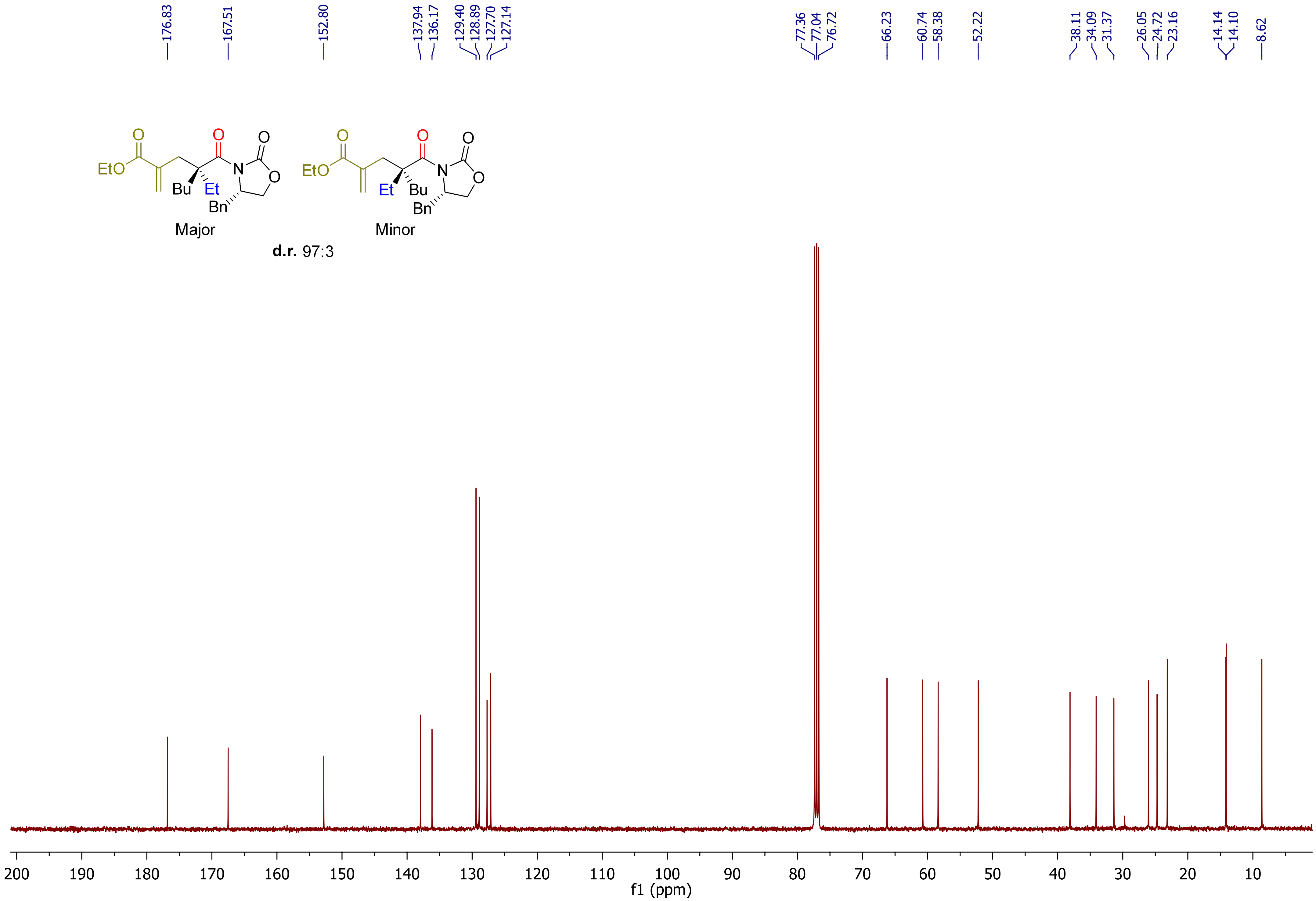
176.83
167.51
152.80
137.94
136.17
129.40
128.89
127.70
127.14
77.36
77.04
76.72
66.23
60.74
58.38
52.22
38.11
34.09
31.37
26.05
24.72
23.16
14.14
14.10
8.62
EtO
O
O
O
N
O
Bu
Et
Bn
Major
EtO
O
O
O
N
O
Et
Bu
Bn
Minor
d.r. 97:3
200
190
180
170
160
150
140
130
120
110
100
90
80
70
60
50
40
30
20
10
f1 (ppm)

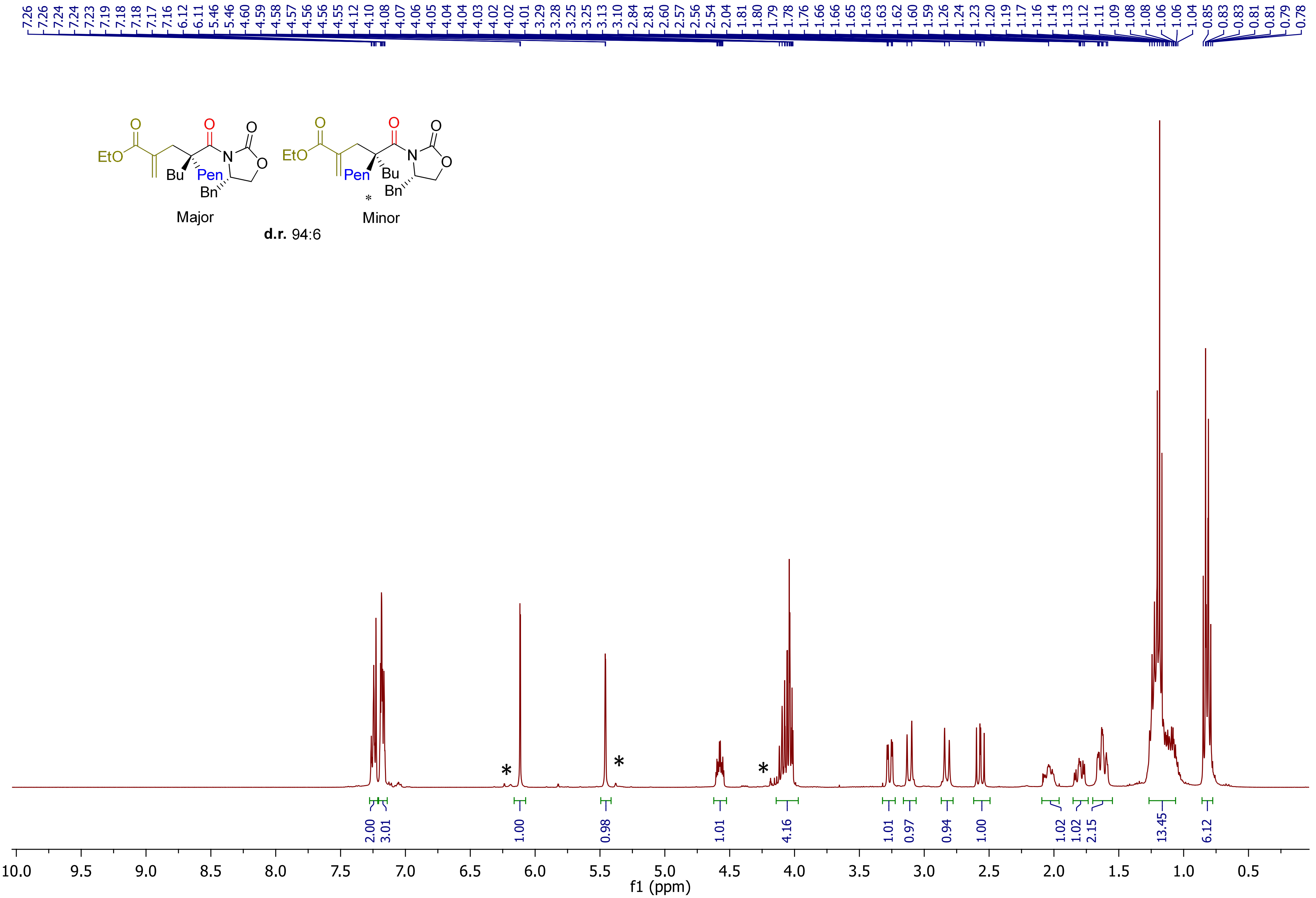
EtO
Bu
Pen
Bn
N
O
Major
Minor
d.r. 94:6
f1 (ppm)

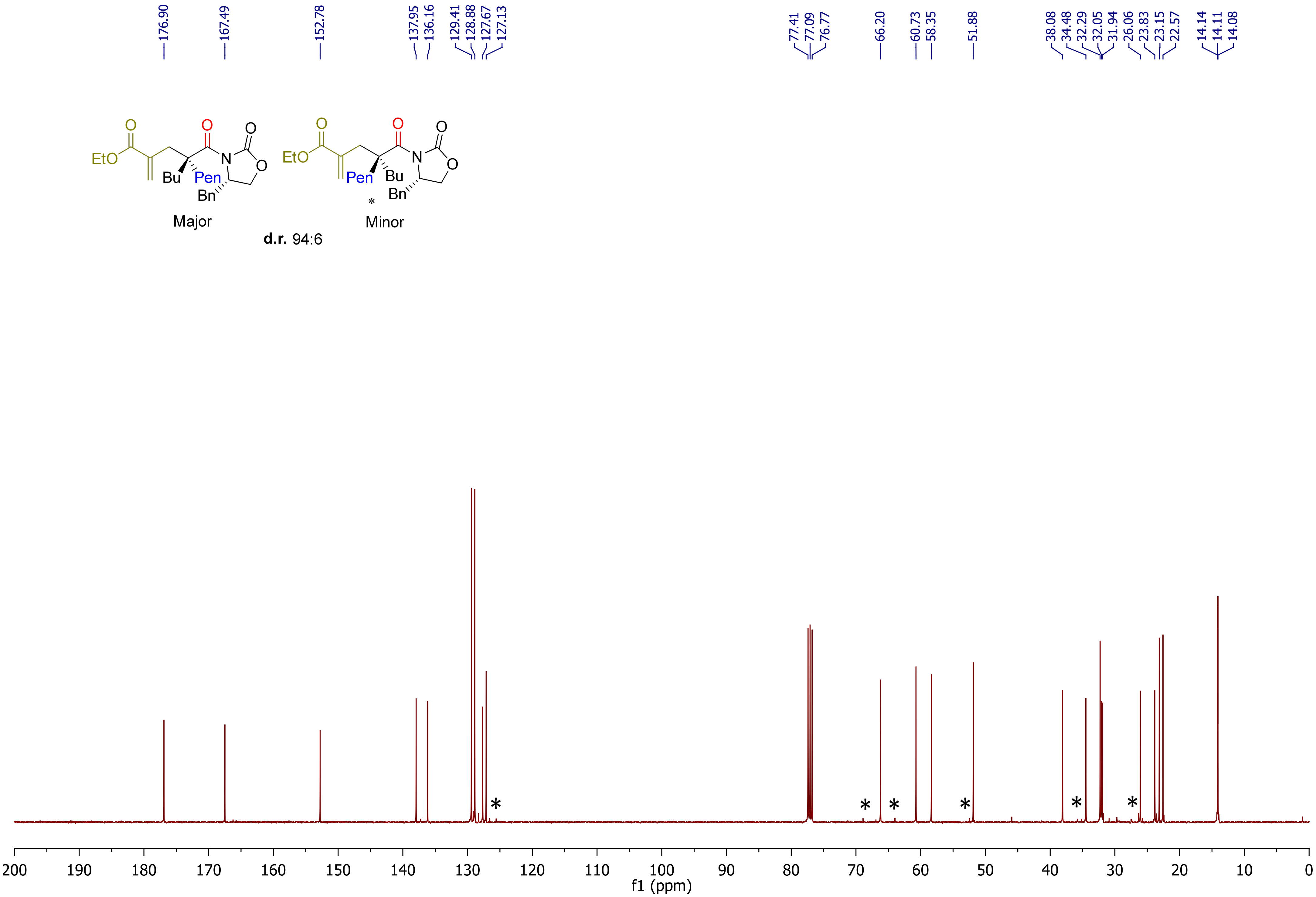
176.90
167.49
152.78
137.95
136.16
129.41
128.88
127.67
127.13
77.41
77.09
76.77
66.20
60.73
58.35
51.88
38.08
34.48
32.29
32.05
31.94
26.06
23.83
23.15
22.57
14.14
14.11
14.08
EtO
O
O
N
O
O
Bu
Pen
Bn
Major
Minor
d.r. 94:6
f1 (ppm)
200
190
180
170
160
150
140
130
120
110
100
90
80
70
60
50
40
30
20
10
0

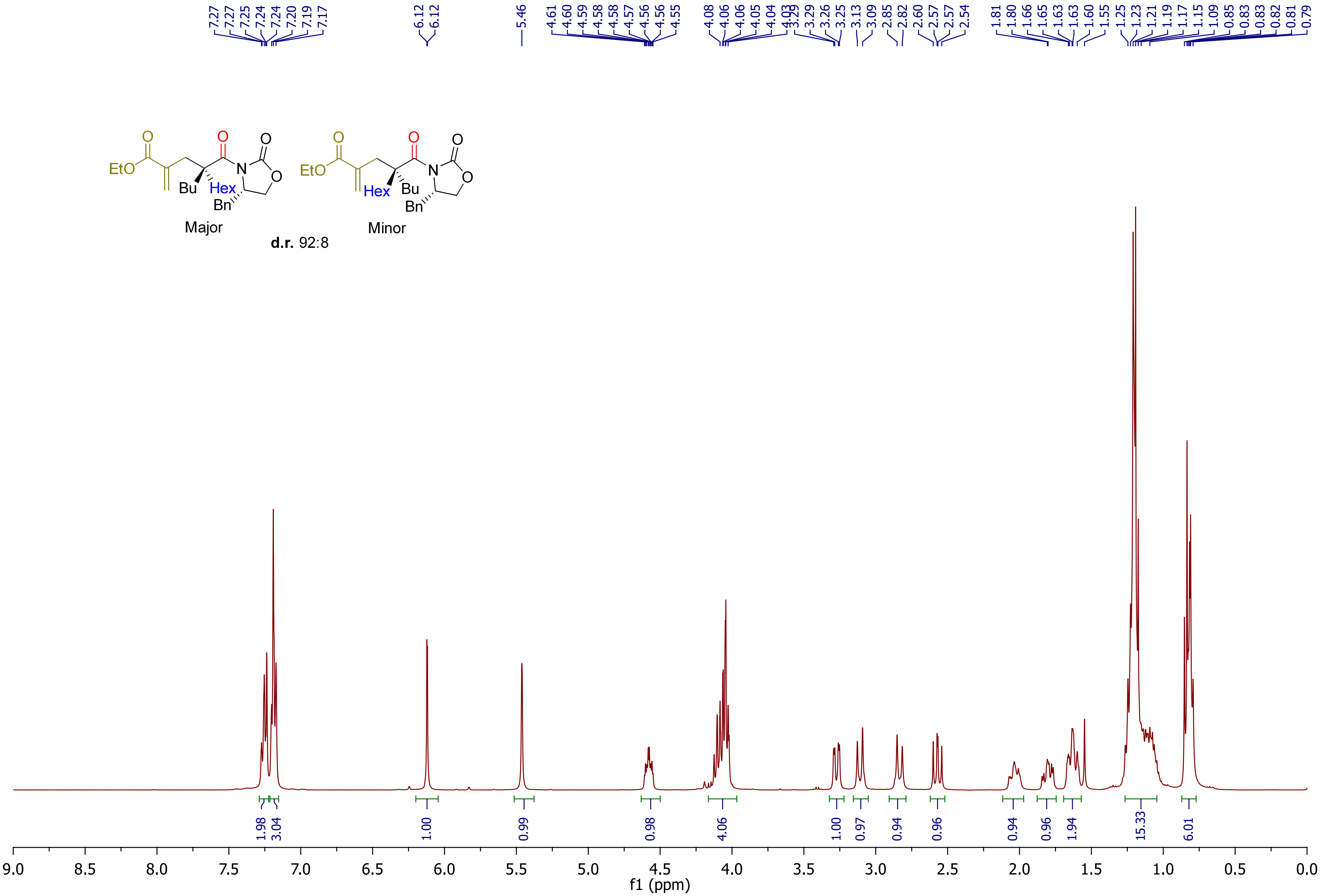
7.27 7.27 7.25 7.24 7.24 7.20 7.19 7.17
6.12 6.12
5.46
4.61 4.60 4.59 4.58 4.58 4.57 4.56 4.56 4.55
4.08 4.06 4.06 4.05 4.04 4.03
3.29 3.29 3.26 3.25 3.13 3.09 2.85 2.82 2.60 2.57 2.57 2.54
1.81 1.80 1.66 1.65 1.63 1.63 1.60 1.55 1.25 1.23 1.21 1.19 1.17 1.15 1.09 0.85 0.83 0.83 0.82 0.81 0.79
EtO
Bu
Hex
Bn
Major
d.r. 92:8
Minor
1.98 3.04 1.00 0.99 0.98 4.06 1.00 0.97 0.94 0.96 0.94 0.96 1.94 15.33 6.01
9.0 8.5 8.0 7.5 7.0 6.5 6.0 5.5 5.0 4.5 4.0 3.5 3.0 2.5 2.0 1.5 1.0 0.5 0.0
f1 (ppm)

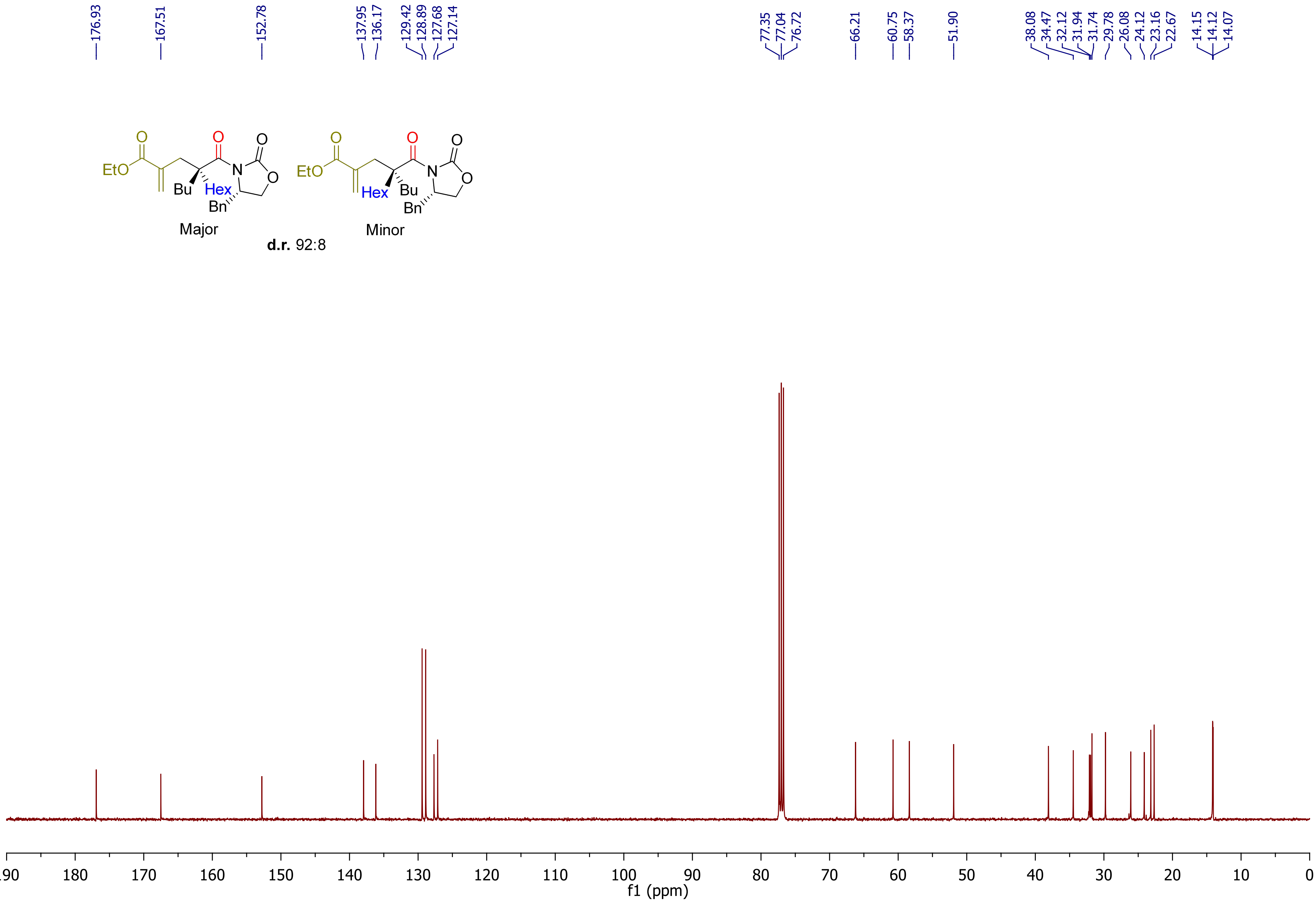

176.93
167.51
152.78
137.95
136.17
129.42
128.89
127.68
127.14
77.35
77.04
76.72
66.21
60.75
58.37
51.90
38.08
34.47
32.12
31.94
31.74
29.78
26.08
24.12
23.16
22.67
14.15
14.12
14.07
EtO
Bu
Hex
Bn
Major
d.r. 92:8
Minor
190 180 170 160 150 140 130 120 110 100 90 80 70 60 50 40 30 20 10 0
f1 (ppm)

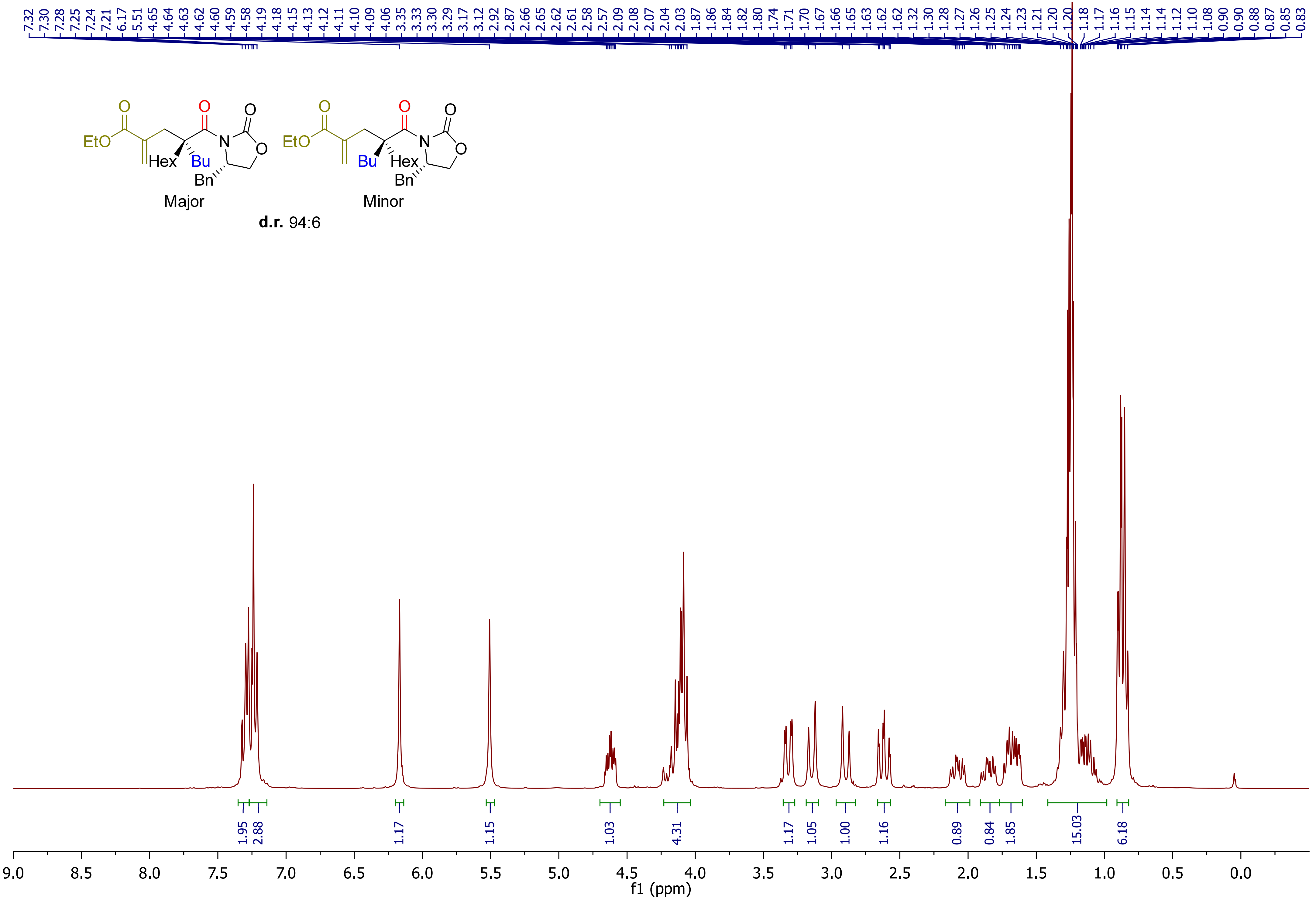
EtO
Hex
Bu
Bn
Major
Minor
d.r. 94:6
f1 (ppm)

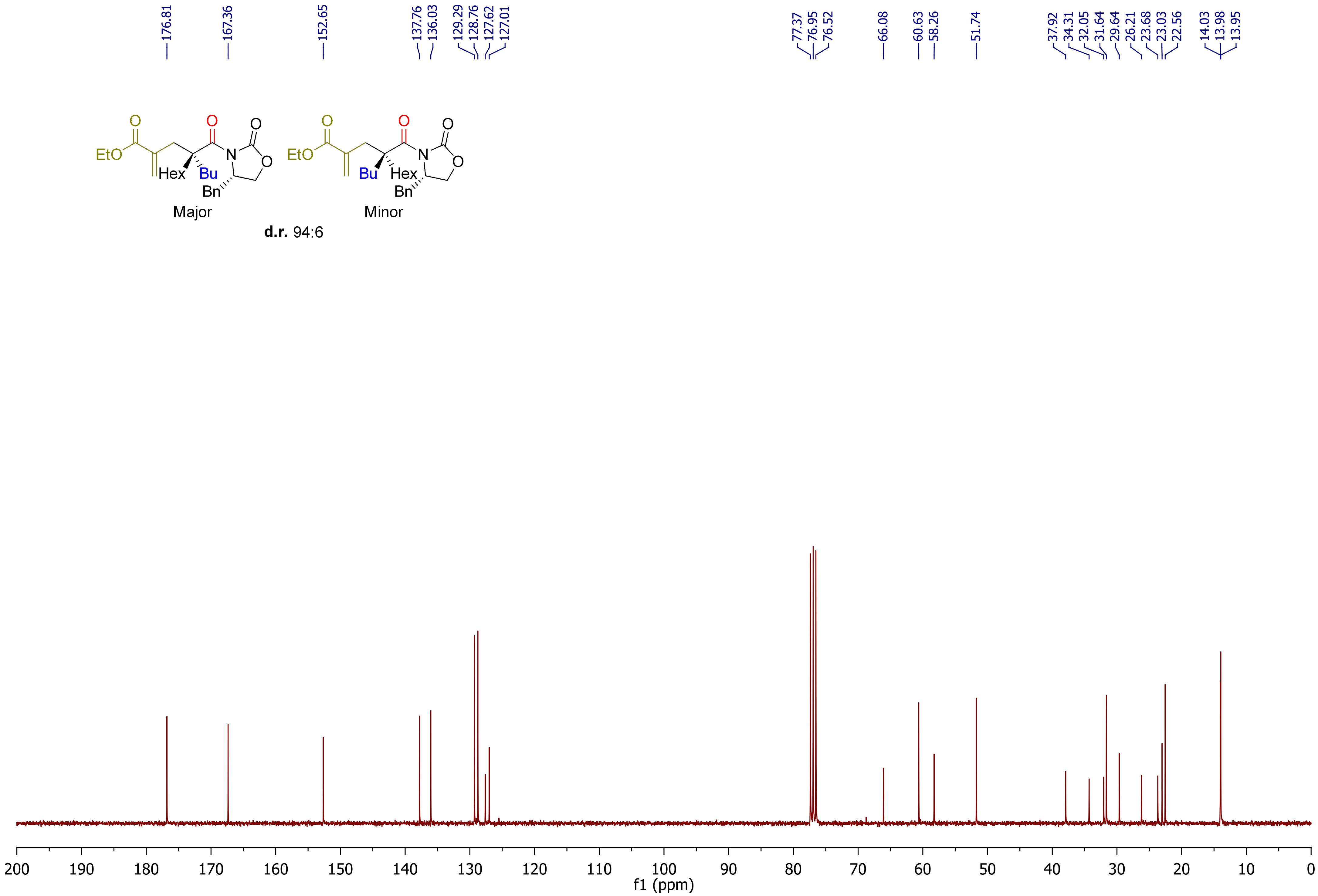
176.81
167.36
152.65
137.76
136.03
129.29
128.76
127.62
127.01
77.37
76.95
76.52
66.08
60.63
58.26
51.74
37.92
34.31
32.05
31.64
29.64
26.21
23.68
23.03
22.56
14.03
13.98
13.95
EtO
Hex
Bu
Bn
N
O
Major
Minor
d.r. 94:6
200 190 180 170 160 150 140 130 120 110 100 90 80 70 60 50 40 30 20 10 0
f1 (ppm)

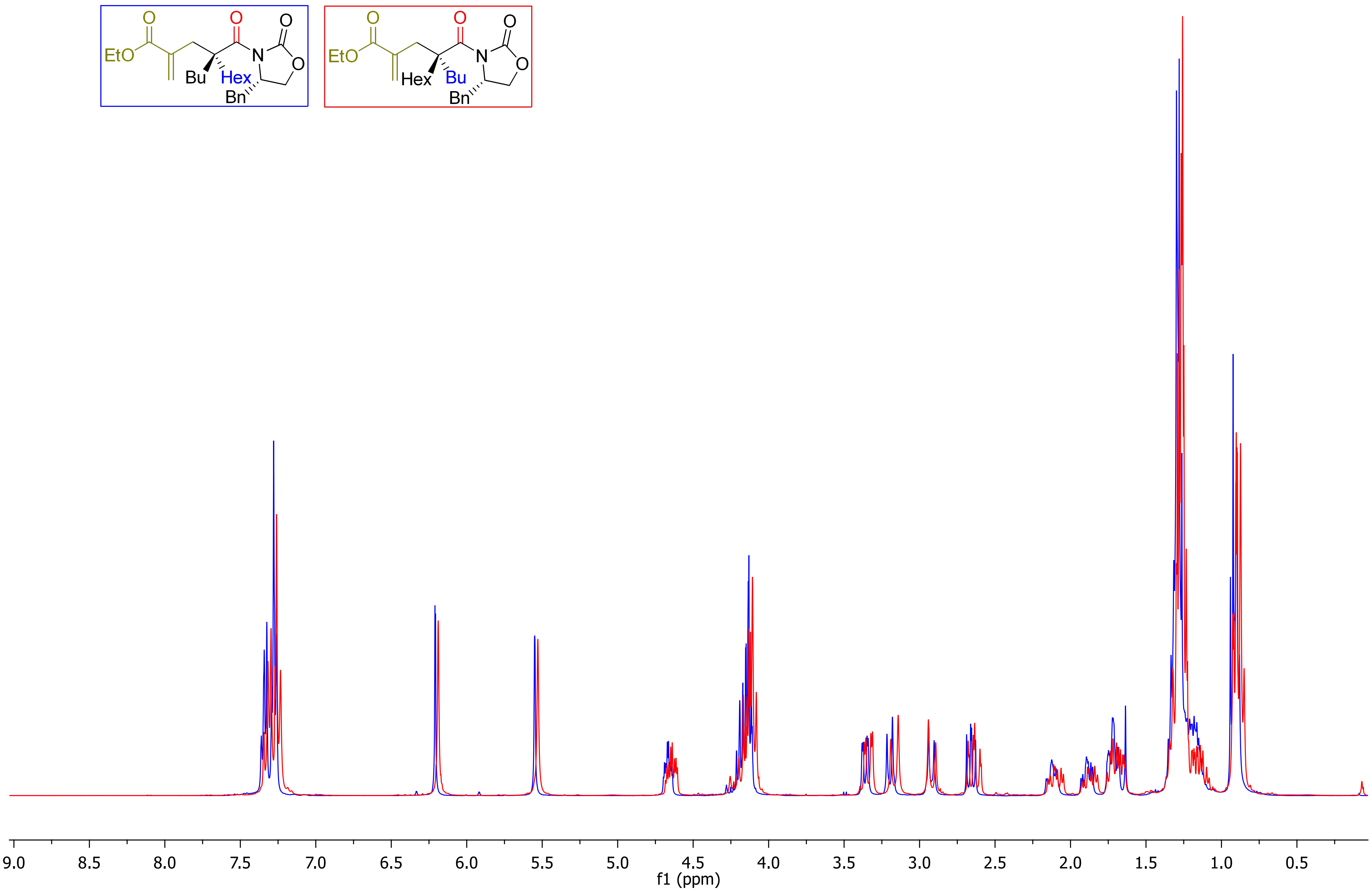

EtO
Bu
Hex
Bn
N
O
EtO
Hex
Bu
Bn
N
O
9.0
8.5
8.0
7.5
7.0
6.5
6.0
5.5
5.0
4.5
4.0
3.5
3.0
2.5
2.0
1.5
1.0
0.5
f1 (ppm)

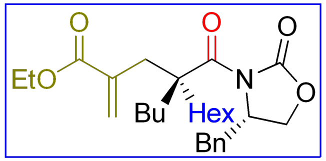
O
O
O
EtO
N
O
Bu
Hex
Bn

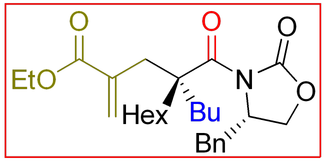
O
O
O
EtO
N
O
Hex
Bu
Bn

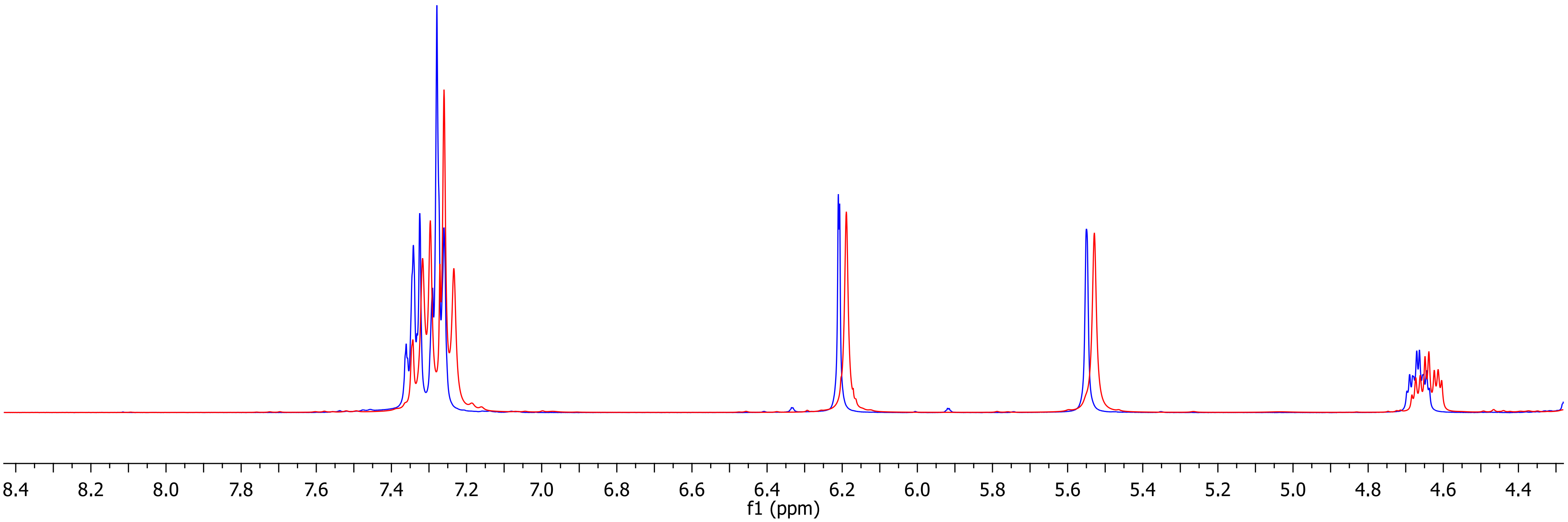
8.4
8.2
8.0
7.8
7.6
7.4
7.2
7.0
6.8
6.6
6.4
6.2
6.0
5.8
5.6
5.4
5.2
5.0
4.8
4.6
4.4
f1 (ppm)

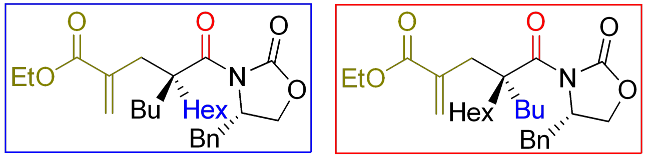
EtO
O
O
N
O
O
Bu
Hex
Bn
EtO
O
O
N
O
O
Hex
Bu
Bn

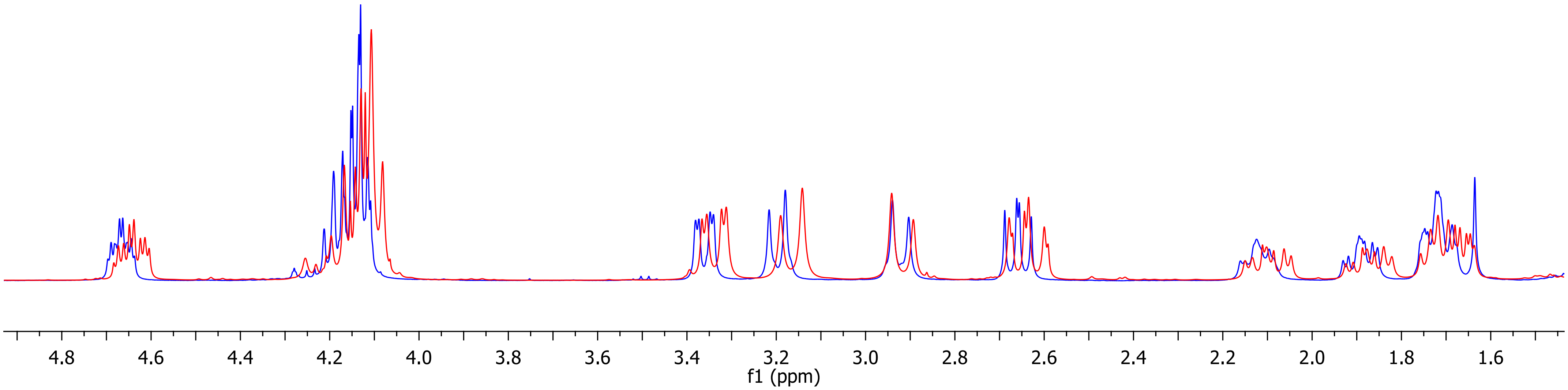
4.8
4.6
4.4
4.2
4.0
3.8
3.6
3.4
3.2
3.0
2.8
2.6
2.4
2.2
2.0
1.8
1.6
f1 (ppm)

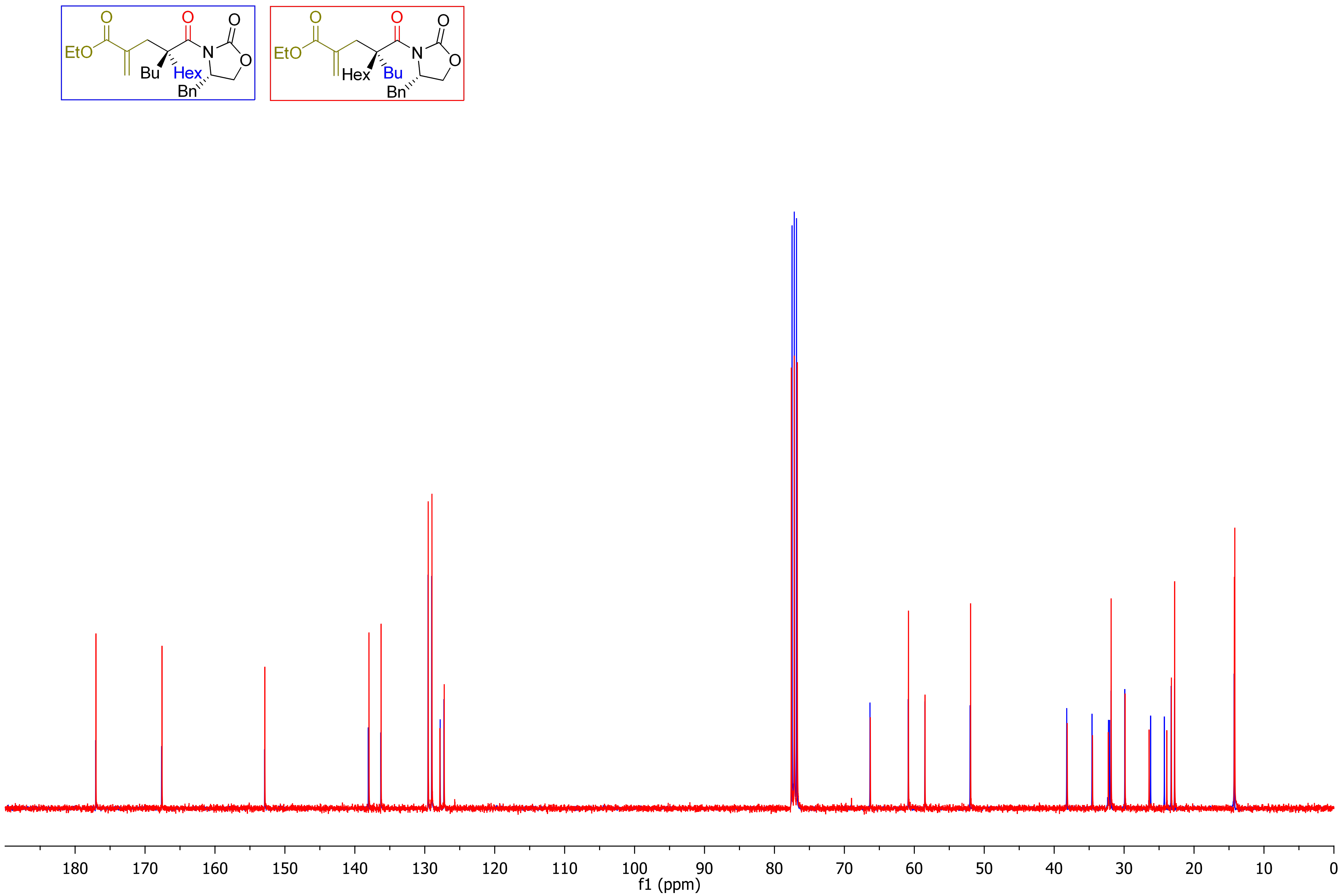

EtO
Bu
Hex
Bn
N
O
180
170
160
150
140
130
120
110
100
90
80
70
60
50
40
30
20
10
0
f1 (ppm)

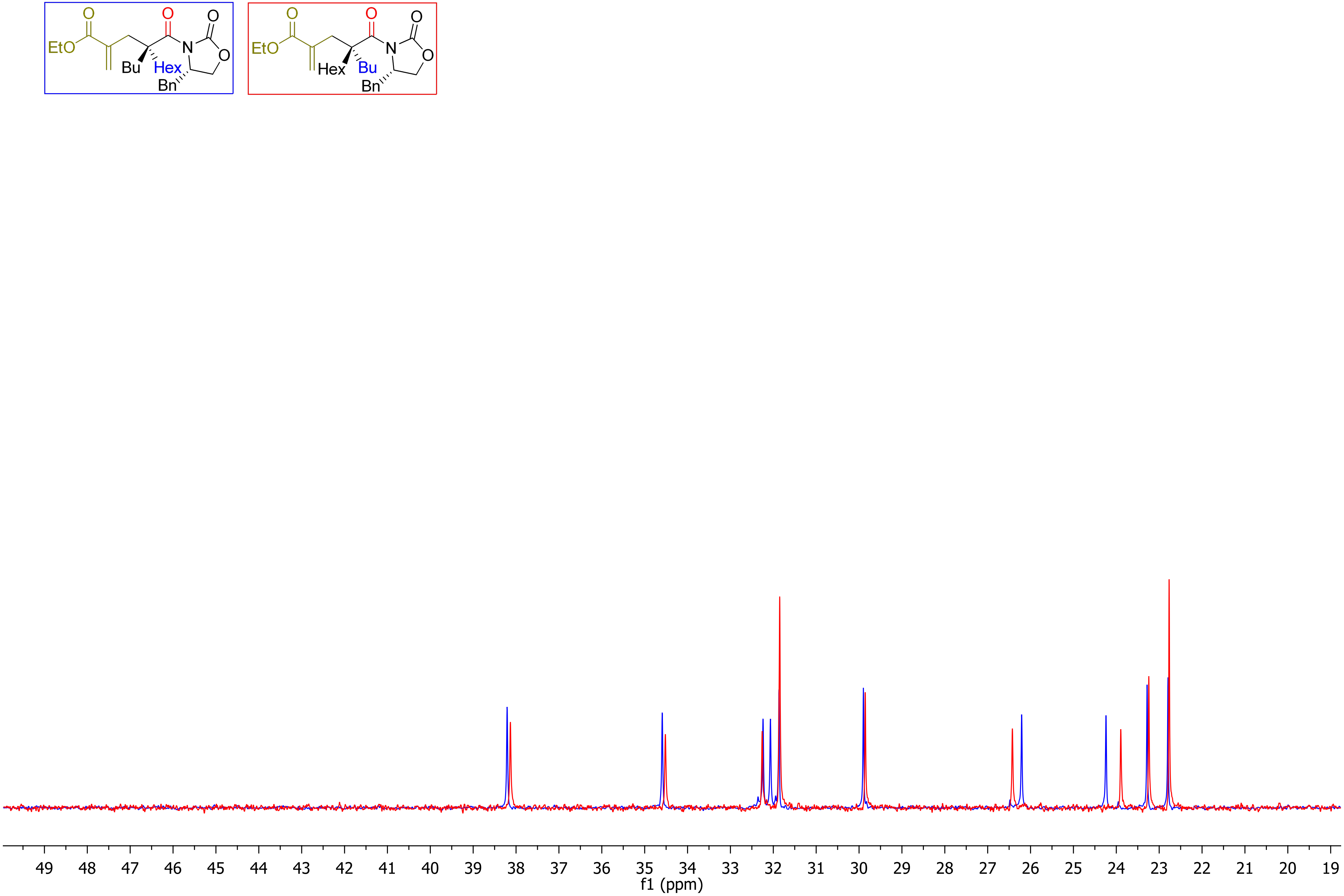
EtO
O
N
Bu
Hex
Bn
EtO
O
N
Hex
Bu
Bn
49 48 47 46 45 44 43 42 41 40 39 38 37 36 35 34 33 32 31 30 29 28 27 26 25 24 23 22 21 20 19
f1 (ppm)

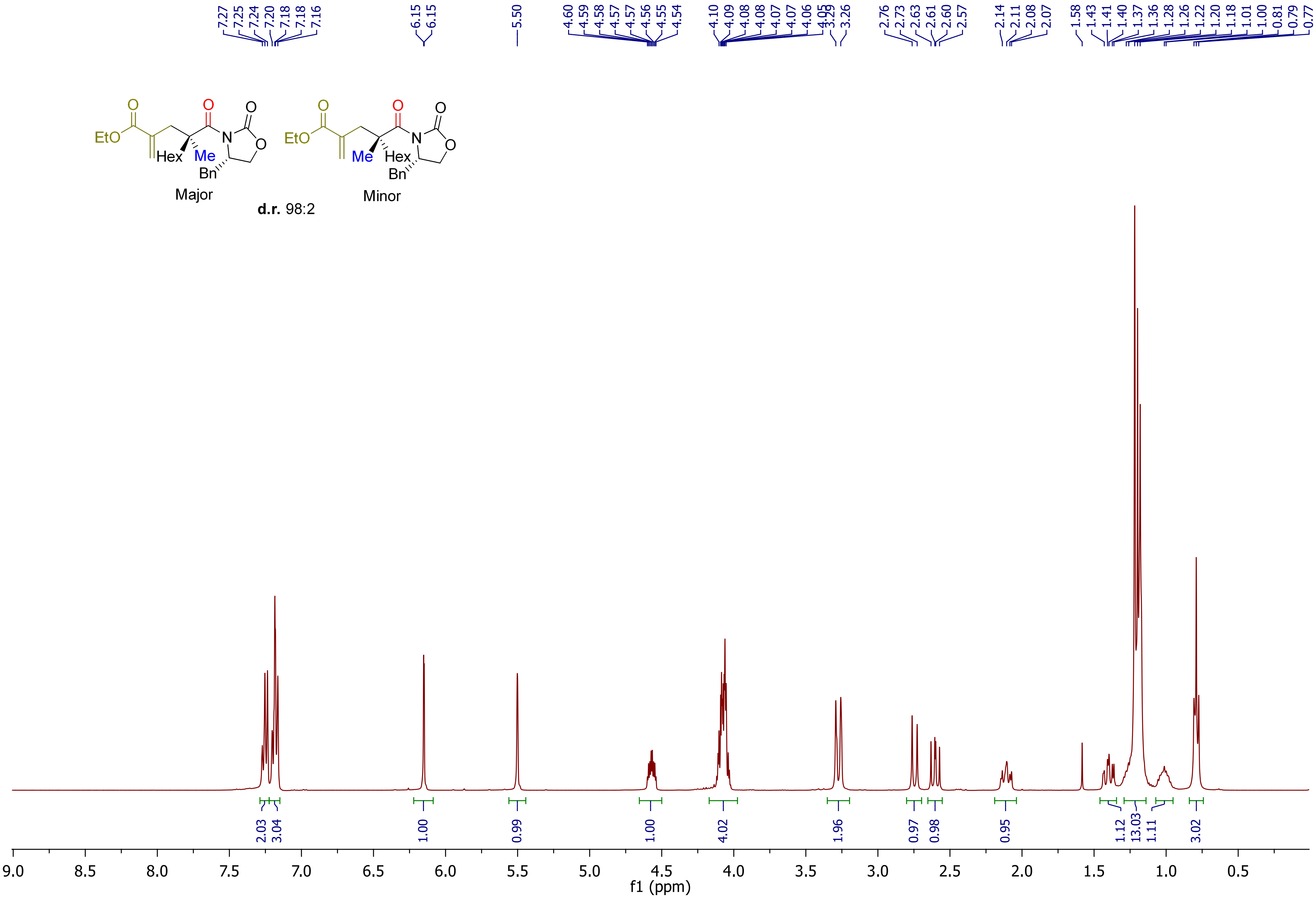

7.27 7.25 7.24 7.20 7.18 7.18 7.16
6.15 6.15
5.50
4.60 4.59 4.58 4.57 4.57 4.56 4.55 4.54
4.10 4.09 4.08 4.08 4.07 4.07 4.06 4.05 3.29 3.26
2.76 2.73 2.63 2.61 2.60 2.57
2.14 2.11 2.08 2.07
1.58 1.43 1.41 1.40 1.37 1.36 1.28 1.26 1.22 1.20 1.18 1.01 1.00 0.81 0.79 0.77
EtO
O
O
O
N
Hex
Me
Bn
Major
Minor
d.r. 98:2
2.03 3.04 1.00 0.99 1.00 4.02 1.96 0.97 0.98 0.95 1.12 13.03 1.11 3.02
9.0 8.5 8.0 7.5 7.0 6.5 6.0 5.5 5.0 4.5 4.0 3.5 3.0 2.5 2.0 1.5 1.0 0.5
f1 (ppm)

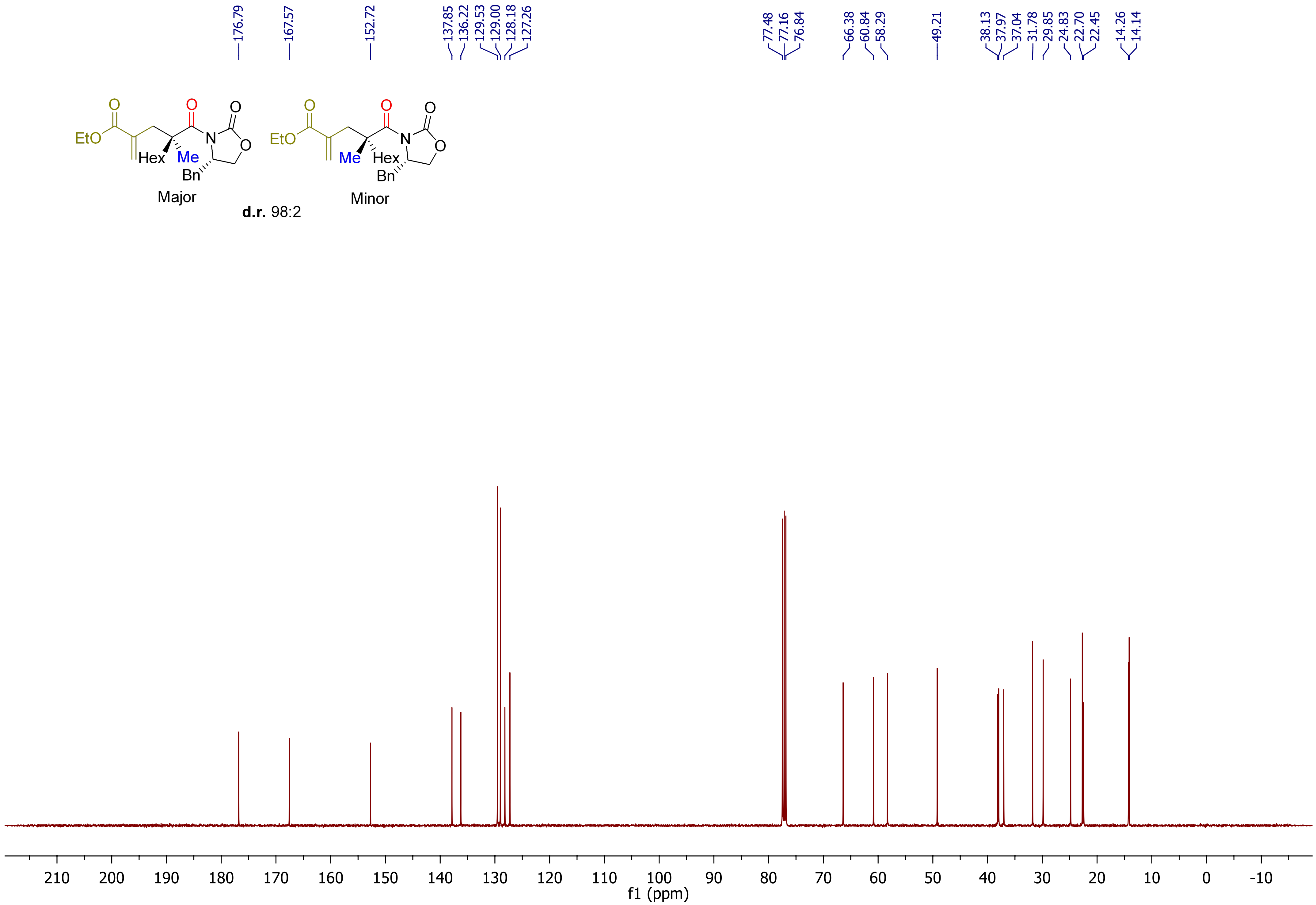

176.79
167.57
152.72
137.85
136.22
129.53
129.00
128.18
127.26
77.48
77.16
76.84
66.38
60.84
58.29
49.21
38.13
37.97
37.04
31.78
29.85
24.83
22.70
22.45
14.26
14.14
EtO
Hex
Me
Bn
Major
d.r. 98:2
Minor
f1 (ppm)

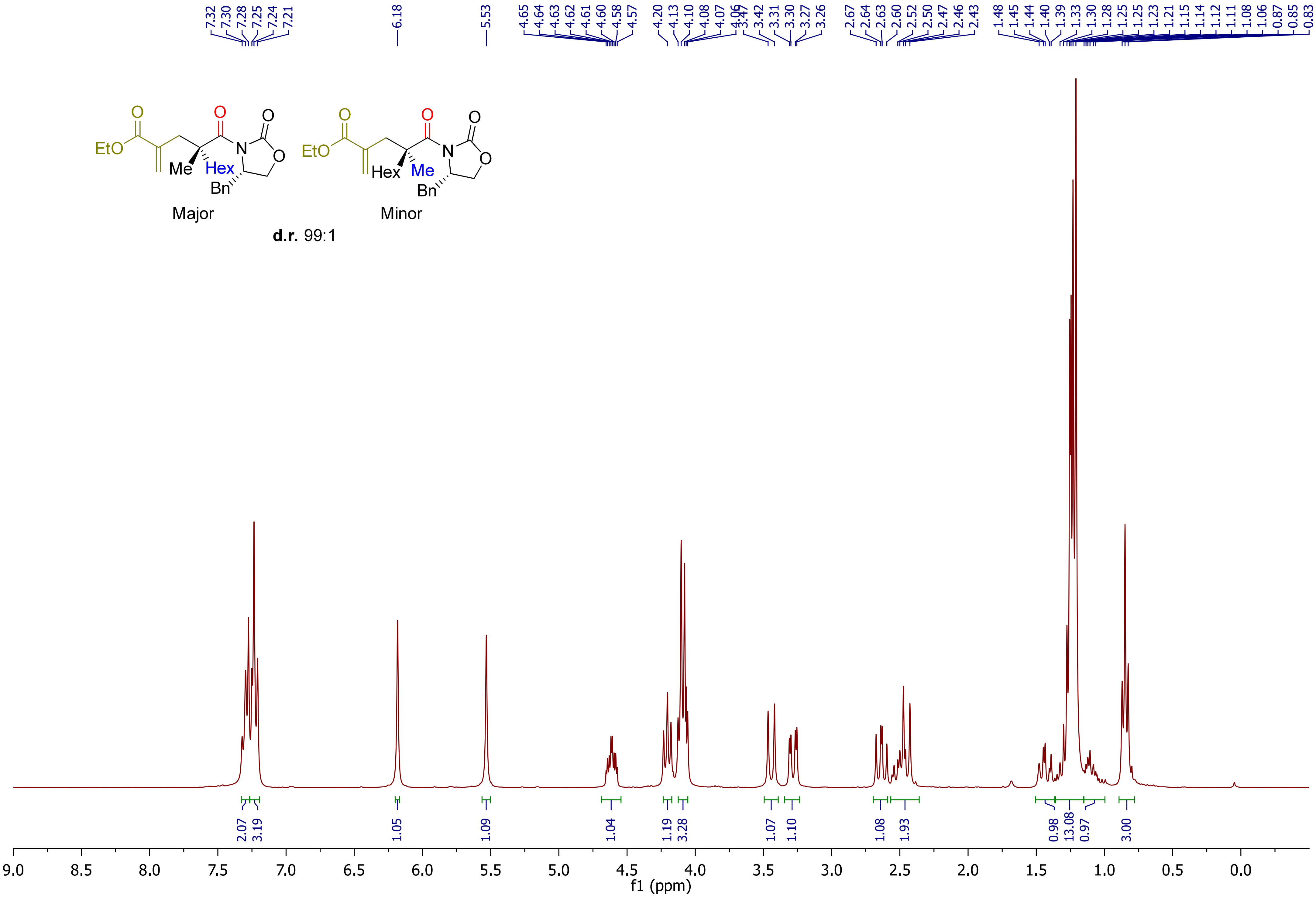
7.32 7.30 7.28 7.25 7.24 7.21
6.18
5.53
4.65 4.64 4.63 4.62 4.61 4.60 4.58 4.57
4.20 4.13 4.10 4.08 4.07 4.06 3.47 3.42 3.31 3.30 3.27 3.26
2.67 2.64 2.63 2.60 2.52 2.50 2.47 2.46 2.43
1.48 1.45 1.44 1.40 1.39 1.33 1.30 1.28 1.25 1.25 1.23 1.21 1.15 1.14 1.12 1.11 1.08 1.06 0.87 0.85 0.83
EtO
Me
Hex
Bn
Major
Minor
d.r. 99:1
2.07 3.19 1.05 1.09 1.04 1.19 3.28 1.07 1.10 1.08 1.93 0.98 13.08 0.97 3.00
9.0 8.5 8.0 7.5 7.0 6.5 6.0 5.5 5.0 4.5 4.0 3.5 3.0 2.5 2.0 1.5 1.0 0.5 0.0
f1 (ppm)

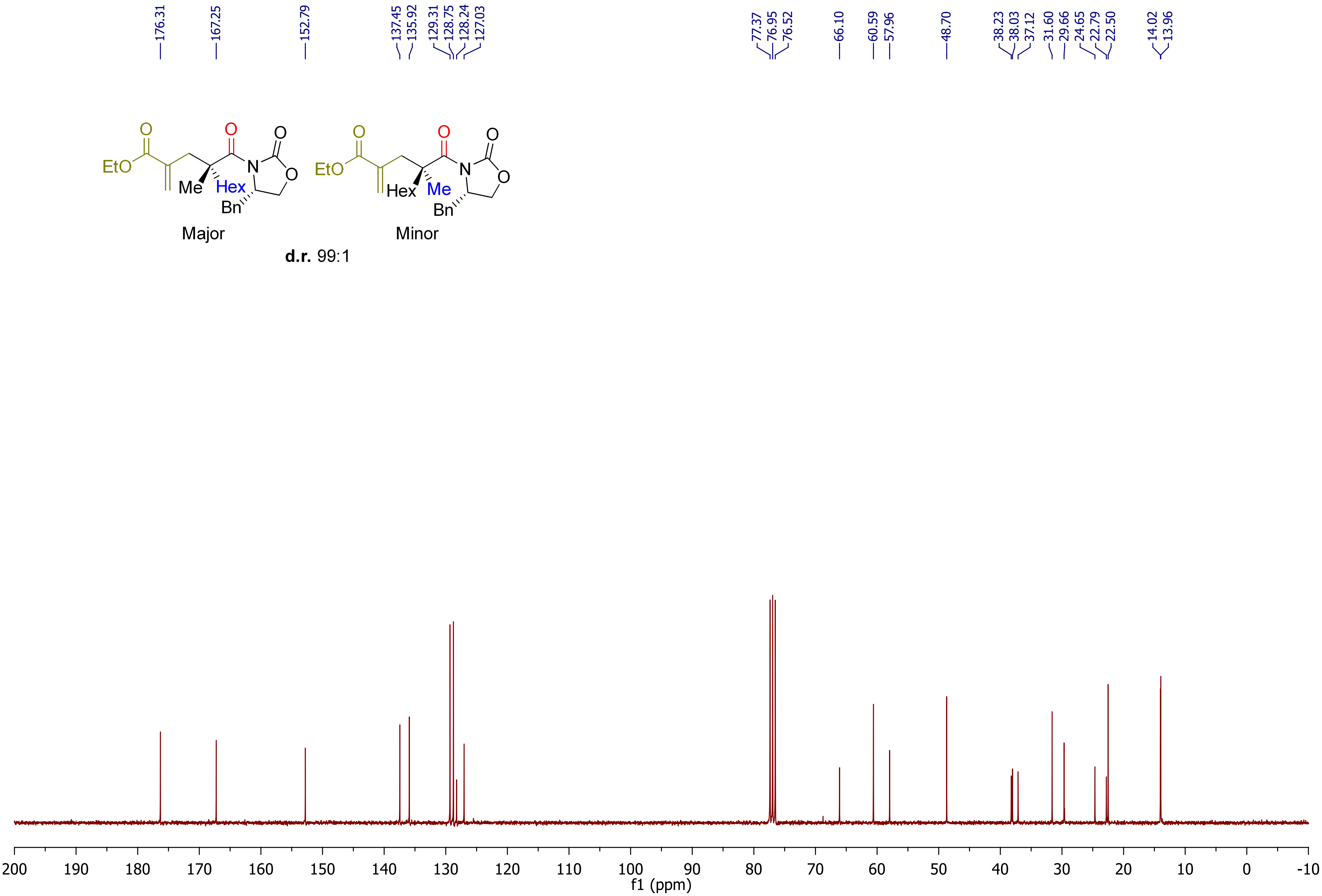

176.31
167.25
152.79
137.45
135.92
129.31
128.75
128.24
127.03
77.37
76.95
76.52
66.10
60.59
57.96
48.70
38.23
38.03
37.12
31.60
29.66
24.65
22.79
22.50
14.02
13.96
EtO
O
O
N
O
O
Me
Hex
Bn
Major
Hex
Me
Minor
d.r. 99:1
200
190
180
170
160
150
140
130
120
110
100
90
80
70
60
50
40
30
20
10
0
-10
f1 (ppm)

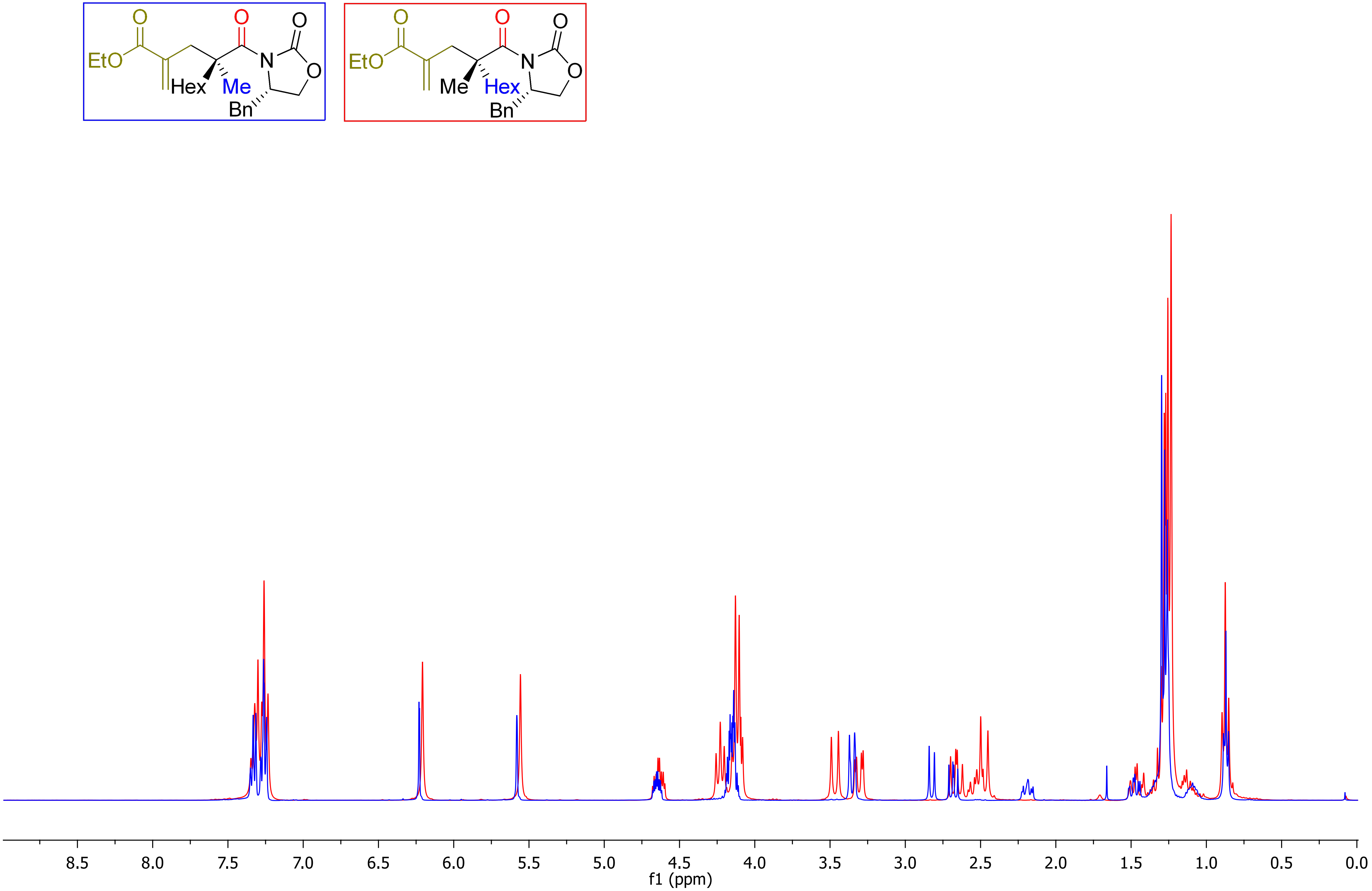

EtO
Hex
Me
Bn
8.5 8.0 7.5 7.0 6.5 6.0 5.5 5.0 4.5 4.0 3.5 3.0 2.5 2.0 1.5 1.0 0.5 0.0
f1 (ppm)

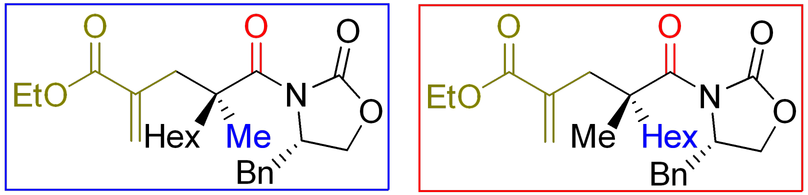
EtO
O
O
O
N
O
Hex
Me
Bn
EtO
O
O
O
N
O
Me
Hex
Bn

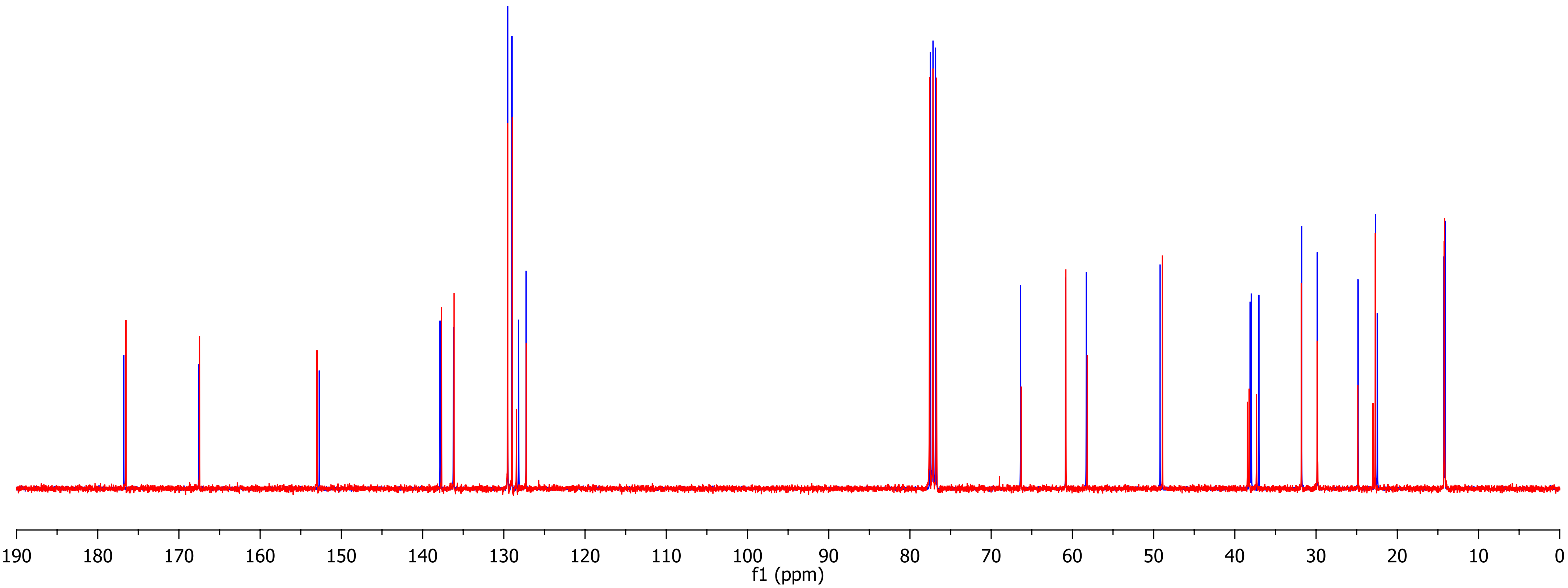
190
180
170
160
150
140
130
120
110
100
90
80
70
60
50
40
30
20
10
0
f1 (ppm)

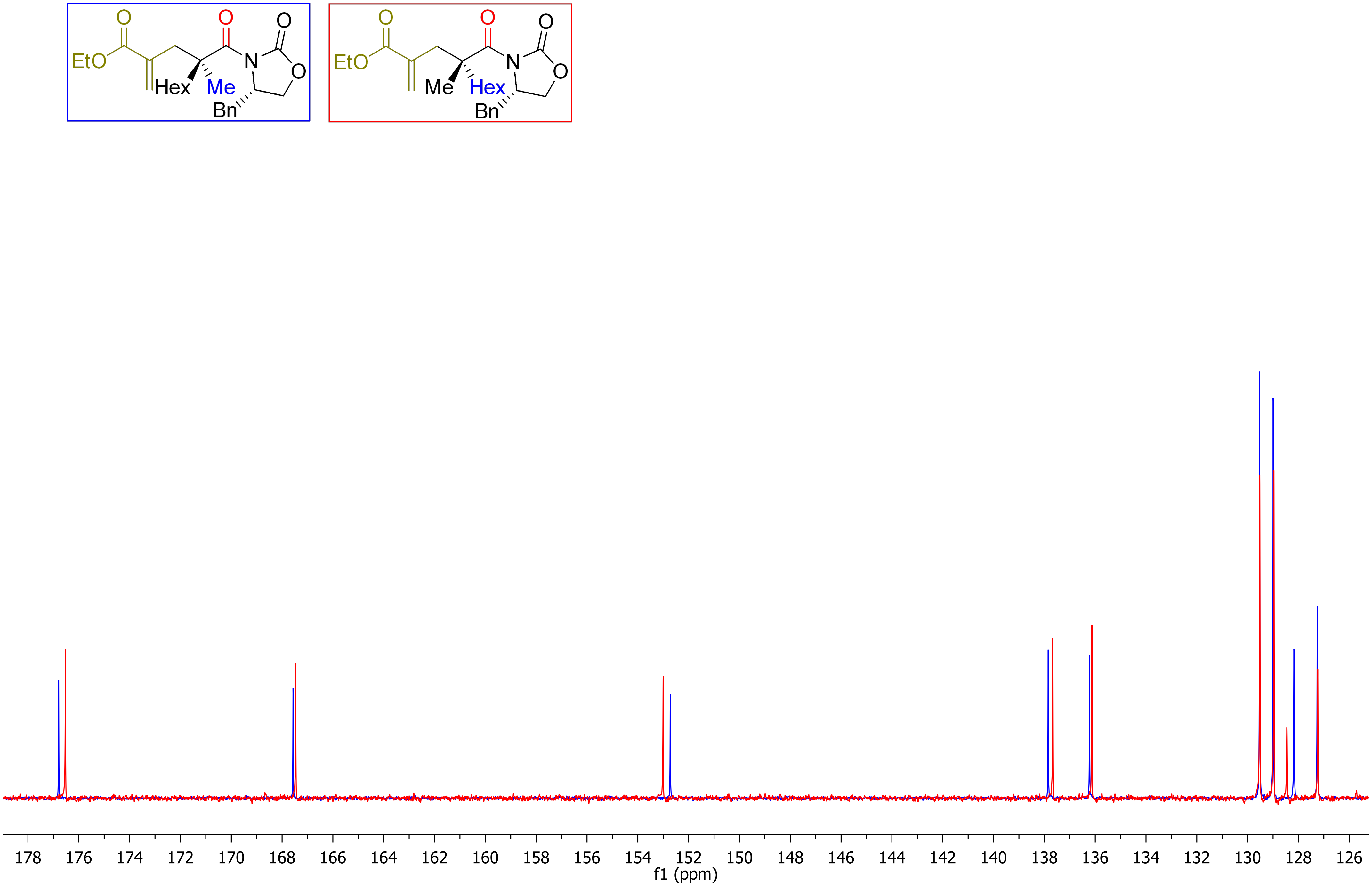
EtO
Hex
Me
Bn
N
O
Me
Hex
178
176
174
172
170
168
166
164
162
160
158
156
154
152
150
148
146
144
142
140
138
136
134
132
130
128
126
f1 (ppm)

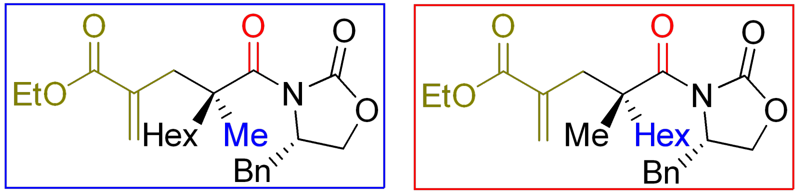
O
O
O
EtO
N
O
Hex
Me
Bn
O
O
O
EtO
N
O
Me
Hex
Bn

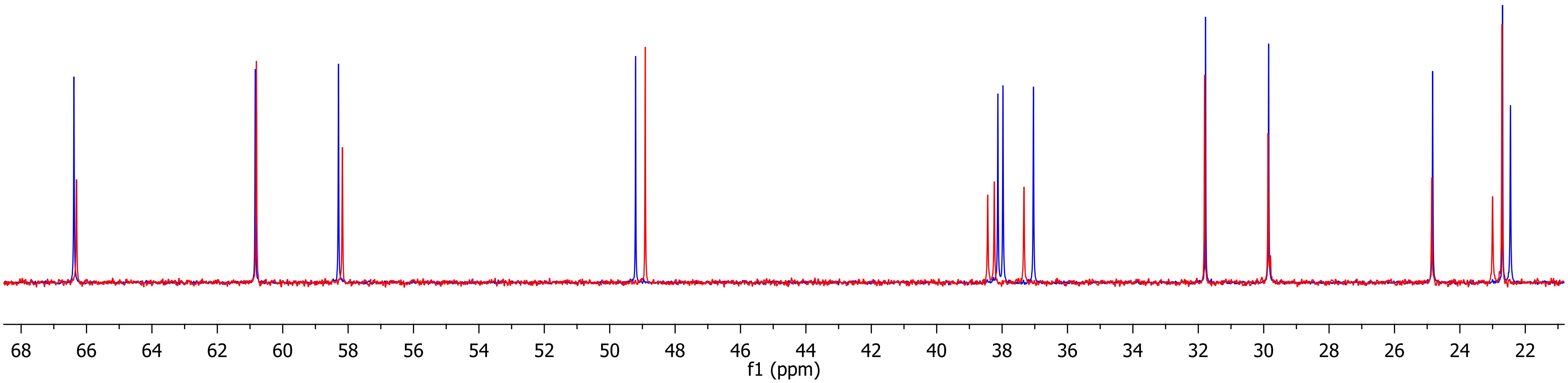
68
66
64
62
60
58
56
54
52
50
48
46
44
42
40
38
36
34
32
30
28
26
24
22
f1 (ppm)

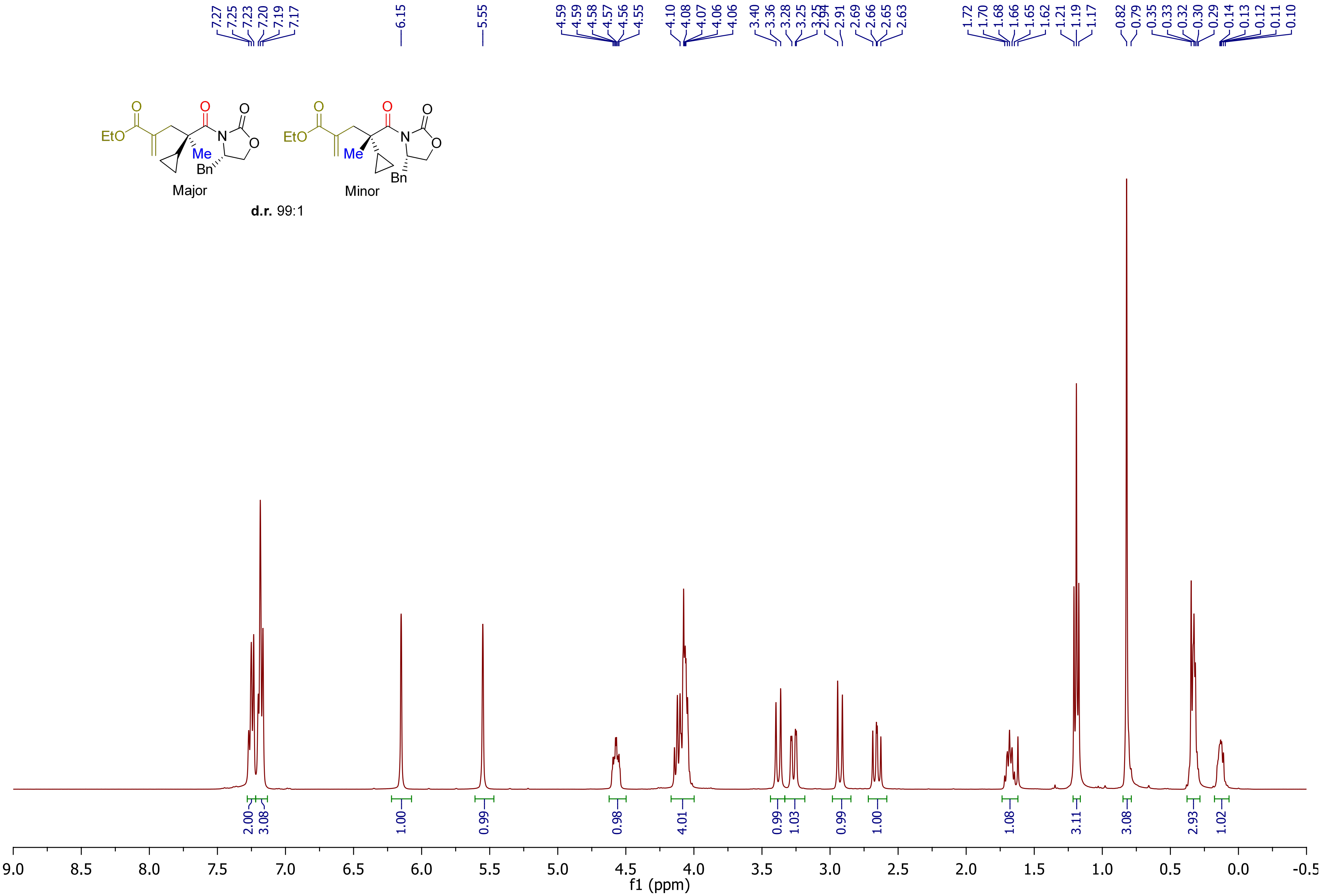

EtO
Me
Bn
N
O
Major
Minor
d.r. 99:1
7.27 7.25 7.23 7.20 7.19 7.17
6.15
5.55
4.59 4.59 4.58 4.57 4.56 4.55
4.10 4.08 4.07 4.06 4.06
3.40 3.36 3.28 3.25 3.25 2.94 2.91 2.69 2.66 2.65 2.63
1.72 1.70 1.68 1.66 1.65 1.62 1.21 1.19 1.17
0.82 0.79 0.35 0.33 0.32 0.30 0.29 0.14 0.13 0.12 0.11 0.10
2.00 3.08 1.00 0.99 0.98 4.01 0.99 1.03 0.99 1.00 1.08 3.11 3.08 2.93 1.02
9.0 8.5 8.0 7.5 7.0 6.5 6.0 5.5 5.0 4.5 4.0 3.5 3.0 2.5 2.0 1.5 1.0 0.5 0.0 -0.5
f1 (ppm)

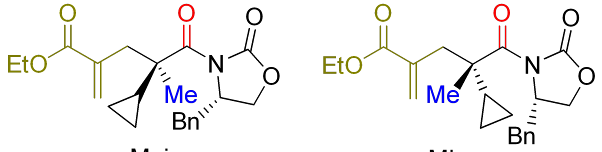

Major Minor

d.r. 99:1

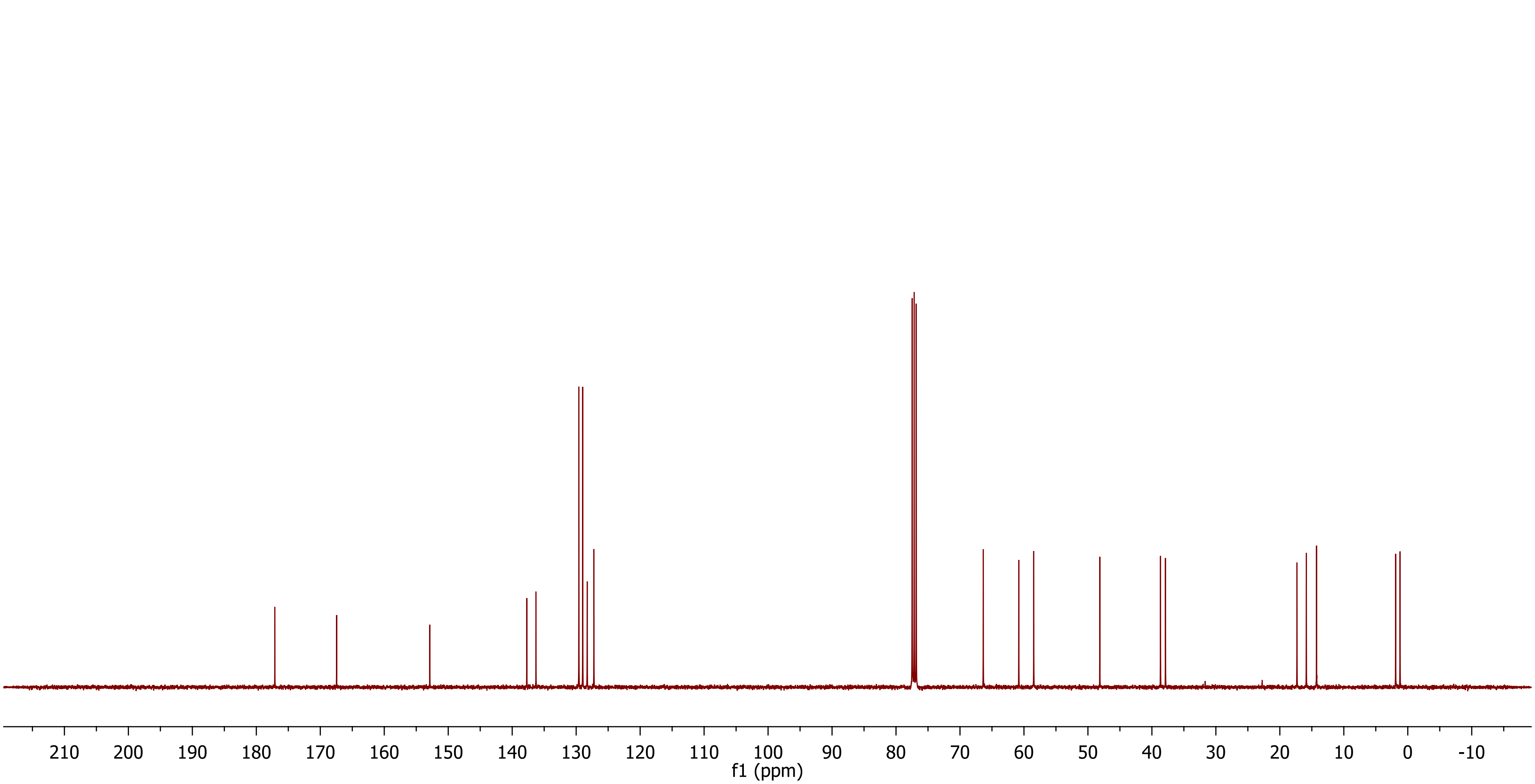

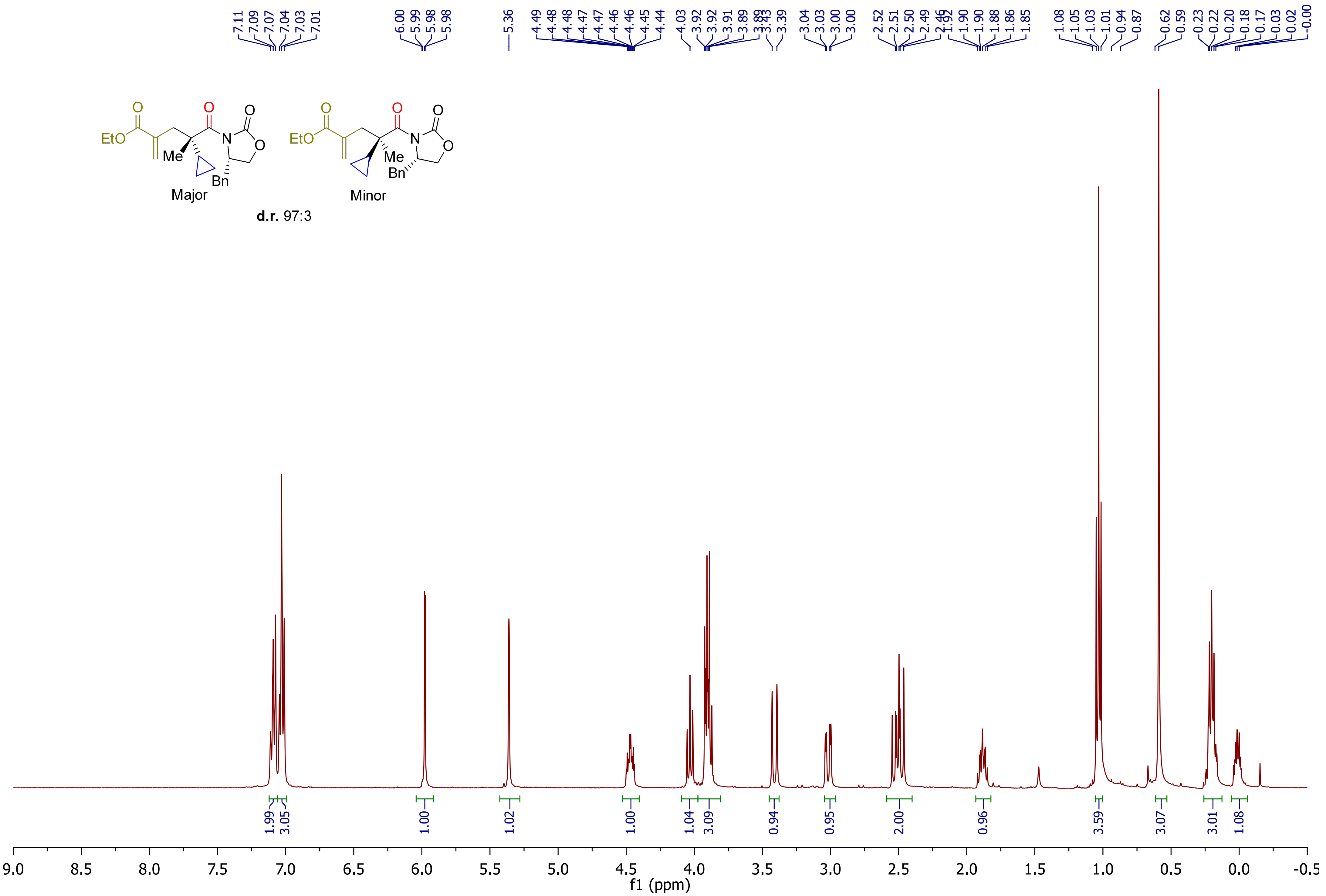

EtO
Me
Bn
Major
Minor
d.r. 97:3
f1 (ppm)

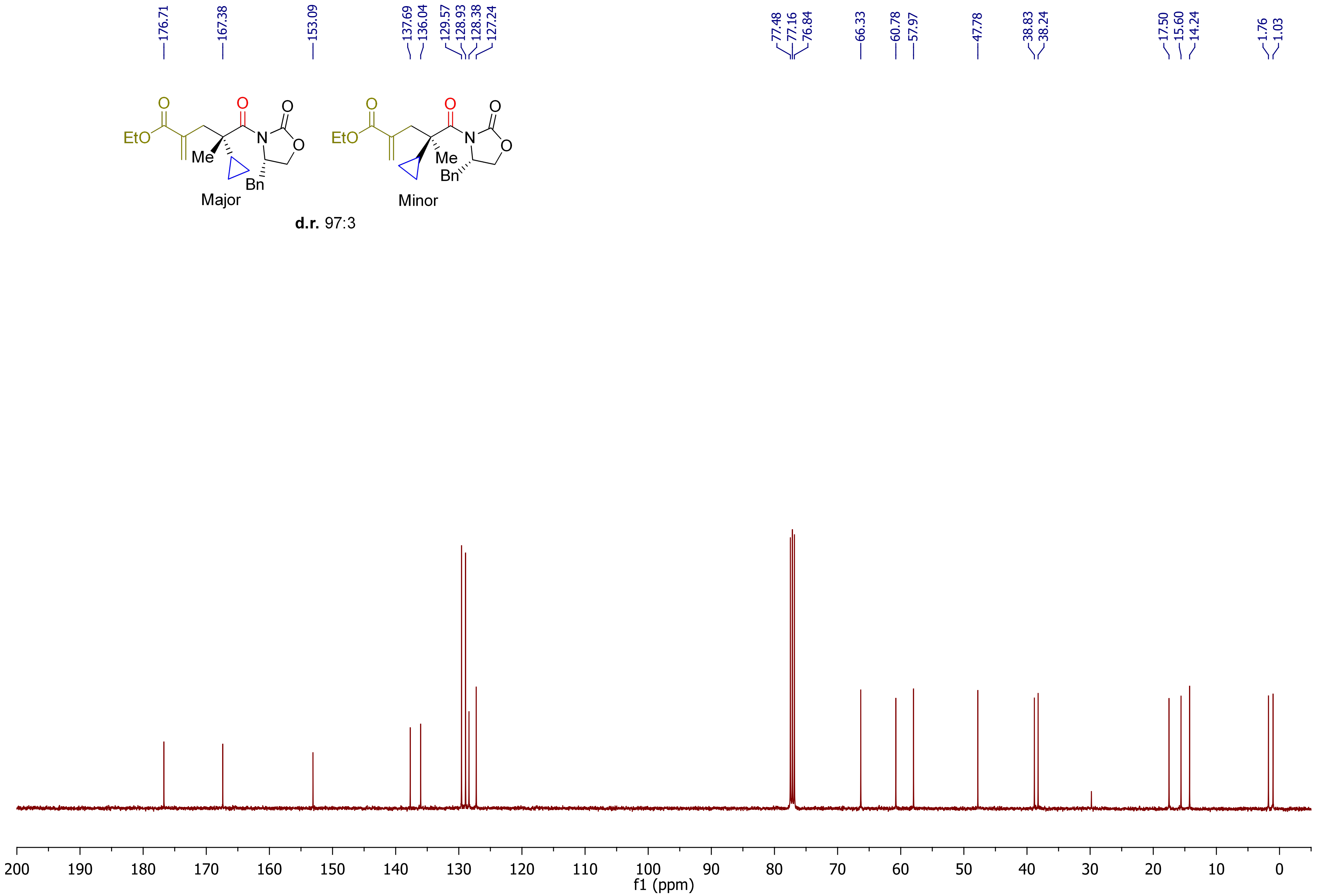

176.71
167.38
153.09
137.69
136.04
129.57
128.93
128.38
127.24
77.48
77.16
76.84
66.33
60.78
57.97
47.78
38.83
38.24
17.50
15.60
14.24
1.76
1.03
EtO
O
O
O
N
O
Me
Bn
Major
Minor
d.r. 97:3
200 190 180 170 160 150 140 130 120 110 100 90 80 70 60 50 40 30 20 10 0
f1 (ppm)

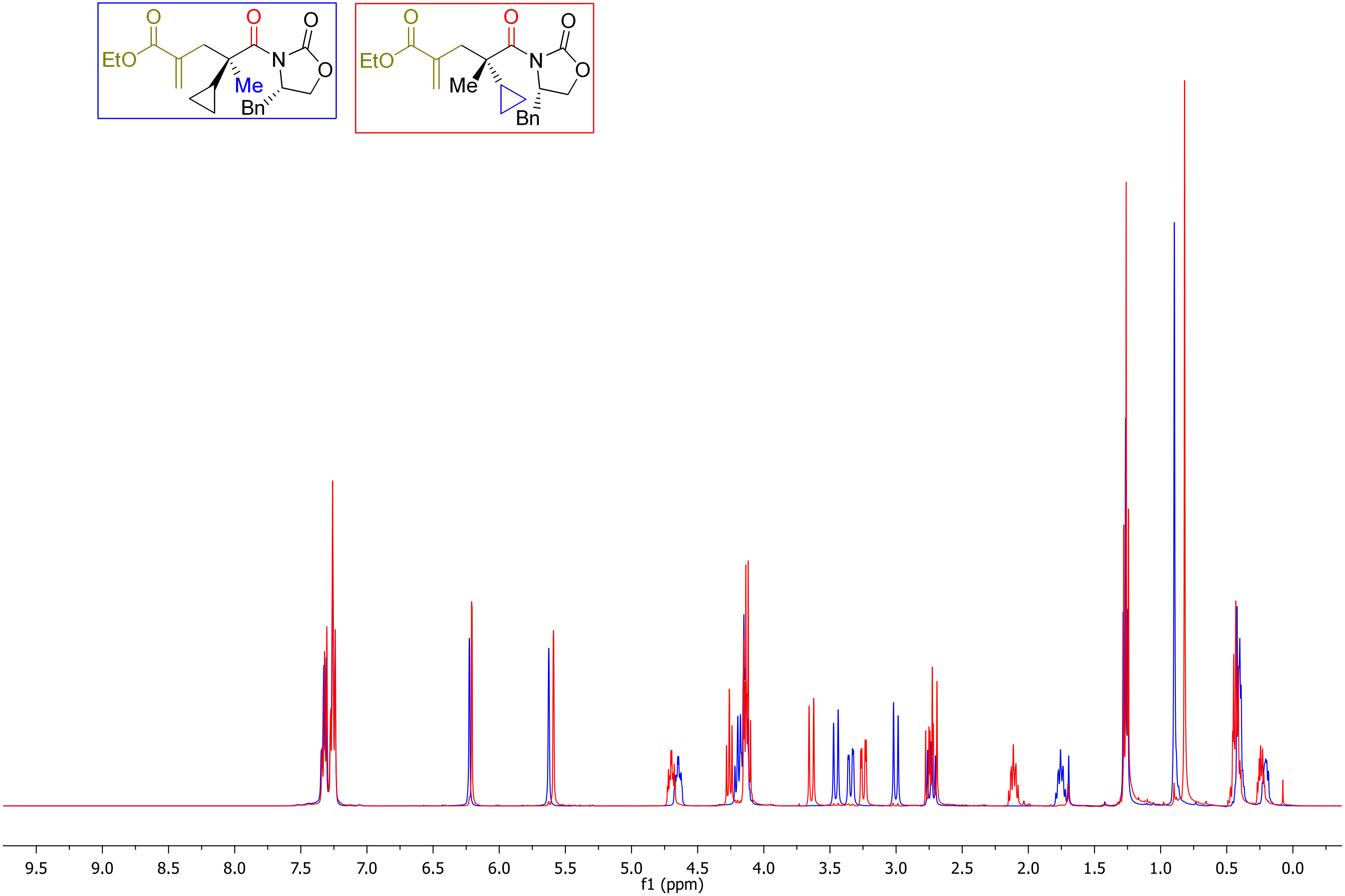
EtO
O
O
N
O
O
Me
Bn
EtO
O
O
N
O
O
Me
Bn
9.5
9.0
8.5
8.0
7.5
7.0
6.5
6.0
5.5
5.0
4.5
4.0
3.5
3.0
2.5
2.0
1.5
1.0
0.5
0.0
f1 (ppm)

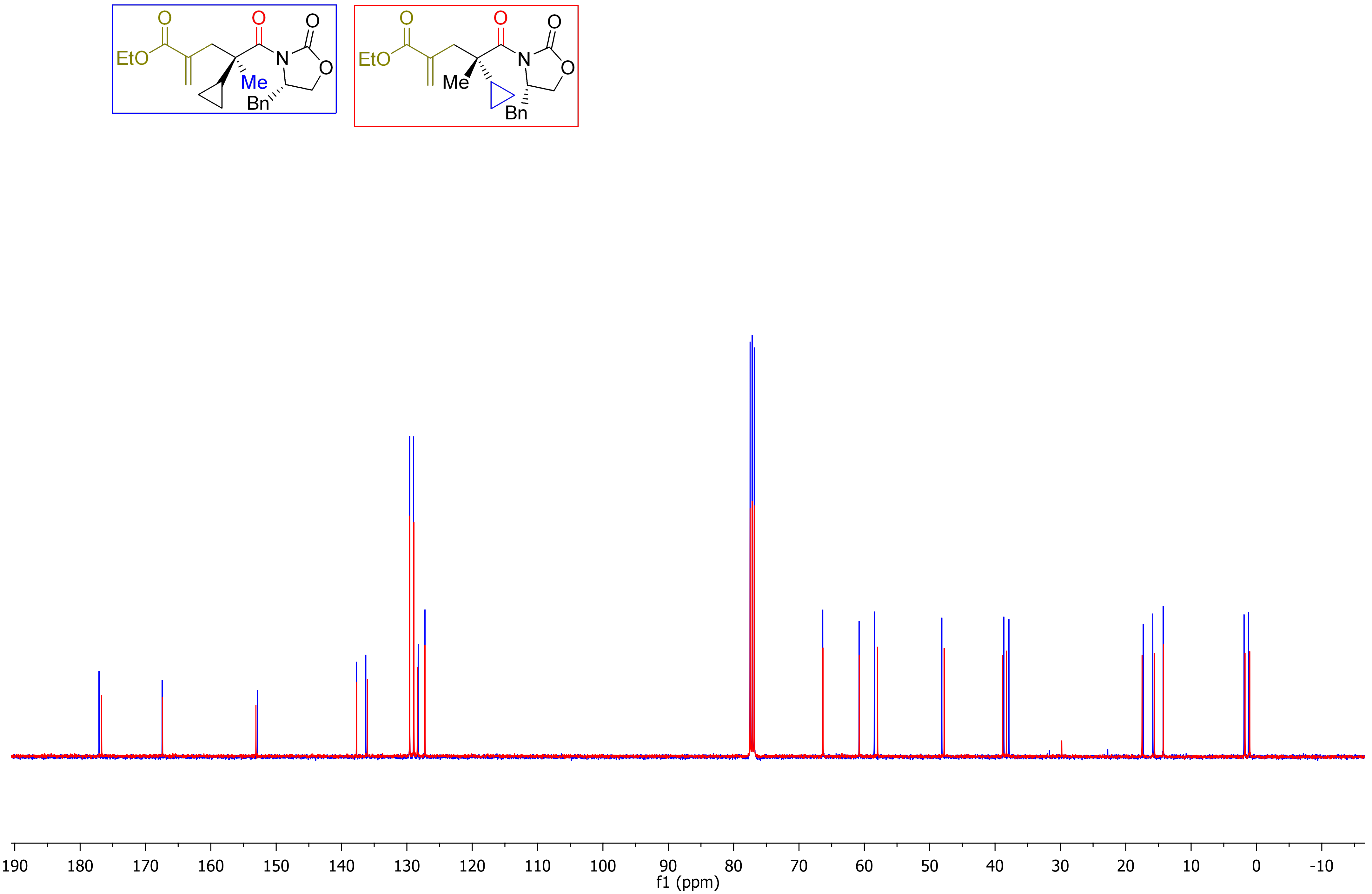
EtO
O
O
N
O
O
Me
Bn
EtO
O
O
N
O
O
Me
Bn
190
180
170
160
150
140
130
120
110
100
90
80
70
60
50
40
30
20
10
0
-10
f1 (ppm)

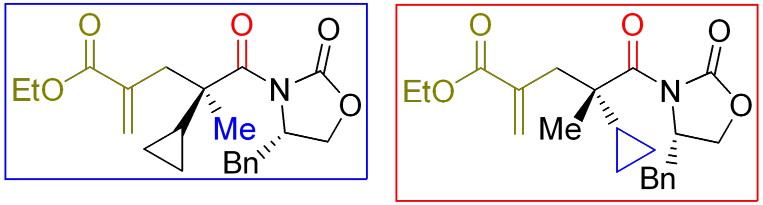
O
O
O
EtO
N
O
Me
Bn
O
O
O
EtO
N
O
Me
Bn

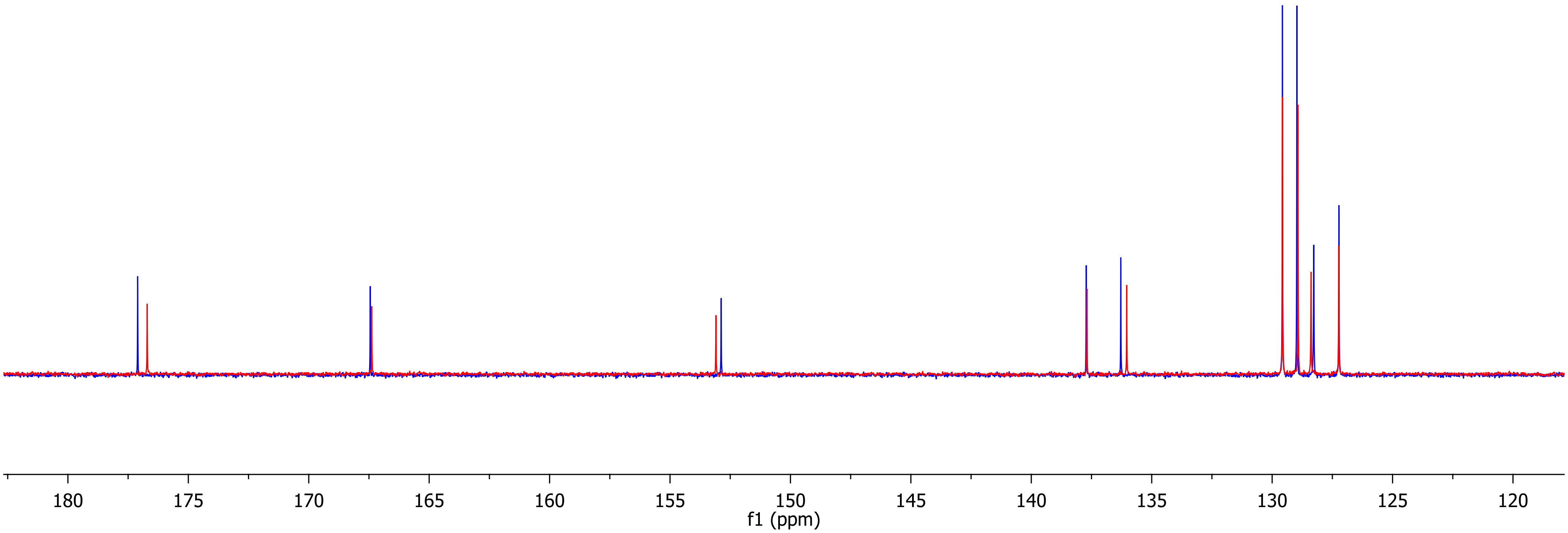
180
175
170
165
160
155
150
145
140
135
130
125
120
f1 (ppm)

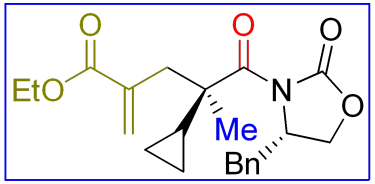
EtO
O
N
Me
Bn
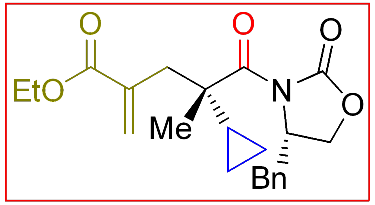
EtO
O
N
Me
Bn
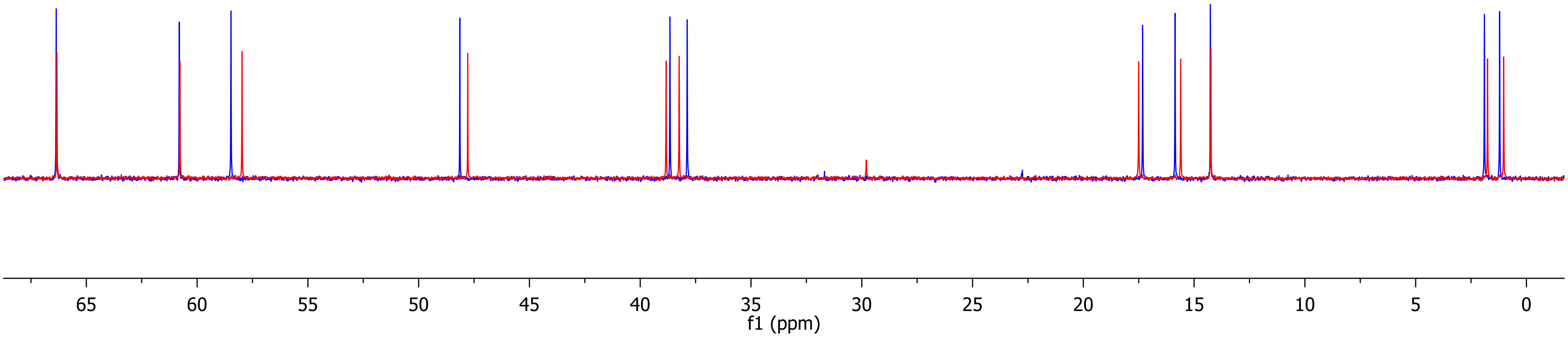
65
60
55
50
45
40
35
30
25
20
15
10
5
0
f1 (ppm)

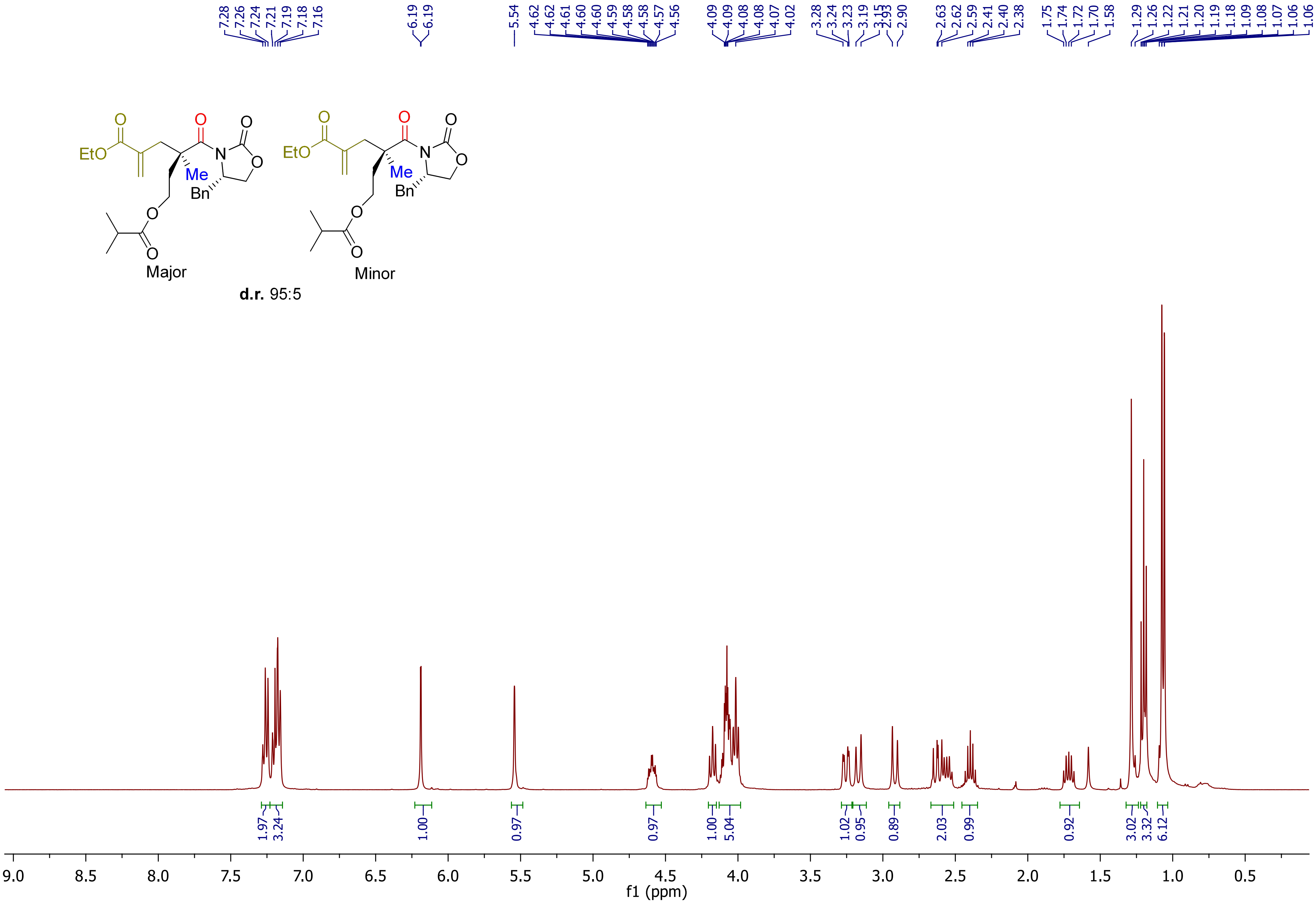

7.28
7.26
7.24
7.21
7.19
7.18
7.16
6.19
6.19
5.54
4.62
4.62
4.61
4.60
4.60
4.59
4.58
4.58
4.57
4.56
4.09
4.09
4.08
4.08
4.07
4.02
3.28
3.24
3.23
3.19
3.15
2.93
2.90
2.63
2.62
2.59
2.41
2.40
2.38
1.75
1.74
1.72
1.70
1.58
1.29
1.26
1.22
1.21
1.20
1.19
1.18
1.09
1.08
1.07
1.06
1.06
EtO
O
O
N
O
O
Me
Bn
O
O
Major
d.r. 95:5
EtO
O
O
N
O
O
Me
Bn
O
O
Minor
1.97
3.24
1.00
0.97
0.97
1.00
5.04
1.02
0.95
0.89
2.03
0.99
0.92
3.02
3.32
6.12
9.0
8.5
8.0
7.5
7.0
6.5
6.0
5.5
5.0
4.5
4.0
3.5
3.0
2.5
2.0
1.5
1.0
0.5
f1 (ppm)

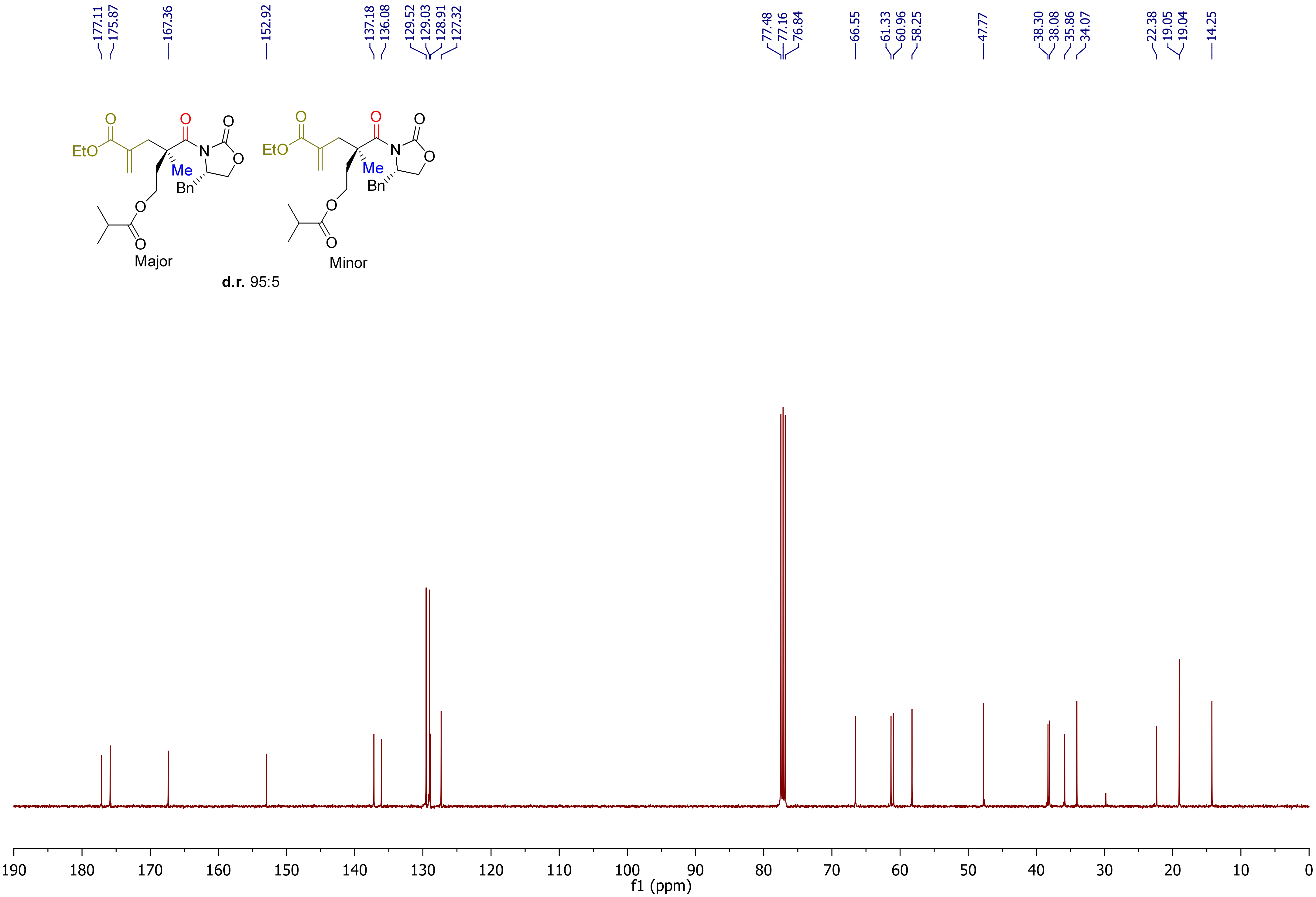
177.11
175.87
167.36
152.92
137.18
136.08
129.52
129.03
128.91
127.32
77.48
77.16
76.84
66.55
61.33
60.96
58.25
47.77
38.30
38.08
35.86
34.07
22.38
19.05
19.04
14.25
EtO
Me
Bn
Major
Minor
d.r. 95:5
f1 (ppm)

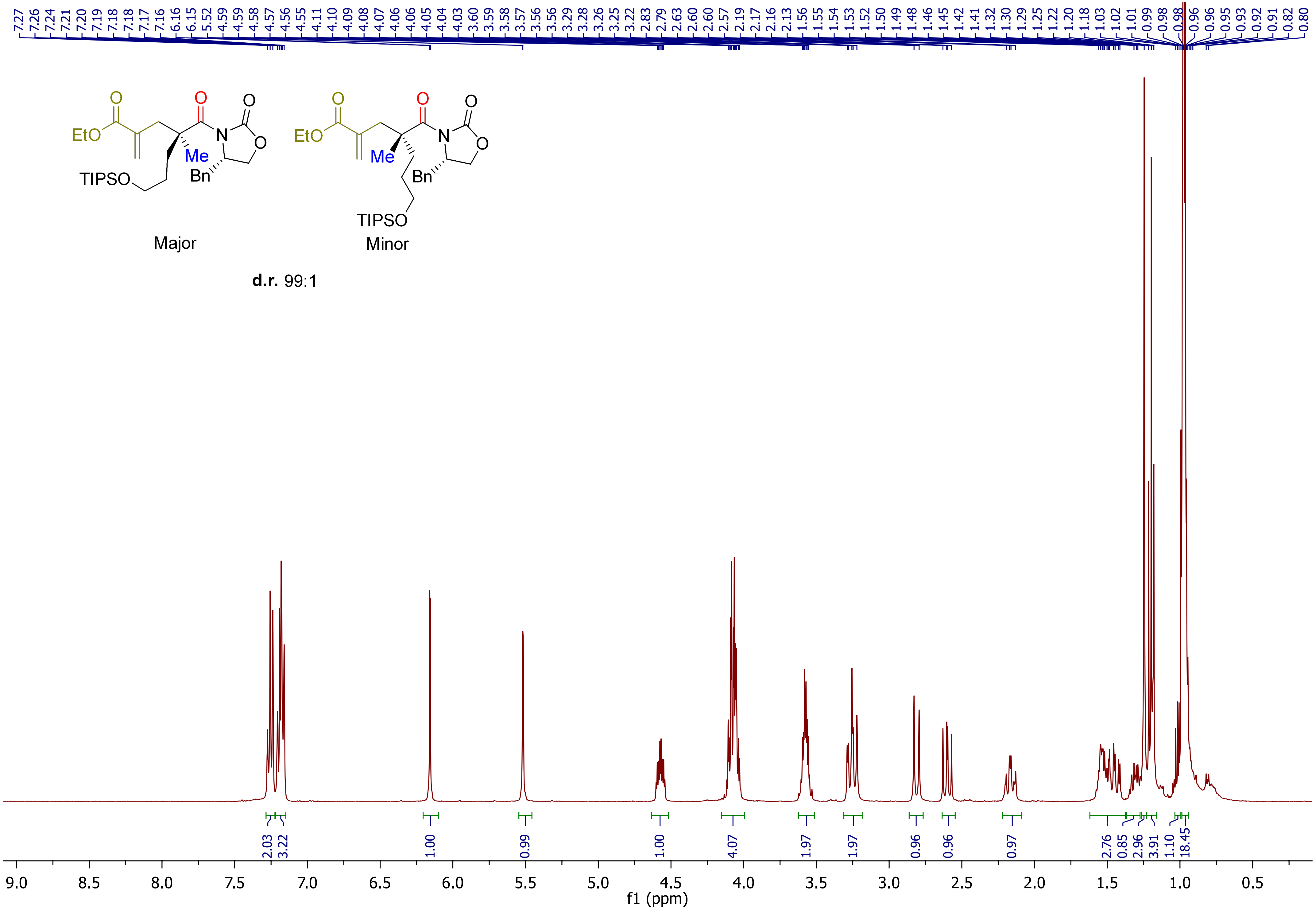

EtO
Me
Bn
TIPSO
Major
Minor
d.r. 99:1
f1 (ppm)

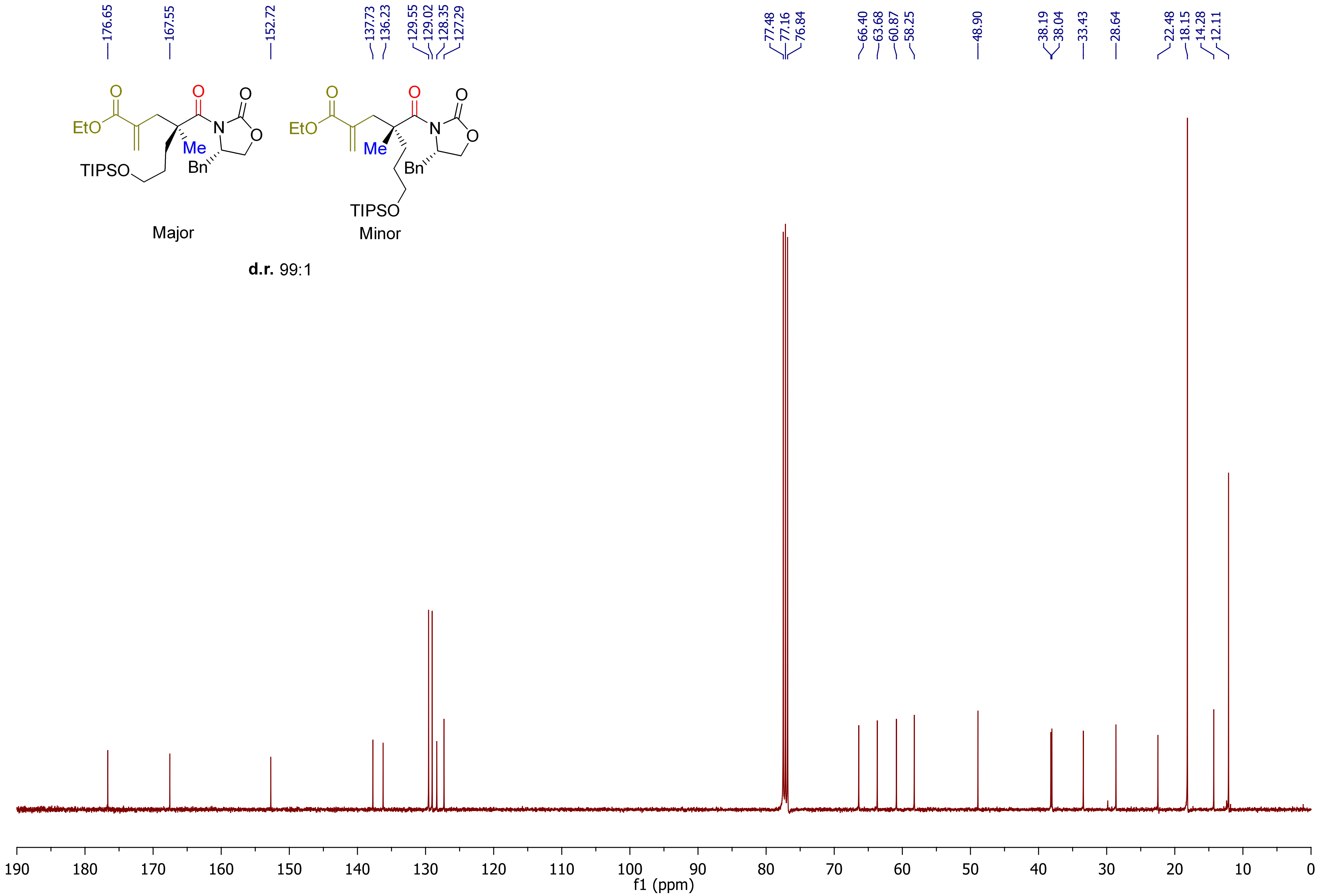
176.65
167.55
152.72
137.73
136.23
129.55
129.02
128.35
127.29
77.48
77.16
76.84
66.40
63.68
60.87
58.25
48.90
38.19
38.04
33.43
28.64
22.48
18.15
14.28
12.11
EtO
O
N
O
Me
Bn
TIPSO
Major
Minor
d.r. 99:1
f1 (ppm)

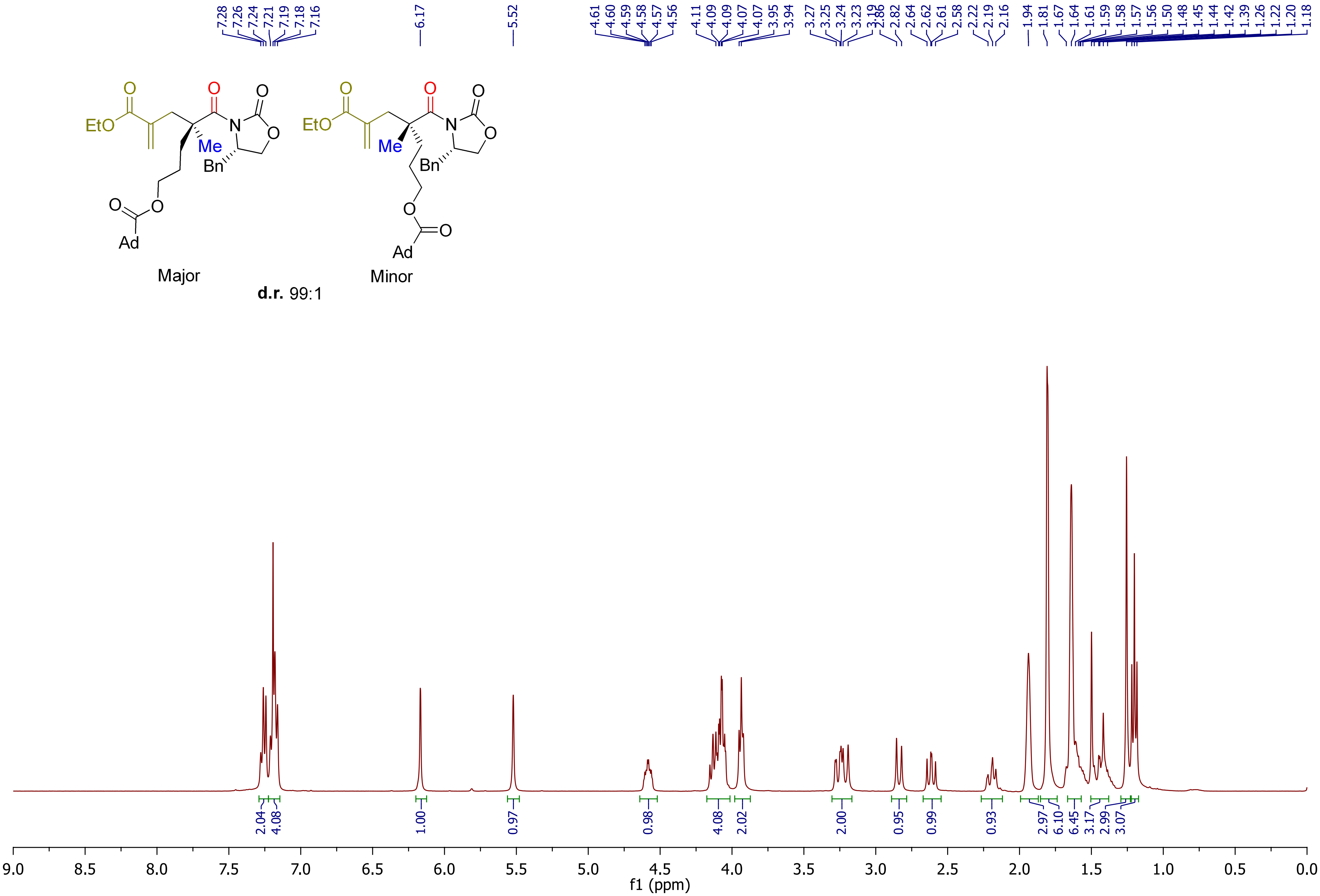

Major
Minor
d.r. 99:1
7.28 7.26 7.24 7.21 7.19 7.18 7.16
6.17
5.52
4.61 4.60 4.59 4.58 4.57 4.56
4.11 4.09 4.09 4.07 4.07 3.95 3.94
3.27 3.25 3.24 3.23 3.19 2.86 2.82 2.64 2.62 2.61 2.58
2.22 2.19 2.16
1.94 1.81 1.67 1.64 1.61 1.59 1.58 1.57 1.56 1.50 1.48 1.45 1.44 1.42 1.39 1.26 1.22 1.20 1.18
2.04 4.08 1.00 0.97 0.98 4.08 2.02 2.00 0.95 0.99 0.93 2.97 6.10 6.45 3.17 2.99 3.07
9.0 8.5 8.0 7.5 7.0 6.5 6.0 5.5 5.0 4.5 4.0 3.5 3.0 2.5 2.0 1.5 1.0 0.5 0.0
f1 (ppm)

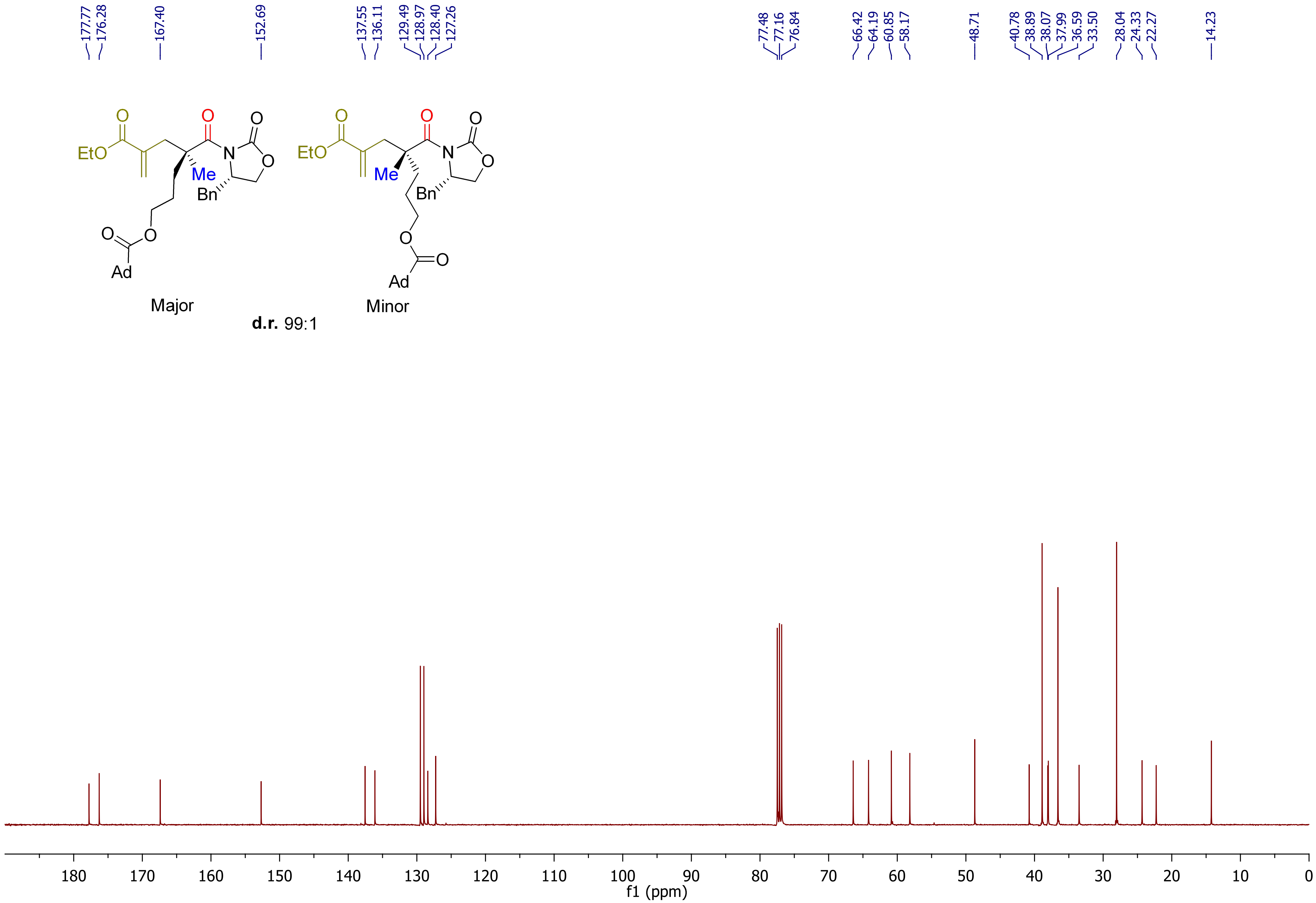
177.77
176.28
167.40
152.69
137.55
136.11
129.49
128.97
128.40
127.26
77.48
77.16
76.84
66.42
64.19
60.85
58.17
48.71
40.78
38.89
38.07
37.99
36.59
33.50
28.04
24.33
22.27
14.23
EtO
Me
Bn
Ad
Major
d.r. 99:1
Minor
180 170 160 150 140 130 120 110 100 90 80 70 60 50 40 30 20 10 0
f1 (ppm)

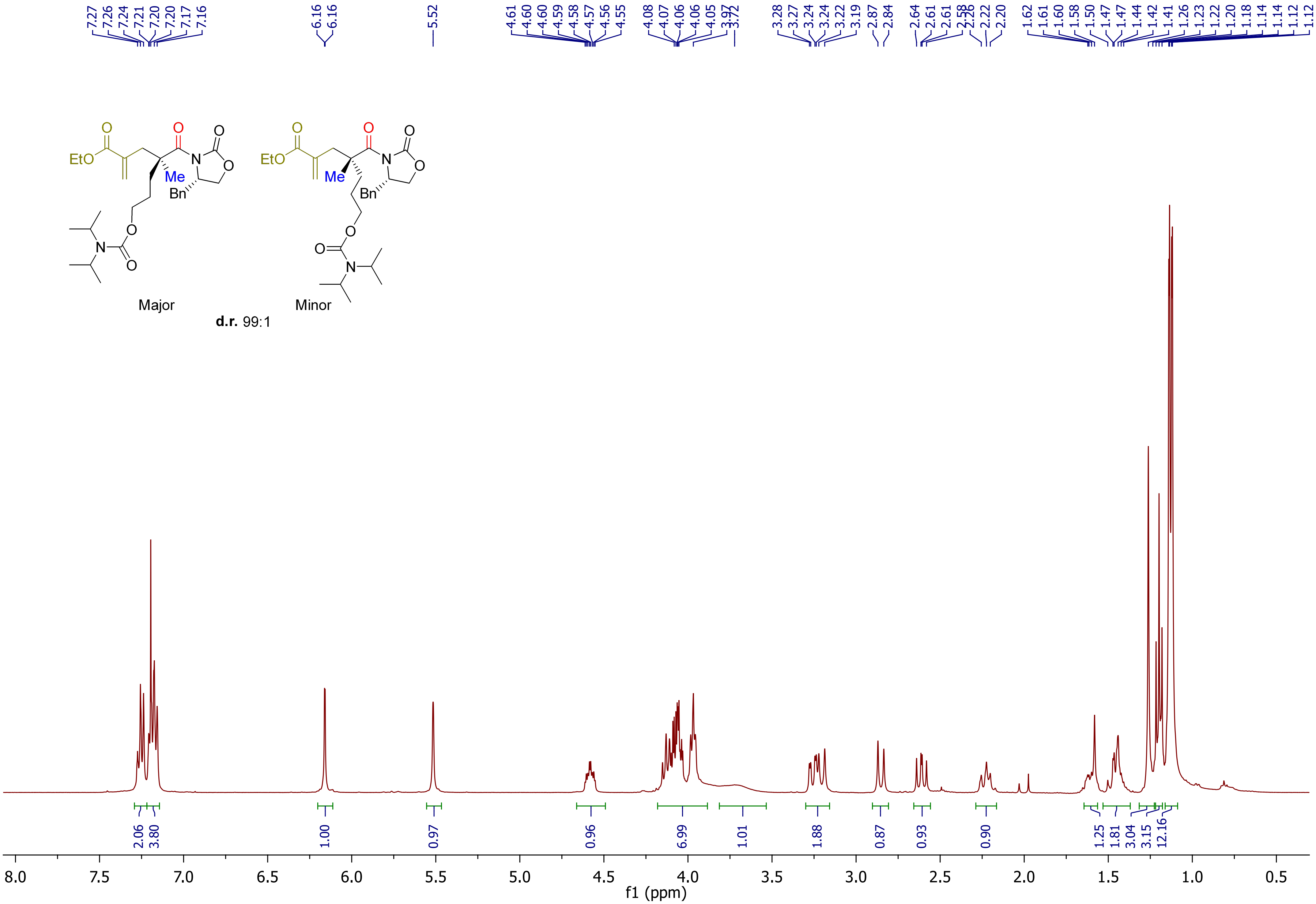

7.27 7.26 7.24 7.21 7.20 7.20 7.17 7.16
6.16 6.16
5.52
4.61 4.60 4.60 4.59 4.58 4.57 4.56 4.55
4.08 4.07 4.06 4.06 4.05 3.97 3.72
3.28 3.27 3.24 3.24 3.22 3.19
2.87 2.84
2.64 2.61 2.61 2.58 2.26 2.22 2.20
1.62 1.61 1.60 1.58 1.50 1.47 1.47 1.44 1.42 1.41 1.26 1.23 1.22 1.20 1.18 1.14 1.14 1.12 1.12
EtO
O
Me
N
Bn
O
Major
Minor
d.r. 99:1
2.06 3.80 1.00 0.97 0.96 6.99 1.01 1.88 0.87 0.93 0.90 1.25 1.81 3.04 3.15 12.16
8.0 7.5 7.0 6.5 6.0 5.5 5.0 4.5 4.0 3.5 3.0 2.5 2.0 1.5 1.0 0.5
f1 (ppm)

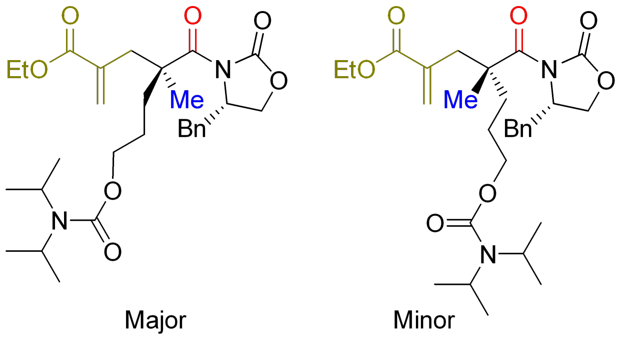
EtO
Me
N
O
Bn
Major
EtO
Me
N
O
Bn
Minor

d.r. 99:1

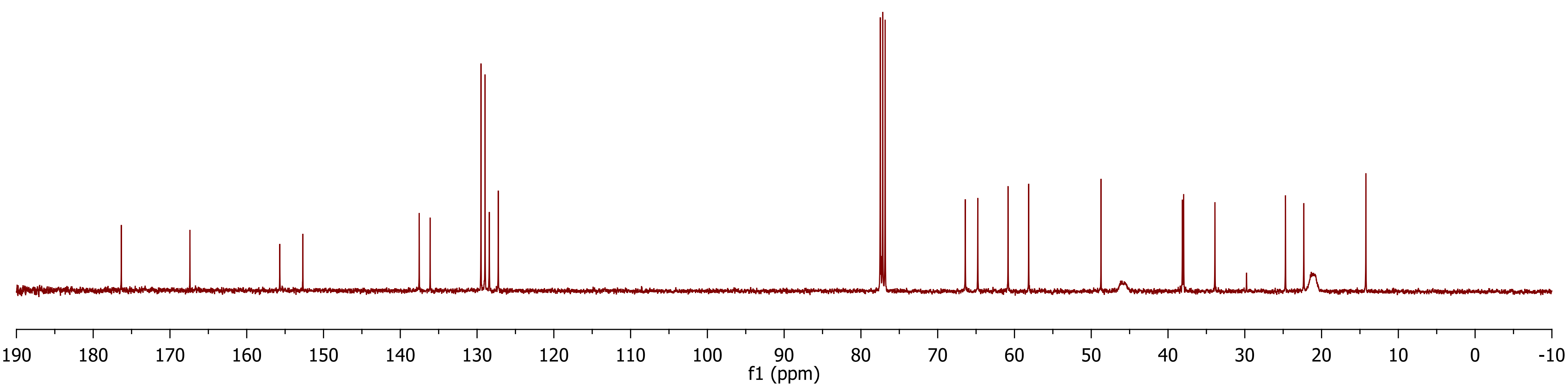
176.34
167.40
155.70
152.69
137.53
136.11
129.49
128.96
128.40
127.23
77.48
77.16
76.84
66.41
64.78
60.83
58.15
48.72
46.12
38.13
37.96
33.89
24.70
22.31
20.88
14.23
190
180
170
160
150
140
130
120
110
100
90
80
70
60
50
40
30
20
10
0
-10
f1 (ppm)

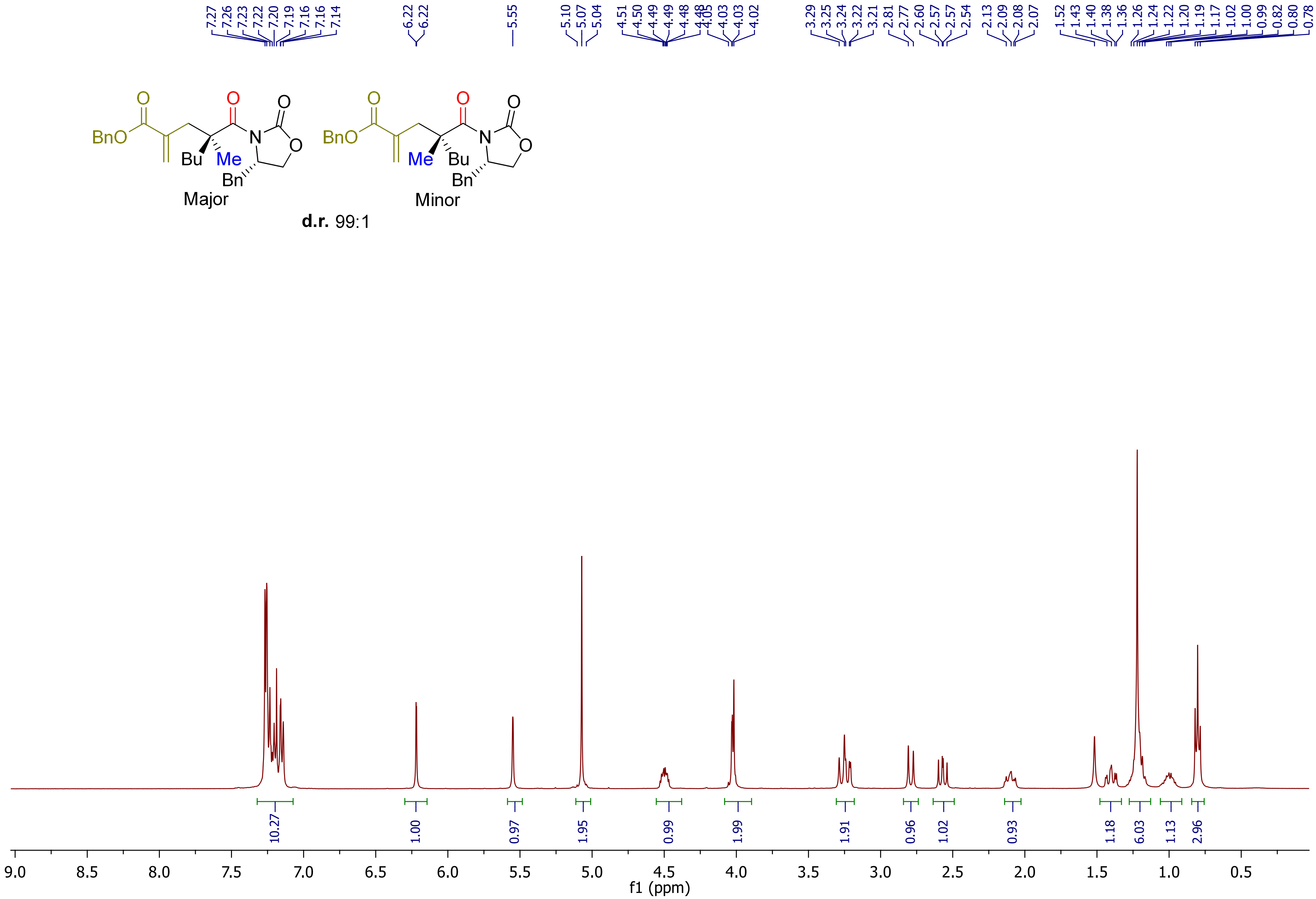
7.27
7.26
7.23
7.22
7.20
7.19
7.16
7.16
7.14
6.22
6.22
5.55
5.10
5.07
5.04
4.51
4.50
4.49
4.49
4.48
4.48
4.05
4.03
4.03
4.02
3.29
3.25
3.24
3.22
3.21
2.81
2.77
2.60
2.57
2.57
2.54
2.13
2.09
2.08
2.07
1.52
1.43
1.40
1.38
1.36
1.26
1.24
1.22
1.20
1.19
1.17
1.02
1.00
0.99
0.82
0.80
0.78
BnO
O
O
O
N
O
Bu
Me
Bn
Major
Me
Bu
Minor
d.r. 99:1
10.27
1.00
0.97
1.95
0.99
1.99
1.91
0.96
1.02
0.93
1.18
6.03
1.13
2.96
9.0
8.5
8.0
7.5
7.0
6.5
6.0
5.5
5.0
4.5
4.0
3.5
3.0
2.5
2.0
1.5
1.0
0.5
f1 (ppm)

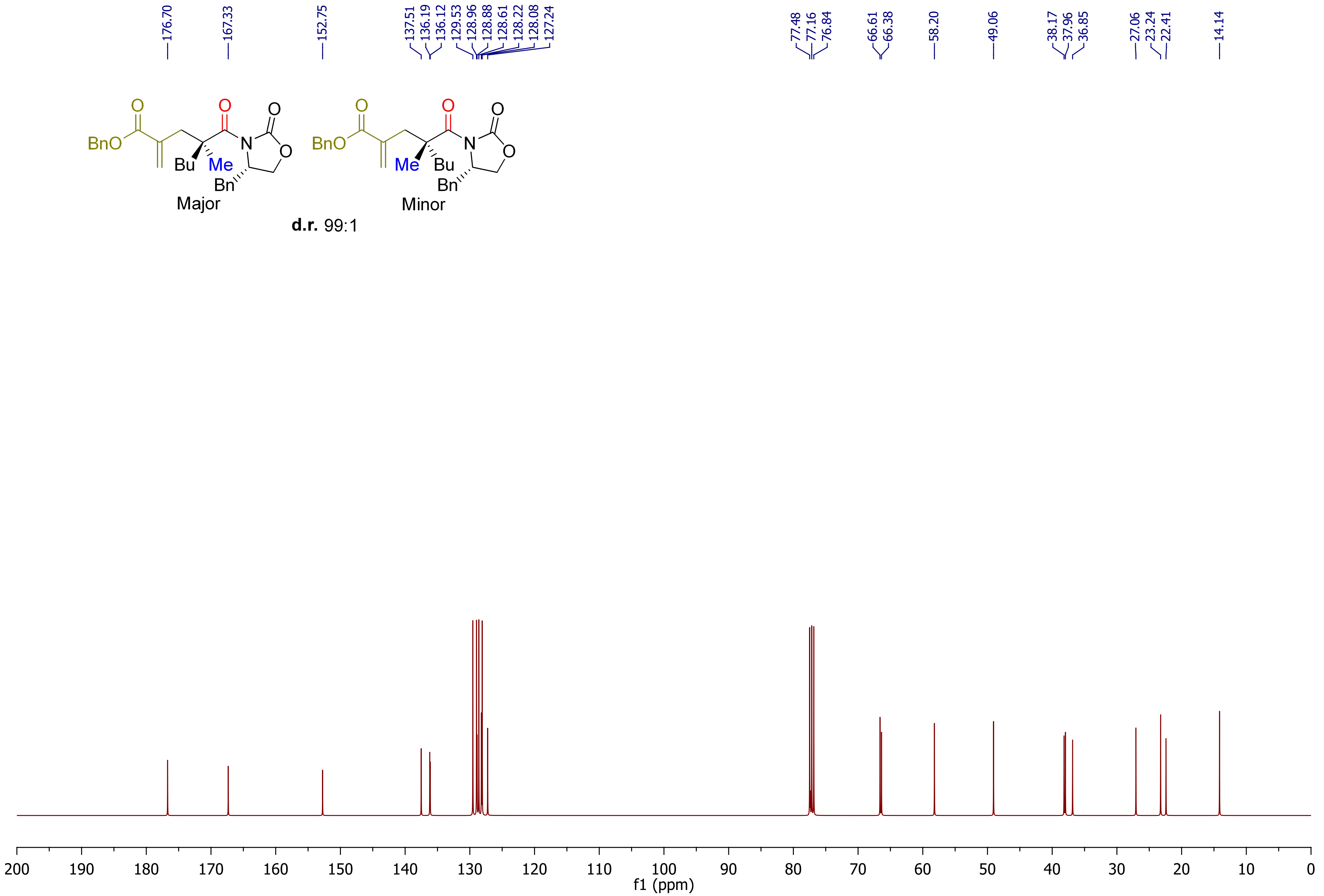
176.70
167.33
152.75
137.51
136.19
136.12
129.53
128.96
128.88
128.61
128.22
128.08
127.24
77.48
77.16
76.84
66.61
66.38
58.20
49.06
38.17
37.96
36.85
27.06
23.24
22.41
14.14
BnO
Bu
Me
N
O
Bn
Major
d.r. 99:1
Minor
200
190
180
170
160
150
140
130
120
110
100
90
80
70
60
50
40
30
20
10
0
f1 (ppm)

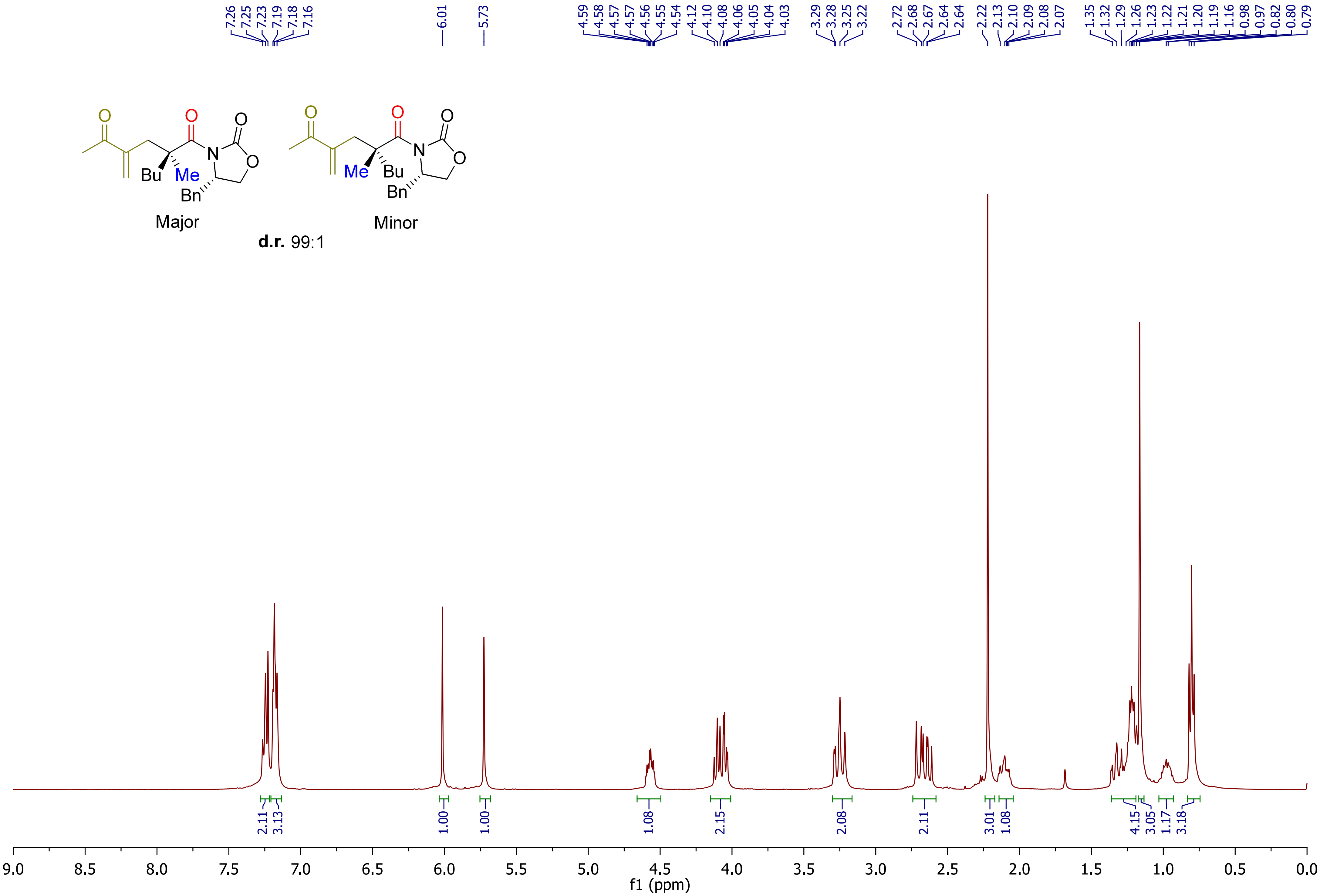

7.26 7.25 7.23 7.19 7.18 7.16
6.01
5.73
4.59 4.58 4.57 4.57 4.56 4.55 4.54 4.12 4.10 4.08 4.06 4.05 4.04 4.03
3.29 3.28 3.25 3.22
2.72 2.68 2.67 2.64 2.64 2.22 2.13 2.10 2.09 2.08 2.07
1.35 1.32 1.29 1.26 1.23 1.22 1.21 1.20 1.19 1.16 0.98 0.97 0.82 0.80 0.79
O
O
O
N
O
Bu
Me
Bn
Major
d.r. 99:1
Me
Bu
Bn
Minor
2.11 3.13 1.00 1.00 1.08 2.15 2.08 2.11 3.01 1.08 4.15 3.05 1.17 3.18
9.0 8.5 8.0 7.5 7.0 6.5 6.0 5.5 5.0 4.5 4.0 3.5 3.0 2.5 2.0 1.5 1.0 0.5 0.0
f1 (ppm)

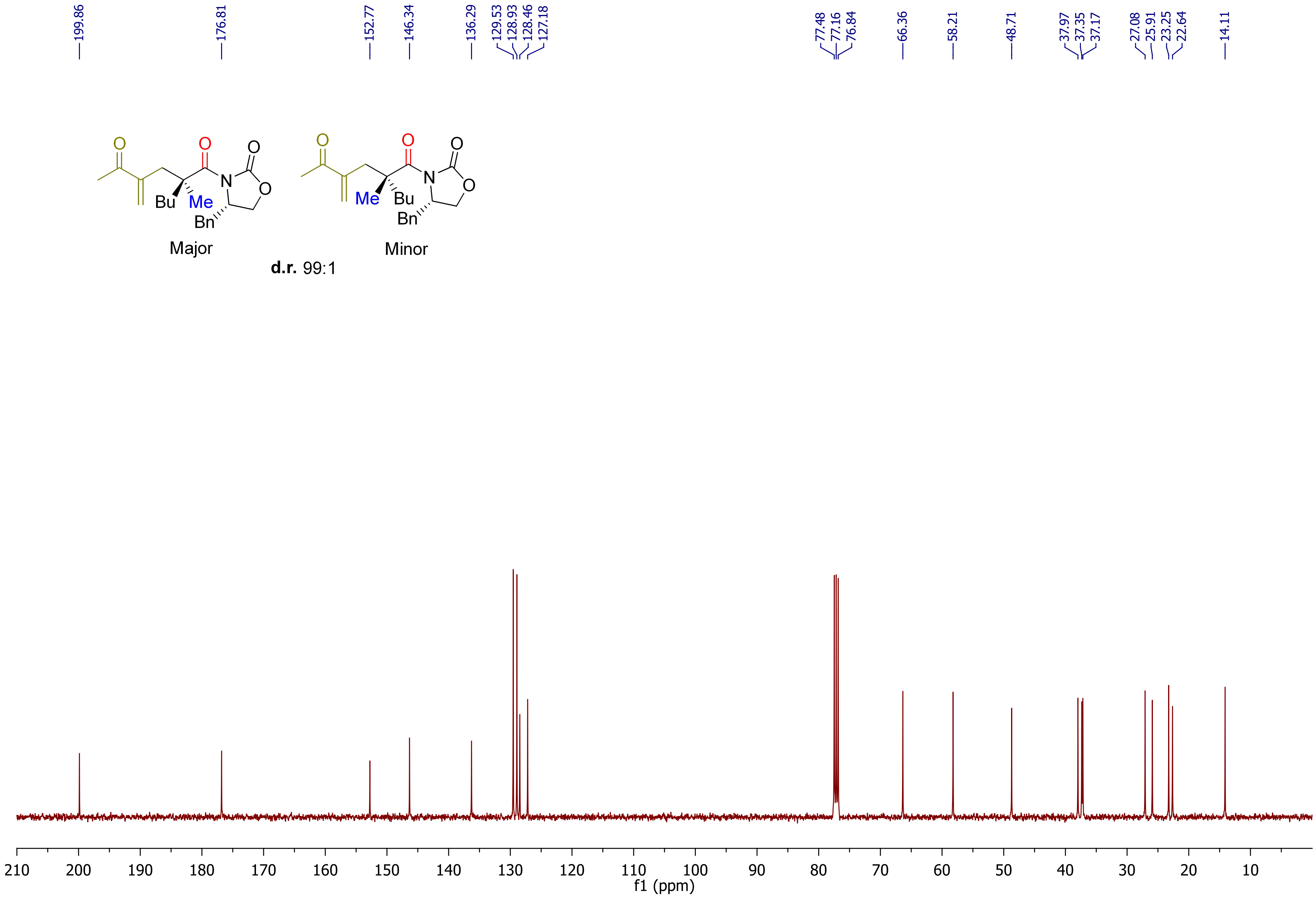
199.86
176.81
152.77
146.34
136.29
129.53
128.93
128.46
127.18
77.48
77.16
76.84
66.36
58.21
48.71
37.97
37.35
37.17
27.08
25.91
23.25
22.64
14.11
Major
Minor
d.r. 99:1
210 200 190 180 170 160 150 140 130 120 110 100 90 80 70 60 50 40 30 20 10
f1 (ppm)

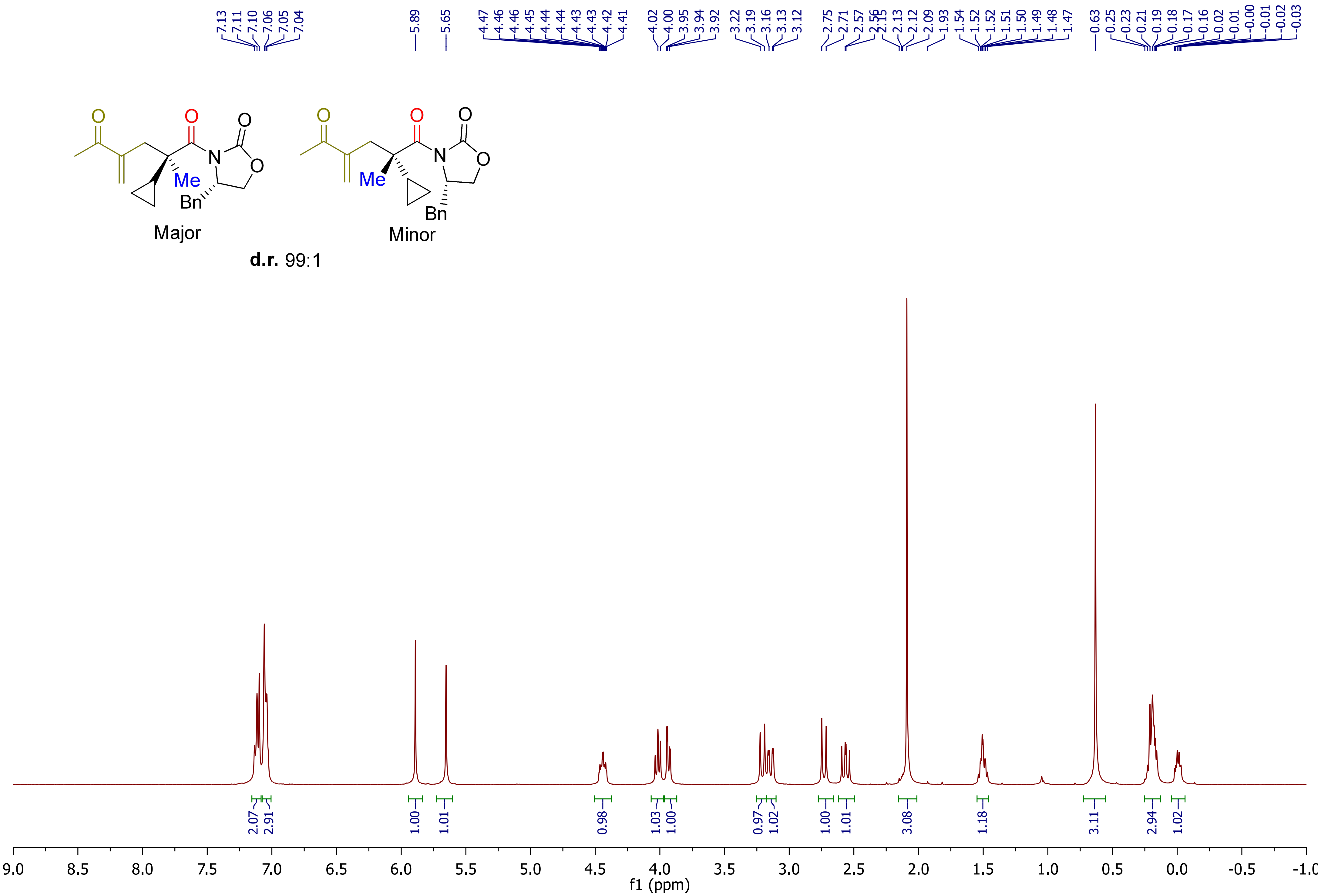
Major
Minor
d.r. 99:1
f1 (ppm)

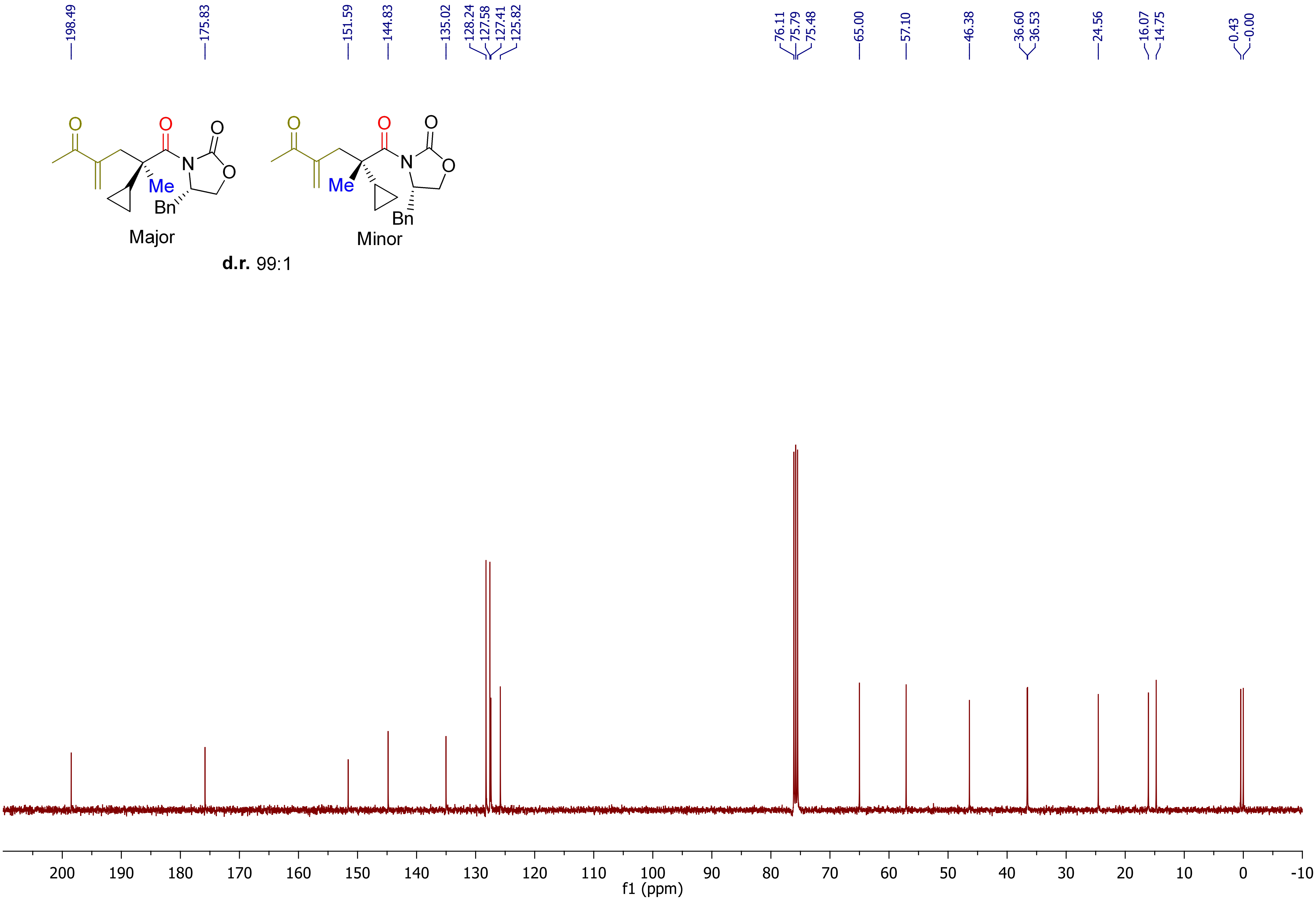
198.49
175.83
151.59
144.83
135.02
128.24
127.58
127.41
125.82
76.11
75.79
75.48
65.00
57.10
46.38
36.60
36.53
24.56
16.07
14.75
0.43
-0.00
O
O
N
O
O
Me
Bn
Major
O
O
N
O
O
Me
Bn
Minor
d.r. 99:1
200
190
180
170
160
150
140
130
120
110
100
90
80
70
60
50
40
30
20
10
0
-10
f1 (ppm)

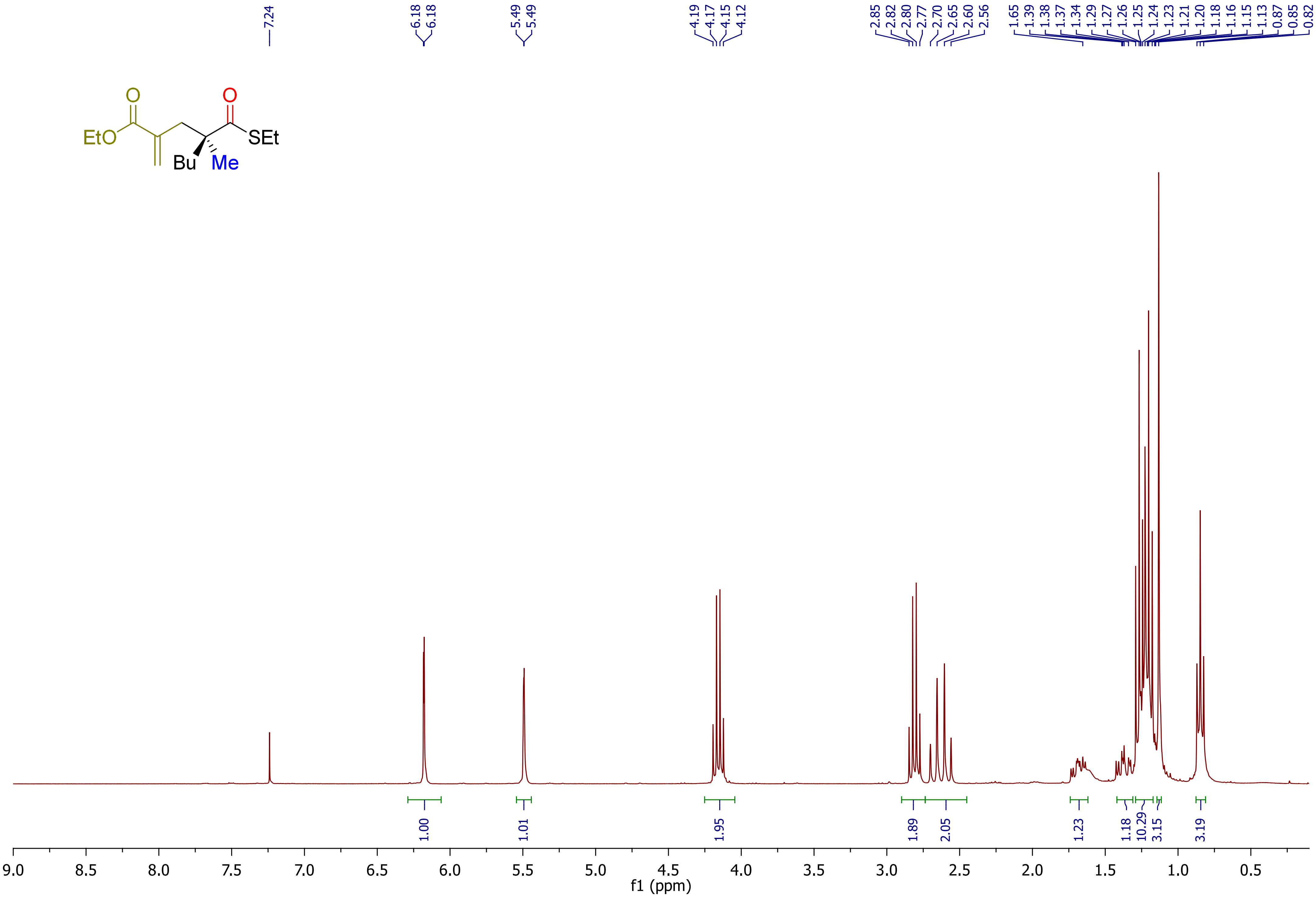

7.24
6.18
6.18
5.49
5.49
4.19
4.17
4.15
4.12
2.85
2.82
2.80
2.77
2.70
2.65
2.60
2.56
1.65
1.39
1.38
1.37
1.34
1.29
1.27
1.26
1.25
1.24
1.23
1.21
1.20
1.18
1.16
1.15
1.13
0.87
0.85
0.82
EtO
O
O
SEt
Bu
Me
1.00
1.01
1.95
1.89
2.05
1.23
1.18
10.29
3.15
3.19
9.0
8.5
8.0
7.5
7.0
6.5
6.0
5.5
5.0
4.5
4.0
3.5
3.0
2.5
2.0
1.5
1.0
0.5
f1 (ppm)

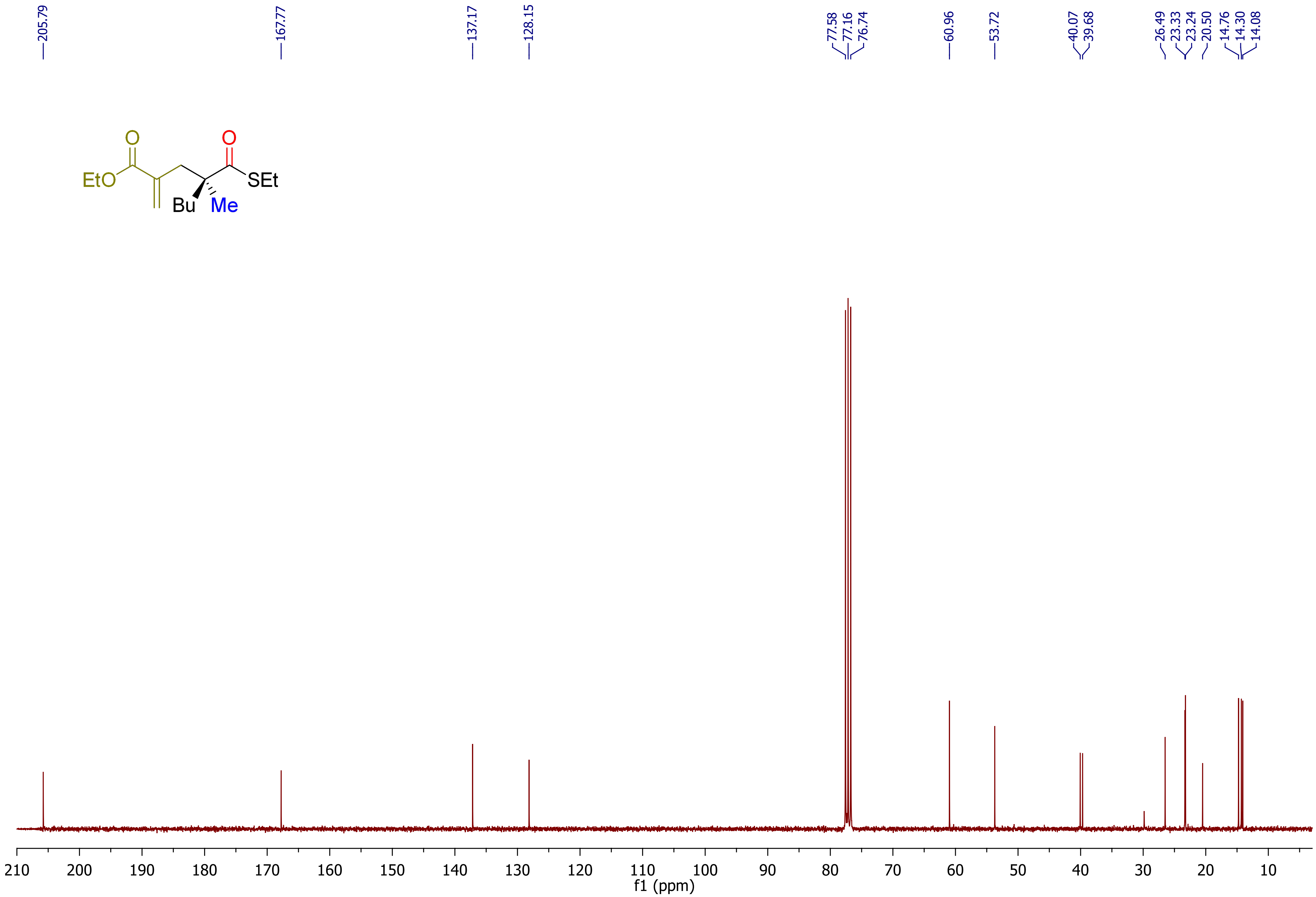
205.79
167.77
137.17
128.15
77.58
77.16
76.74
60.96
53.72
40.07
39.68
26.49
23.33
23.24
20.50
14.76
14.30
14.08
EtO
O
O
SEt
Bu
Me
210 200 190 180 170 160 150 140 130 120 110 100 90 80 70 60 50 40 30 20 10
f1 (ppm)

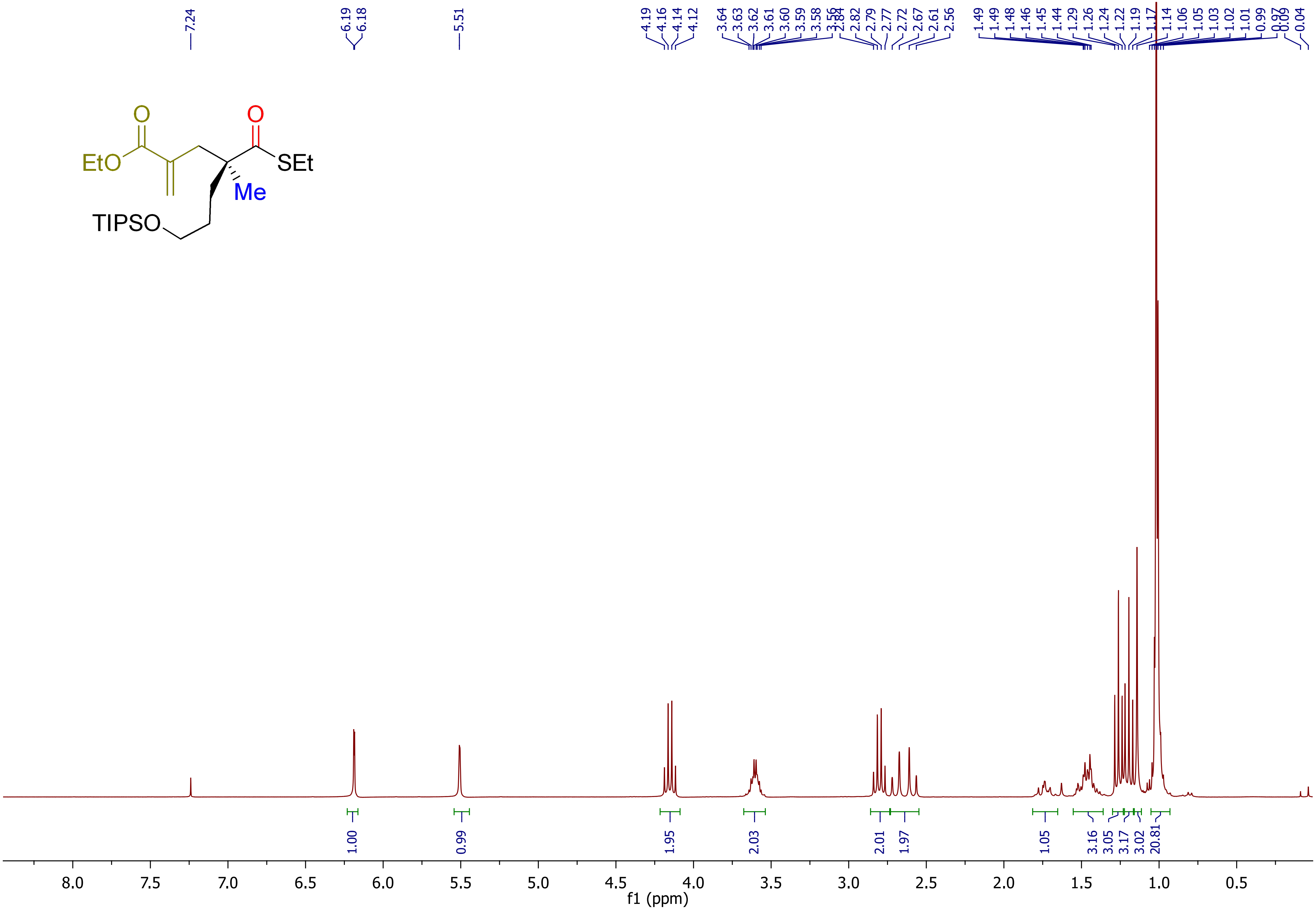
EtO
O
O
SEt
Me
TIPSO
f1 (ppm)

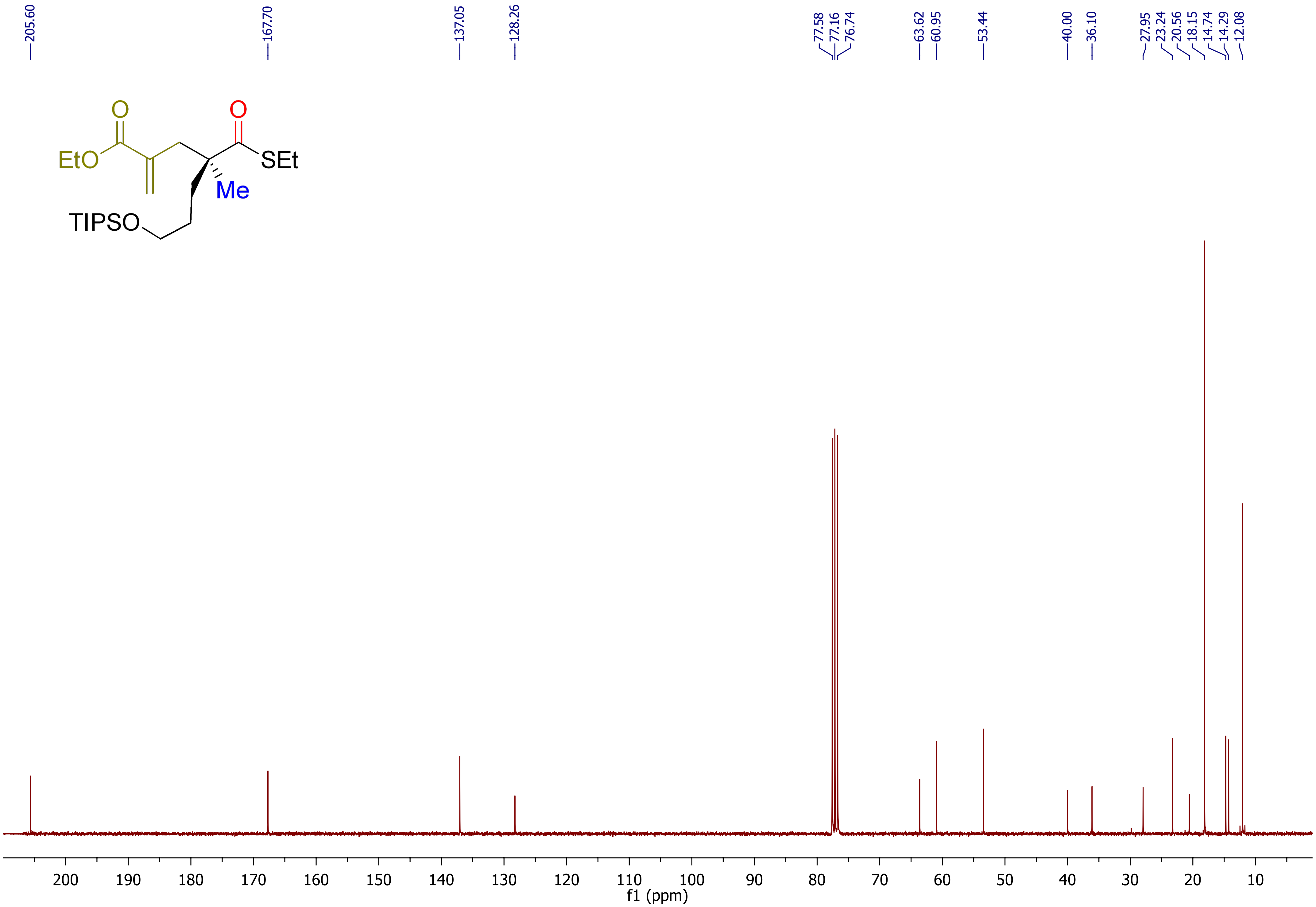
205.60
167.70
137.05
128.26
77.58
77.16
76.74
63.62
60.95
53.44
40.00
36.10
27.95
23.24
20.56
18.15
14.74
14.29
12.08
EtO
O
O
SEt
Me
TIPSO
200
190
180
170
160
150
140
130
120
110
100
90
80
70
60
50
40
30
20
10
f1 (ppm)
